# Diffusion-RarePHM: A Physics-Constrained Conditional Latent World Model for Multi-Future Degradation Forecasting and Rare-Fault Prognostics

**DOI:** 10.3390/s26216785

**Published:** 2026-10-27

**Authors:** Yuan Yao, Weiqiang Mao, Binghua Su

**Affiliations:** 1 Faculty of Data Science, City University of Macau, Macao 999078, China; yao_yuan@bitzh.edu.cn; 2 Marine Science and Technology Domain, Beijing Institute of Technology, Zhuhai 519088, China; 3 Department of Computing, The Hongkong Polytechnic University, Hong Kong, China; weiqiang.mao@hkpolyu-njresearch.cn; 4 Frontier Interdisciplinary Domain, Beijing Institute of Technology, Zhuhai 519088, China

**Keywords:** conditional latent diffusion, rare-fault prognostics, multi-future degradation, physics-constrained world model, uncertainty-aware remaining useful life

## Abstract

Industrial prognostics is constrained less by the abundance of healthy telemetry than by the scarcity, heterogeneity, and operational ambiguity of genuine failure evolution. This study introduces Diffusion-RarePHM, a physics-constrained conditional latent world model that converts heterogeneous monitoring streams into probabilistic, multi-future degradation scenarios. A unified health encoder maps engine and compressor observations into a common mechanism-oriented health interface with asset-specific fitting, while state-dependent dual-domain dynamics couple latent health levels with their increments. Conditioned on current health, equipment structure, prospective duty, environment, maintenance action, and target fault mode, latent diffusion generates an ensemble of complete future trajectories. Rare precursors are synthesized as physically directed departures from normal latent evolution and screened by physical, dynamic, and explicit temporal-predicate semantic criteria. The study draws from PHM2024 and MetroPT3 source archives containing 742,625 and 1,516,948 records, respectively; deterministic PHM2024 subsets and 12,642 chronologically retained MetroPT3 observations are used for the reported experiments. On the PHM2024 record-level held-out diagnosis partition, the model attained 98.23% accuracy, 98.16% macro-F1, and 0.9985 AUROC. Across held-out operating-condition groups, it attained 97.86±0.12% accuracy, 97.77±0.13% macro-F1, and 0.9979±0.0003 AUROC over five training seeds. In the MetroPT3-anchored controlled semi-physical benchmark, reconstruction of prescribed health trajectories achieved nine-scenario averages of 2.250 percentage-point RMSE, 2.294% MAPE, and 90.00% pointwise constructed-reference coverage for the nominal 90% interval. Controlled trajectory experiments examine the generator’s response to prescribed precursors, leakage, compound degradation, maintenance, thermal-load stress, and observation corruption, together with their first-passage and tail-risk functionals. The trajectory evidence establishes mechanism-conditioned generation and reconstruction of controlled degradation scenarios, with accepted-path warning, first-passage, and tail-risk statistics derived under the specified dynamics and interventions.

## 1. Introduction

Industrial telemetry describes nominal operation far more extensively than the evolution of failure. In turbines and compressors, the evidence for deterioration is intertwined with load, environment, controller switching, and maintenance. The prognostic target consequently includes both accumulated condition [1,2,3,4] and the operating response through which that condition is observed. Physics-informed degradation models and hybrid model-based predictors preserve mechanistic structure under partial observations [5,6,7,8,9,10], while few-shot, recurrent, and attention-based methods capture interactions among monitoring channels [11,12,13,14]. These developments provide the state-estimation basis [15] for reasoning about several plausible futures from the same present evidence.

Alternative futures differ in timing and mechanism as well as dispersion. A similar current health state may precede gradual decline, accelerated deterioration, or maintenance-mediated recovery, with different warning and loss consequences. Probabilistic RUL estimation [16,17,18,19] and recurrent distributional forecasting [20,21] characterize uncertainty, while ensemble and conformalized methods support interval assessment [22,23]. Diffusion forecasters represent correlated future sequences [24,25,26]; conditional and temporally encoded generators further preserve long-range structure [27,28,29,30]. For industrial prognosis, this distributional representation must also respond to prospective operating conditions and interventions, so the timing of threshold crossing remains linked to the path that produces it.

Rare-fault evidence adds a complementary requirement: generation must preserve the transition from precursor to developed fault. Open-set diagnosis and domain-generalized representations improve recognition under unfamiliar conditions [31,32,33,34,35,36,37,38,39,40,41,42]. Generative adaptation, unknown-condition augmentation, and open-space feature synthesis broaden the diagnostic population [43,44,45,46], while compound-fault and imbalance-aware learning account for interacting mechanisms and uneven class frequencies [47,48,49,50,51,52]. Extending these ideas to prognosis requires control over onset, accumulated displacement, and event order within complete trajectories. A direction fitted in the health-state space can supply this control, while residual generation represents variation around the specified mechanism.

Physical consistency concerns both individual states and their temporal connections. Structure-aware, graph-based, and conditional-density anomaly detectors resolve coordinated departures among sensors [53,54,55], while masked latent and multiscale approaches characterize the geometry and persistence of these departures [56,57,58]. Mechanism-informed fault models and digital-twin maintenance relate learned responses to equipment operation [59,60]. Yang et al. similarly place mechanistic–data-driven fusion within state estimation and control under variable operating conditions [61]. For generated degradation paths, reconstructed channel relations [62,63], transition residuals, and control-response order therefore provide complementary evidence about whether the future remains compatible with the queried asset and intervention.

Diffusion-RarePHM formulates these requirements as conditional distribution learning over complete latent futures. Asset-specific projections map heterogeneous observations into a common mechanism-oriented health interface. State-dependent dual-domain dynamics (SDA2D) couple the latent level with its increment, distinguishing persistent displacement from reversible operating motion. Conditional latent diffusion then generates alternative paths under duty, environment, maintenance, and fault specifications. Physics-directed displacement supplies the rare-fault profile, and physical, dynamic, and explicit temporal-predicate gates define the accepted path population. Contextual semantic verification is motivated by time-series vision-language research [64] and implemented through declared event-order predicates.

The resulting formulation contributes a condition-responsive law for complete futures, a mechanism-directed construction for rare-fault scenarios, and a complementary three-criterion validation rule. Warning probability, first-passage RUL, prediction intervals, and tail loss are derived from the same accepted ensemble, preserving the relationship between event timing and adverse severity. PHM2024 fault-label diagnosis evaluates the engine representation; MetroPT3 histories support compressor transition fitting, controlled scenario generation, and health-trajectory reconstruction. Their separate targets make representation quality, future-path fidelity, and decision statistics distinguishable within a common inference framework.

## 2. Dataset Description

### 2.1. Overview of Datasets

The empirical study combines two complementary industrial regimes. PHM2024 supplies a large labeled operating envelope for turbine-engine health discrimination, whereas MetroPT3 provides a continuous compressor record in which pressure, temperature, current, and controller states evolve jointly. PHM2024 therefore supports supervised diagnosis across broad environmental and load conditions, while MetroPT3 supports trajectory learning, dynamic consistency, and condition-controlled counterfactual analysis. Robot and battery variable schemas additionally inform energy-action and long-term capacity constraints for cross-asset extension.

The PHM2024 training archive contains 742,625 observations. Each record comprises measured torque, outside-air temperature, measured gas temperature, pressure altitude, indicated airspeed, power-turbine speed, and gas-generator speed. The associated targets include a binary fault indicator and continuous torque margin. The dataset is physically meaningful because torque margin is not an arbitrary statistical label: it expresses the residual capability between demanded and available engine torque after correcting for atmospheric density and operating condition. The official challenge analysis relates health estimation to torque margin and density altitude [65]. Faulty observations therefore occupy a condition-dependent region rather than a single global threshold in raw sensor space.

MetroPT3 contains 1,516,948 records from 1 February to 1 September 2020, sampled approximately every 10 s. The selected channels include TP2, TP3, H1, discharge-valve pressure, reservoir pressure, oil temperature, motor current, compressor command, electric discharge-valve state, tower state, MPG, low-pressure switch, pressure switch, oil level, and flow impulses. Its data paper documents the asset, acquisition process, and maintenance relevance [66]. The compressor is particularly suitable for physics-constrained generation because analog pressures and thermal states must remain compatible with binary actuation logic. For example, compressor loading raises current and changes the relationship among TP2, TP3, H1, and reservoir pressure; a synthetic path that breaks these couplings is statistically plausible but physically inadmissible.

### 2.2. Degradation Mechanisms and Physical Significance

Battery and robot telemetry define extension constraints for electrochemical and electromechanical assets. BatteryLife standardizes cycling, capacity, voltage, current, temperature, material, and protocol metadata across multiple sources [67], motivating monotone long-term capacity constraints conditioned on chemistry and duty. Seven-axis robot measurements of speed, torque, angle, power, voltage, and current motivate compatibility constraints among commanded motion, joint effort, and energy consumption. Quantitative evaluation remains confined to PHM2024 and MetroPT3, for which the complete diagnostic and trajectory evidence is available.

### 2.3. Final Dataset Specification and Evidential Scope

The public PHM2024 training release pools observations from four engines of the same make and model; the organizers shuffled the observations and removed asset identifiers before release. The official test and validation datasets draw on the remaining three engines [68]. Subset extraction uses a four-row stride and stops after 180,000 records. Filtering missing labels and missing feature values retains 179,412 records. A label-stratified sample without replacement, using seed 2024, selects 150,000 classification records comprising 90,000 nominal and 60,000 faulty observations. The 75%/25% record-level partition is generated with train_test_split, stratify=label, and random_state=2024. Model development contains 67,500 nominal and 45,000 faulty records; the internal held-out set contains 22,500 nominal and 15,000 faulty records. The partition manifest records original row identity, subset row identity, partition membership, and label. These row identities index the shuffled release, whereas physical sequence information resides in the original operating records.

An additional operating-condition-held-out evaluation isolates environmental and operating regimes. Robustly standardized OAT, pressure altitude, IAS, NP, and NG define ten K-means groups, indexed 0–9, with clustering seed 2026 and 20 initializations. The group-standardization parameters and centroids are fitted on all 150,000 sampled records using these operating covariates. Groups {3,7} form the primary held-out set; groups {0,1,2,4,5,6,8,9} form the development. Classifier preprocessing and optimization then use development records. The initial partition contains 119,750 development and 30,250 held-out records. A Mahalanobis-distance exclusion region removes 374 held-out observations using input geometry, yielding 29,876 evaluation records. Table A7 reports the resulting diagnosis performance, and Appendix B specifies the partition and fitting procedure.

MetroPT3 is retained every 120 records, yielding 12,642 observations while preserving the seven-month chronology on the declared trajectory grid. Table 1 summarizes the resulting evidential scope and analytical role of each dataset. Robust medians and median absolute deviations are used for normalization.

The trajectory benchmark couples retained MetroPT3 measurements with condition-controlled future interventions. Six consecutive retained observations form a trajectory block, using the mean of continuous channels and the last value of binary controls, yielding 2107 positions at a nominal two-hour resolution. The history window contains L=64 blocks (128 h), and the forecast extends over H=160 blocks (320 h). The 20 min retained grid supports the compressor chronology, whereas the two-hour grid defines the trajectory experiments. Measured observations anchor the forecast-origin state and local operating variation; scenario-defined health paths constitute the reference trajectories. Health is expressed on a percentage scale, with larger values denoting healthier states.

The normal reference follows a deterministic SDA2D mean rollout, with the increment evaluated as $\Delta z_{t}=z_{t}-z_{t-1}$. Prescribed fault and maintenance interventions define departures from this normal path. Sensor drift and modality masking act on the observation stream while retaining the same mechanical reference under a common initial state and duty condition. Reference construction does not invoke reverse diffusion, the triple-gate validator, or the calibration objective. At each forecast origin, the model proposes 500 candidate futures per seed; five seeds, 2026–2030, provide repeated stochastic realizations. Predictive statistics are evaluated on the accepted ensemble.

The chronological partition assigns blocks 0–421 to normal representation and SDA2D fitting, blocks 422–843 to gate and interval-calibration settings, and blocks 844–2106 to final trajectory evaluation. Evaluation uses the 256 distinct origins $t_{i}=908+4i$, i=0,…,255, with all 64 history blocks and 160 future blocks contained in the evaluation segment. Consecutive origins are eight hours apart and share 60 history blocks and 156 future time positions. The evaluation thus retains temporal dependence among rolling origins. Scenario interventions and repeated seeds reuse these origin histories; their candidate paths sample conditional futures at each origin.

Calibration uses 128 distinct normal-condition origins $o_{j}=485+\mathrm{round}\left(198j/127\right)$, j=0,…,127. Their complete history–forecast supports lie within blocks 422–843: the first history starts at block 422, and the last forecast ends at block 843. Source-window-disjoint evaluation cases are identified at origins 908, 1132, 1356, 1580, and 1804, with respective supports 845–1068, 1069–1292, 1293–1516, 1517–1740, and 1741–1964. This five-case subset is selected by source indices; the complete rolling-origin evaluation retains all 256 origins. These five supports are chronological windows within one MetroPT3 compressor record. Prescribed scenarios reuse the recorded history under different intervention laws, and training seeds characterize algorithmic variation on common data; the number of independently recorded equipment histories remains one.

The benchmark and predictive model share the health mapping, temporal grid, prescribed intervention profiles, fault directions, and SDA2D mean transition structure. These shared mechanisms define the reference against which stochastic generation, admissibility screening, and uncertainty calibration are assessed. RMSE and MAPE quantify agreement with the constructed health evolution under the declared scenario, while interval coverage measures the inclusion of its reference path within the predictive ensemble. The PHM2024 diagnosis metrics separately quantify discrimination against measured fault labels.

Appendix A specifies the health mapping, intervention laws, reference construction, and ensemble evaluation. The shared-operator deterministic rollout in Equation (A10a) reproduces the constructed target. Measured MetroPT3 records supply the initial histories and normal-transition fit; the evaluated future health paths follow the prescribed scenario laws. Trajectory error and pointwise interval coverage assess reconstruction and reference inclusion within this controlled system.

## 3. Method

**Figure 1:**
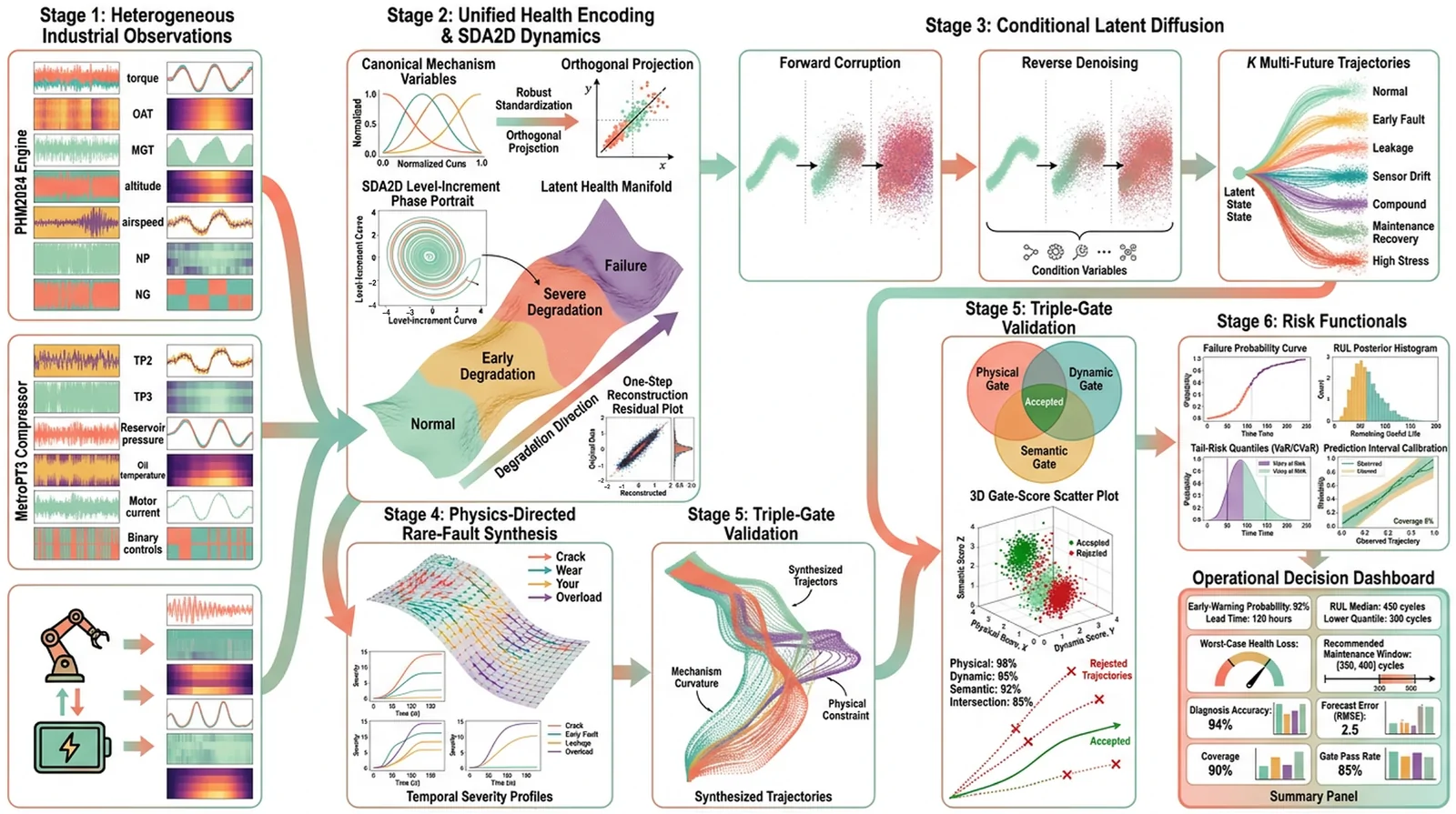
End-to-end architecture of Diffusion-RarePHM. Heterogeneous industrial observations are transformed through unified health encoding, SDA2D latent dynamics, conditional latent diffusion, physics-directed rare-fault synthesis, risk functionals, and the staged training procedure.

The architecture in Figure 1 organizes prognostic inference around complete conditional futures. The present state constrains the starting point, while duty, environment, maintenance, and fault mode determine the evolution being queried. Level–increment dynamics retain the operating response within that evolution, and diffusion represents variation among paths compatible with the same history. Directed displacement supplies the prescribed degradation mechanism; the gates determine which realizations enter decision statistics. This path-based organization connects the timing of deterioration with its eventual severity, so warning, RUL, and tail loss retain a common probabilistic origin.

### 3.1. Problem Formulation and Notation

At decision time *t*, the model receives an observation window $H_{t}$, contextual condition $c_{t}$, and an optional target-fault condition. Equation (1) defines the conditional law of the complete future health sequence $Y_{t+1:t+H}$ over horizon *H* by integrating the decoder $p_{\theta }$ over the latent future path Z drawn from $p_{\varphi }$ given $H_{t}$ and $c_{t}$. RUL distribution, prediction intervals, VaR, and CVaR use the gate-conditioned version of this future law defined in Equation (16); present-state diagnosis reads the encoded observations through Equation (3).

(1) $$p_{\theta }\left(Y_{t+1:t+H}|H_{t},c_{t}\right)=\int p_{\theta }\left(Y|Z,c_{t}\right)p_{\varphi }\left(Z|H_{t},c_{t}\right)\;dZ$$

Equation (2) maps the observation window $x_{t-L+1:t}$, its local increment $\Delta x_{t}$, and context $c_{t}$ through the asset-fitted instance of the common encoder formulation $E_{\varphi }$ to the latent health state $z_{t}$. The window length *L* controls the historical support used for state inference. Rather than compressing the record to one scalar, $z_{t}$ retains operating regime, thermal and loading evidence, pressure or effort, and degradation orientation, allowing the same state to support both diagnosis and future generation.

(2) $$z_{t}=E_{\varphi }\left(x_{t-L+1:t},\Delta x_{t},c_{t}\right)$$

Equation (3) applies the diagnosis rule to the shared state: *m* is the current mode, $p_{\omega }\left(m|z_{t},c_{t}\right)$ is its posterior, and the argmax returns the reported mode. Because prognosis conditions on the same $z_{t}$ and $c_{t}$, diagnosis and future generation read the same latent evidence rather than two disconnected feature spaces.

(3) $${\hat{m}}_{t}=\underset{m}{\arg\max}\;p_{\omega }\left(m|z_{t},c_{t}\right)$$

Here, superscript *k* indexes a sampled future, τ denotes diffusion time, *t* denotes physical time, and *H* is the forecast horizon. The condition vector $c_{t}$ contains present health, equipment structure, prospective duty, environment, maintenance action, and requested fault mode. All subsequent constraints act on this explicitly conditioned future distribution, so prognosis, intervals, and risk remain functionals of its gate-conditioned future distribution.

Partial identifiability is handled by retaining the latent-path integral when the same observation window is compatible with several hidden damage states, for example, under censored failure times or incomplete maintenance history. The model therefore preserves multiple evidence-consistent explanations until future trajectories provide enough separation for a threshold decision.

### 3.2. Unified Health Encoding and State-Dependent Dual-Domain Dynamics

Equation (4) robustly transforms mechanism variable $x_{tj}$ using its channel ${\mathrm{median}}_{j}$ and ${\mathrm{MAD}}_{j}$, with ε preventing division by zero and clip bound *b* limiting the influence of rare extremes. Equation (5) then maps the clipped vector ${\tilde{x}}_{t}$ into the eight-dimensional health interface $z_{t}$ through W, with $W^{T}W=I$ enforcing an orthogonal basis. Robust scaling and orthogonal projection are fitted for each asset, preserving its mechanism structure within the common encoding formulation.

(4) $${\tilde{x}}_{tj}=\mathrm{clip}\;\left(\frac{x_{tj}-{\mathrm{med}}_{j}}{1.4826\;{\mathrm{MAD}}_{j}+\varepsilon },-b,b\right)$$

(5) $$z_{t}=W^{T}{\tilde{x}}_{t},\;W^{T}W=I$$

For asset a∈{P,M}, Equation (5) denotes $z_{t}^{\left(a\right)}={\left(W^{\left(a\right)}\right)}^{T}{\tilde{x}}_{t}^{\left(a\right)}$, where P and M identify PHM2024 and MetroPT3. The projection matrices have shapes $W^{\left(P\right)}\in R^{8\times 8}$ and $W^{\left(M\right)}\in R^{15\times 8}$; the PHM2024 input comprises seven measurements and the derived density ratio specified in Appendix C. Channel medians, MAD scales, projection bases, and component endpoints are estimated within each asset’s fitting partition: PHM2024 development records and MetroPT3 blocks 0–421. The common interface consists of the eight-dimensional state format, the scalar readout H=60+18d, its increasing-health orientation, and the conditioning and risk-functional definitions. Component weights and endpoints retain their asset-specific values in Appendix A. Here, cross-domain representation denotes this common interface with asset-specific fitting.

PHM2024 diagnosis encodes each observation as a single feature vector. Its fitted projection $W^{\left(P\right)}$, health encoder, and classification head form an independent diagnosis branch. MetroPT3 uses its own projection $W^{\left(M\right)}$, history encoder, SDA2D, denoiser, fault direction, gates, and interval parameters. The branches share the eight-coordinate state format, health orientation, and architectural definitions; their trainable parameter sets are disjoint. Sequence-level encoding and the SDA2D transition in Equation (6) use the MetroPT3 chronology for trajectory learning, with the compressor transition parameters, innovation covariance, and physical predicates fitted or calibrated on its designated blocks. Within the MetroPT3 scenario comparison, these fitted operators remain common across the declared interventions.

Channel-count adaptation precedes temporal encoding. For an asset with $D_{a}$ mechanism features, $W^{\left(a\right)}\in R^{D_{a}\times 8}$ maps each standardized record to eight coordinates. Consequently, PHM2024 supplies a 1×8 state input, whereas the MetroPT3 64×15 history becomes a 64×8 latent sequence. Sensor correspondence is specified by each asset’s feature dictionary; absent channels are not appended to a common sensor list. Learned embeddings then set the neural feature width. The latent dimension of eight, history-encoder width of 512, and denoiser width of 128 therefore refer to distinct stages of the computation.

The evidence variants in Table 2 retain these projection dimensions. PHM2024 P5 and P6 concatenate one and three auxiliary descriptors, respectively, after the eight-dimensional projection for diagnosis. MetroPT3 evaluates its derived efficiency, control-consistency, and valve–pressure quantities at the health-readout and gate stages; its projected observation sequence remains 64×8. Appendix Asset-Specific Evidence Variables and Masks specifies the descriptor paths and masks.

Equation (6) is the SDA2D transition. $A_{\psi }$ and $B_{\psi }$ map the current latent level $z_{t}$ and increment $\Delta z_{t}$ under context $c_{t}$, $b_{\psi }\left(z_{t},c_{t}\right)$ supplies state- and condition-dependent drift, and $\eta_{t}$ is the observed innovation. The level represents accumulated condition, the increment represents local transition velocity, and their coupling distinguishes persistent degradation from fast operating transitions.

(6) $$z_{t+1}=A_{\psi }\left(z_{t},c_{t}\right)z_{t}+B_{\psi }\left(z_{t},c_{t}\right)\Delta z_{t}+b_{\psi }\left(z_{t},c_{t}\right)+\eta_{t}$$

Equation (7) selects the dynamic operator $f_{\psi }$ by combining one-step latent fidelity over *T* observations with a regularization term weighted by λ. $L_{\mathrm{dyn}}$ therefore rewards accurate transitions while constraining operator complexity and stability, providing the normal residual covariance later used by diffusion.

(7) $$L_{\mathrm{dyn}}=\frac{1}{T-1}\sum_{t=1}^{T-1}{∥z_{t+1}-f_{\psi }\left(z_{t},\Delta z_{t},c_{t}\right)∥}_{2}^{2}+\lambda {∥\psi ∥}_{2}^{2}$$

The health orientation is fixed after projection: the principal health coordinate is reversed when necessary so that larger values denote healthier states. Compressor health combines oil temperature, pressure-per-current efficiency, windowed control consistency, and latent health with weights of 0.25, 0.30, 0.15, and 0.30, respectively. PHM2024 health combines torque margin, measured gas temperature, and latent health with weights of 0.55, 0.25, and 0.20. Each component is mapped to [0,1] using normal-reference and adverse endpoints, and the resulting score *d* is converted to H=60+18d. Torque margin defines the PHM2024 reference-health readout and its evaluation; classification is supervised by the official fault indicator. The complete mappings and endpoint-estimation rules are given in Appendix A.

The representation is also conditioned by operating regime. Rather than using an unrestricted attention layer that may amplify accidental correlations, regime sensitivity enters through the canonical variables and the state-dependent transition. Graph-based few-shot prognostics [11], multivariate recurrent health modeling [12], and calibrated transformers under speed variation [69] each demonstrate that cross-channel coupling matters most when the operating regime changes. SDA2D makes that coupling explicit: the same latent level can evolve differently when its increment and context indicate a switching event, a thermal excursion, or a persistent efficiency loss.

**Figure 2:**
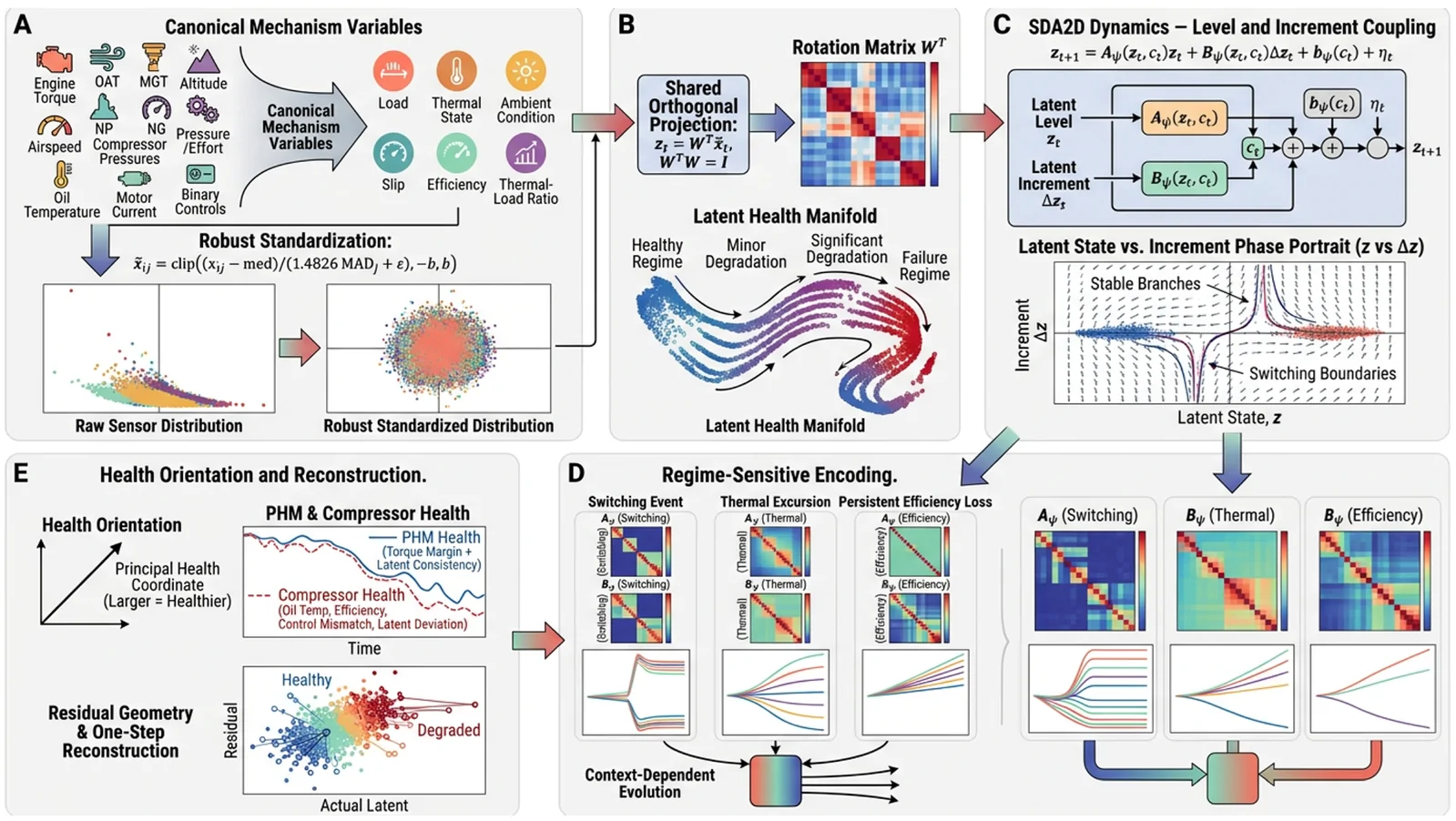
Unified health encoding and SDA2D dynamics. (**A**) Canonical mechanism variables and their asset-specific robust standardization; (**B**) orthogonal projection into the common health interface and the resulting latent health manifold; (**C**) coupling between latent state levels and increments in SDA2D, with the corresponding phase portrait; (**D**) regime-sensitive encoding and context-dependent evolution under switching events, thermal excursions, and persistent efficiency loss; (**E**) health-coordinate orientation, asset-specific health readouts, and residual geometry for one-step reconstruction.

Preserving operating regimes within a health representation is essential when rapid control responses coexist with slower condition changes. In Figure 2, asset-specific scaling and projection retain the channel relations through which these processes are observed, while level–increment coupling gives them distinct temporal roles. A local increment identifies motion relative to the current state; its interpretation depends on duty and environment. Similar health levels can consequently support different next-state responses during loading, switching, or recovery. The increasing-health orientation makes component readout consistent across assets, while separately fitted bases preserve their sensor geometry.

### 3.3. Conditional Latent Diffusion for Multi-Future Degradation

Equations (8) and (9) define the diffusion bridge. The forward distribution $q(z_{\tau }|z_{0})$ adds identity-covariance noise according to ${\bar{\alpha }}_{\tau }$, while the conditional reverse distribution $p_{\theta }\left(z_{\tau -1}|z_{\tau },c\right)$ is parameterized by mean $\mu_{\theta }$ and covariance $\Sigma_{\theta }$ at diffusion time τ. This paired construction converts a feasible future latent path into a noisy state and then reconstructs it under the complete condition c without replacing multimodality by a single mean trajectory.

(8) $$q\left(z_{\tau }|z_{0}\right)=N\;\left(\sqrt{{\bar{\alpha }}_{\tau }}z_{0},\left(1-{\bar{\alpha }}_{\tau }\right)I\right)$$

(9) $$p_{\theta }\left(z_{\tau -1}|z_{\tau },c\right)=N\;\left(\mu_{\theta }\left(z_{\tau },\tau ,c\right),\Sigma_{\theta }\left(z_{\tau },\tau ,c\right)\right)$$

Equation (10) trains the denoiser to recover the stochastic innovation ε from the noisy trajectory using $\varepsilon_{\theta }\left(z_{\tau },\tau ,c\right)$. The expectation spans diffusion time and innovation draws, so reverse corrections inherit the state-dependent residual geometry learned by SDA2D rather than an arbitrary isotropic uncertainty scale.

(10) $$L_{\mathrm{diff}}=E_{\tau ,\varepsilon }\;\left[{∥\varepsilon -\varepsilon_{\theta }\left(z_{\tau },\tau ,c\right)∥}_{2}^{2}\right]$$

Equation (11) samples B=500 candidate future latent trajectories from $p_{\theta }\left(Z|H_{t},c\right)$, all anchored to the same history and forecast horizon *H*. This ensemble is the single probabilistic source for failure probability, RUL, interval calibration, and tail risk; scenario changes act through c rather than through separate estimators.

(11) $${\left\{{\hat{z}}_{t+1:t+H}^{\left(k\right)}\right\}}_{k=1}^{B}∼p_{\theta }\left(Z|H_{t},c\right),\;B=500$$

The MetroPT3 history and candidate future have latent shapes 64×8 and 160×8, respectively. The diffusion-time index τ in Equations (8)–(10) indexes the noise and reverse-denoising sequence, whereas each physical forecast step spans two hours. The candidate count *B* is fixed before screening; the retained count $K=\sum_{k=1}^{B}G\left({\hat{Z}}^{\left(k\right)}\right)$ varies by origin, scenario, and seed. Predictive means and empirical quantiles use these *K* accepted paths.

Training uses $T_{\mathrm{diff}}=1000$ noise levels with $\beta_{\tau }=10^{-4}+\frac{\tau -1}{999}\left(0.02-10^{-4}\right)$ and ${\bar{\alpha }}_{\tau }=\prod_{s=1}^{\tau }\left(1-\beta_{s}\right)$. Forecast generation uses 100 DDIM updates [70] with zero added reverse-step variance. The sampler’s deterministic updates act on separately initialized candidate noise tensors, preserving an ensemble of 500 futures. The DDIM variance setting and the physical-transition covariance $\Sigma_{\eta }$ serve distinct roles.

The implementation selects zero-based noise-array indices $k_{i}=\mathrm{round}\left(1000i/100\right)=10i$, i=0,…,99, and traverses 990,980,…,0 during generation. Index *k* corresponds to the one-based noise level τ=k+1 in the variance schedule. The condition vector has 17 entries: present health (1), equipment structure (4), duty (3), environment (3), maintenance (2), and requested fault mode (4). The current eight-dimensional SDA2D state is projected to width 128 and added to the condition embedding.

At the final selected noise-array index, the sampler returns the predicted clean latent trajectory without an additional noise term. The MetroPT3 history encoder uses six Transformer layers with a model width of 512, eight attention heads, and a feedforward width of 2048. The noise predictor uses four Transformer layers with a model width of 128, eight attention heads, a feedforward width of 512, GELU activation, and a dropout rate of 0.1. Sinusoidal diffusion-time encoding is followed by an MLP; the condition embedding has widths 17→256→128. A linear 8→128 projection of the current SDA2D state supplies the history-conditioned state embedding. The SDA2D parameterization uses four-layer MLPs of hidden width 512 with inputs $\left(z_{t},c_{t}\right)$ to produce $A_{\psi }$, $B_{\psi }$, and $b_{\psi }$. The diagnosis head has widths 128→64→2, with ReLU and dropout 0.1.

Within MetroPT3, normal, early-fault, slow-leakage, sensor-drift, compound-fault, incomplete-recovery, high-stress, and missing-sensor experiments use the same encoder, base dynamics, and innovation covariance. Only the declared conditioning changes. That control is central to the counterfactual design: a difference between scenarios can be traced to a specified intervention instead of an undocumented change in preprocessing or model parameters.

The common innovation covariance $\Sigma_{\eta }$ is estimated from normal-validation one-step SDA2D residuals after 95% Mahalanobis-radius truncation. It remains fixed across the primary scenarios. Thermal-load conditioning acts through the state-dependent transition, while drift and missingness act through the observations; ensemble dispersion follows the conditional posterior without a scenario-specific covariance multiplier. The noise levels refer to externally imposed observation perturbations.

The variance schedule is chosen to preserve low-frequency degradation structure before high-frequency details are reconstructed. This ordering is consistent with probabilistic forecasting systems that separate global uncertainty from local residual variation [23,71] and with generative studies of correlated temporal noise [29]. The latent diffusion transformer literature [26] further supports the use of a compact latent trajectory rather than direct denoising of every raw channel. The schedule and covariance projection are held fixed across scenarios, ensuring that observed multimodality arises from the conditioning variables and learned trajectory distribution rather than scenario-specific noise scaling.

**Figure 3:**
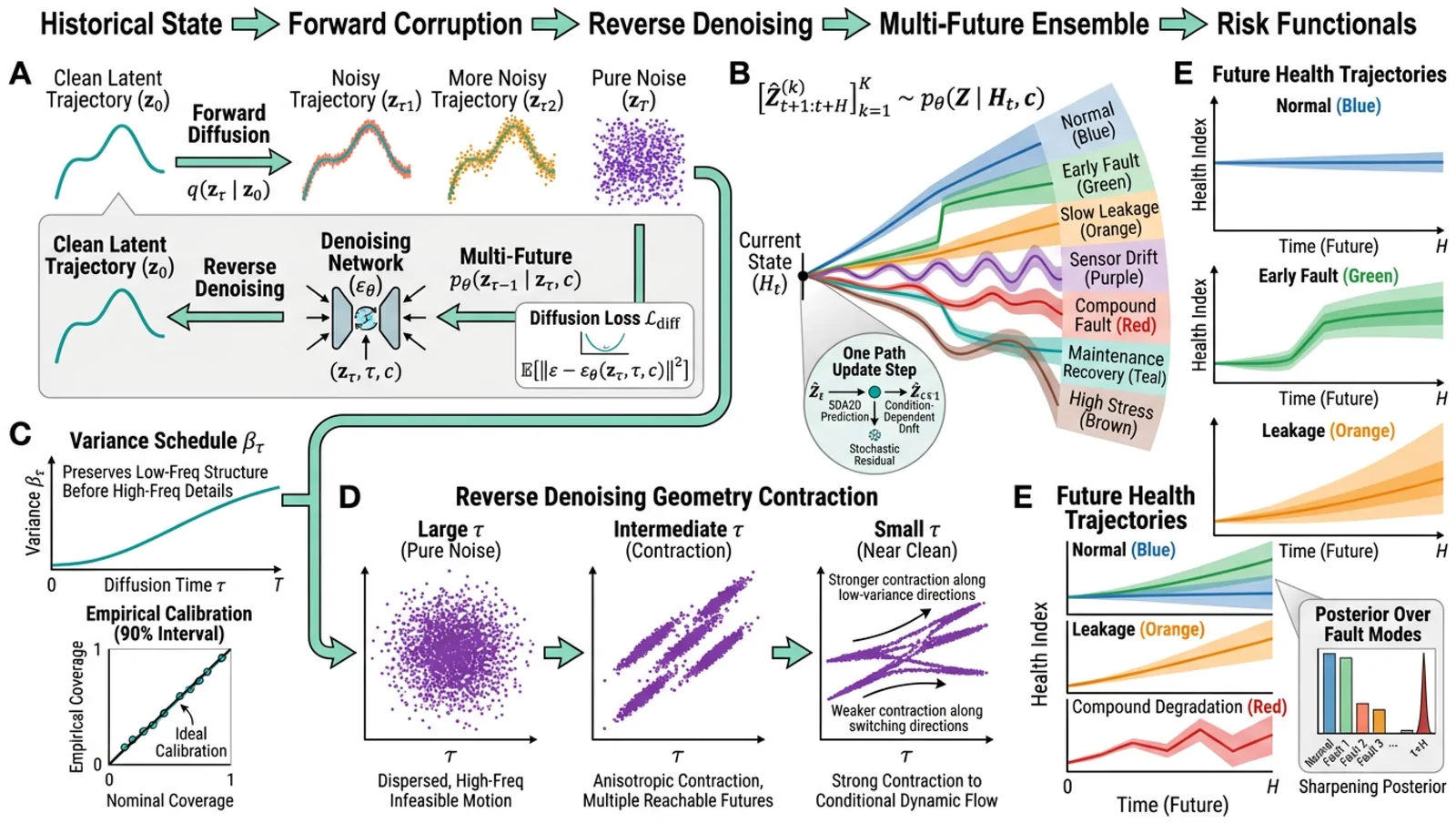
Conditional latent diffusion for multi-future degradation. (**A**) Forward corruption of clean latent trajectories and conditional reverse denoising with the diffusion training objective; (**B**) multi-future ensemble generation and a single-path update combining SDA2D prediction, condition-dependent drift, and stochastic residuals; (**C**) diffusion variance scheduling and empirical calibration of the nominal 90% prediction interval; (**D**) anisotropic contraction of latent trajectory geometry during reverse denoising toward conditionally feasible futures; (**E**) scenario-specific future health trajectories with uncertainty bands and posterior summaries of fault modes.

Generating a complete latent future preserves dependence between early departures, subsequent acceleration, and terminal health. Figure 3 connects this dependence to history-conditioned denoising: the condition vector enters the reverse process, and the SDA2D residual geometry constrains variation around the evolving state. Different candidate initializations produce alternative paths under the same intervention, retaining uncertainty in the timing and magnitude of deterioration. Their temporal dependence matters for first-passage statistics because successive threshold approaches belong to the same realization. The resulting ensemble supports comparisons of onset, curvature, and adverse severity within one conditional law.

### 3.4. Physics-Directed Rare-Fault Synthesis and Triple-Gate Semantic Validation

Equation (12) defines the fault direction $v_{f}$ as the unit-normalized difference between the fault and normal latent expectations. Unit normalization fixes the scale against which the displacement profile is specified. Equation (13) injects that direction into a normal trajectory $z_{\mathrm{normal}}\left(t\right)$ through a bounded temporal profile ρ(t;c,f) and residual $r_{\theta }\left(t\right)$, producing $z_{\mathrm{fault}}\left(t\right)$. Fault identity therefore controls a temporally structured departure while the local normal trajectory and stochastic continuity remain visible. The direction is used as a conditioning vector for scenario-specific trajectory generation and is then screened by the physical, dynamic, and semantic gates.

For benchmark construction, ρ(t;c,f) specifies the total displacement from the normal mean path at forecast time *t*. The reference is formed by superimposing this displacement on a completed deterministic normal rollout, while model-side stochastic residuals populate the conditional future ensemble.

(12) $$v_{f}=\frac{E(z|y=f)-E(z|y=\mathrm{normal})}{{∥E(z|y=f)-E(z|y=\mathrm{normal})∥}_{2}}$$

(13) $$z_{\mathrm{fault}}\left(t\right)=z_{\mathrm{normal}}\left(t\right)+\rho \left(t;c,f\right)v_{f}+r_{\theta }\left(t\right)$$

Equation (14) assembles the physical loss from signed residuals $g_{r}\left(\hat{Z}\right)$, weights $w_{r}$, and ReLU rectification. A residual is positive only in the infeasible region, so its squared rectification penalizes constraint violation while retaining admissible variability; $L_{\mathrm{phys}}$ aggregates the *R* asset-specific relations used by the validator.

(14) $$L_{\mathrm{phys}}=\sum_{r=1}^{R}w_{r}{\left(\mathrm{ReLU}(g_{r}\left(\hat{Z}\right))\right)}^{2}$$

Each generated trajectory is evaluated by three non-substitutable gates: asset-specific physical consistency, SDA2D residual consistency, and temporal-predicate semantics. “VLM4TS-inspired” refers specifically to the contextual verification of candidate time-series patterns [64]. The implemented semantic gate computes event descriptors from reconstructed channels and evaluates explicit timing and ordering predicates. The reported pipeline contains no vision–language backbone, image–text prompting, or VLM inference; its semantic decision is the predicate-violation count defined below.

For each gate *u*, $g_{u}$ denotes a violation measure, with larger values indicating poorer consistency. Acceptance requires $g_{u}\leq \kappa_{u}$, where the normal-validation threshold $\kappa_{u}$ is fixed across scenarios.

For MetroPT3, the physical score is the 95th percentile of the standardized valve–pressure residual along the forecast, and the dynamic score is the 95th percentile of the SDA2D Mahalanobis residual. The semantic score counts event-order violations. Their thresholds are $\kappa_{\mathrm{phys}}=3.0$, $\kappa_{\mathrm{dyn}}=2.5$, and $\kappa_{\mathrm{sem}}=0$, respectively. Calibration uses blocks 422–843 and freezes the thresholds before final evaluation; Equation (A12) specifies the trajectory-level aggregation.

The valve–pressure relation $f_{\mathrm{phys}}\left(TP2,TP3\right)$ is fitted by linear regression on normal fitting blocks 0–421, and $\sigma_{\mathrm{phys}}$ is the standard deviation of its fitting residuals. The semantic predicates require a controller transition to precede the corresponding pressure response within two forecast steps (4 h), pressure recovery to follow the order TP2→TP3→reservoir, and a requested escalation after a precursor to produce the corresponding latent displacement within ten steps (20 h). The semantic score sums violations of these temporal predicates.

Controller transitions use a change threshold of 0.5, pressure displacements use a detection threshold of 0.5 bar, and the pressure-recovery order permits a one-step timing tolerance. These event tolerances remain fixed across the scenario comparison.

(15) $$G\left(\hat{Z}\right)=\prod_{u\in \{\mathrm{phys},\mathrm{dyn},\mathrm{sem}\}}⊮\;\left(g_{u}\left(\hat{Z}\right)\leq \kappa_{u}\right)$$

The final gate is an intersection of physical, dynamic, and semantic acceptance criteria. A path enters the accepted ensemble only when it satisfies all three thresholds, preserving pressure logic, local SDA2D reachability, and the requested event order in the same future. This intersection makes world-model self-validation part of generation and keeps the evidence attached to a complete trajectory rather than to an isolated score.

Candidate acceptance uses the complete generated pool as its denominator. Each gate contributes a binary decision on the same candidate, and their intersection determines ensemble membership. Rejection attribution records the full set of failed gates, including simultaneous physical, dynamic, and semantic violations. This accounting distinguishes marginal gate acceptance, joint candidate retention, and validity within the retained ensemble; Appendix A.4 defines the corresponding counts and proportions.

Gate thresholds are estimated from normal validation residuals and then held fixed during scenario comparison. This avoids selecting a convenient threshold after viewing a rare-fault plot. Dual-level open-set augmentation [46] and imbalanced compressor generalization [49] motivate separate treatment of known variability and unknown faults, while self-supervised open-set learning [72] reduces dependence on rare labels. The physical residuals remain asset-specific, following the broader principle of mechanism-informed fault synthesis [60]. Consequently, a trajectory may be semantically consistent with leakage yet still fail because its valve sequence is impossible for the compressor under study.

**Figure 4:**
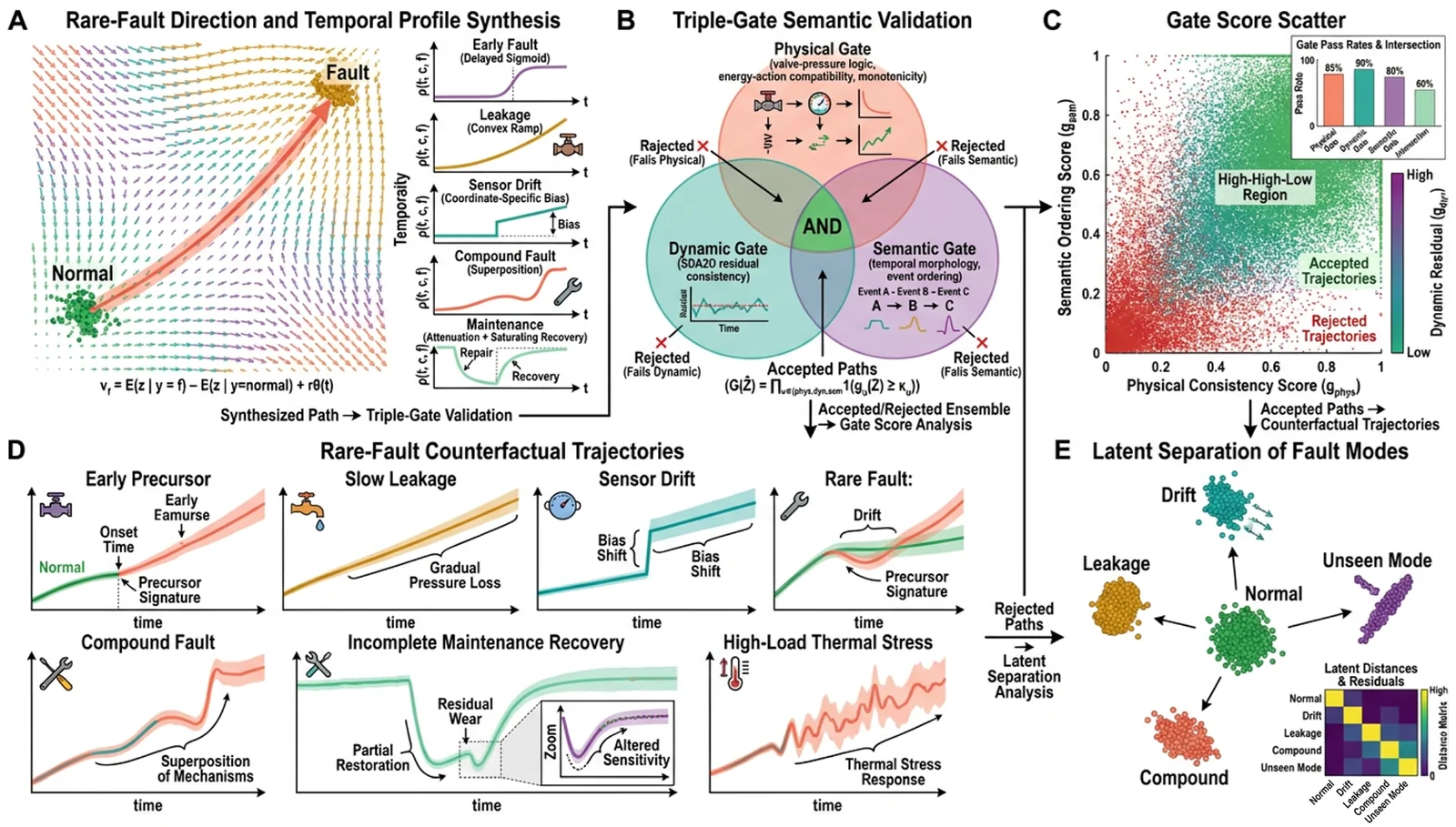
Physics -directed rare-fault synthesis and triple-gate semantic validation. (**A**) Fault-direction estimation and scenario-dependent temporal profile synthesis; (**B**) joint screening by physical, SDA2D dynamic, and temporal-predicate semantic gates; (**C**) gate-score scatter colored by dynamic residuals, with marginal and joint acceptance rates; (**D**) representative counterfactual trajectories under fault, sensor drift, maintenance, and thermal-load interventions; (**E**) latent separation of normal, drift, leakage, compound-fault, and unseen-mode representations, with the associated distance and residual matrix.

Mechanism displacement and residual variation play different roles in the construction in Figure 4. The fault direction fixes the orientation of the intervention, whereas its temporal coefficient controls the accumulated departure from normal evolution. Residual generation preserves variation around that mechanism-conditioned path. Physical relations constrain reconstructed channel combinations; the dynamic criterion assesses the consistency of successive latent states; temporal predicates evaluate the order of control and response events. Their intersection retains each requirement without allowing strong agreement with one criterion to compensate for another violation.

### 3.5. Risk Functionals and Training Procedure

Let *Q* denote the candidate path law $p_{\varphi }\left(dZ|H_{t},c\right)$ before screening, and let $A_{\kappa }=\left\{G_{\kappa }\left(Z\right)=1\right\}$ be acceptance under the threshold vector κ. The accepted law is

(16) $$Q_{\kappa }\left(dZ\right)=Q\left(dZ|A_{\kappa }\right)=\frac{G_{\kappa }\left(Z\right)\;Q\left(dZ\right)}{a_{\kappa }},\;a_{\kappa }=E_{Q}\left[G_{\kappa }\left(Z\right)\right]$$

All trajectory-derived probabilities, RUL distributions, VaR, and CVaR are conditional on $H_{t}$, *c*, and $A_{\kappa }$. For *B* generated paths, the empirical weights are $w_{k}=G_{\kappa }\left(Z_{k}\right)/\sum_{\ell =1}^{B}G_{\kappa }\left(Z_{\ell }\right)$. Every accepted path therefore carries mass 1/K. The reported estimators apply this normalization without additional importance or inverse-selection weights. The calibration objective in Equation (21) controls interval coverage and width while retaining these equal path weights.

Equations (16) and (17) evaluate first-passage risk under $Q_{\kappa }$. The failure boundary is $H_{\mathrm{crit}}=65$, whereas the warning boundary is $H_{\mathrm{warn}}=74$. Equation (16) counts first crossings anywhere within the next *h* steps, including paths that subsequently recover; substituting $H_{\mathrm{warn}}$ gives the warning probability. Equation (17) defines RUL by the first crossing of $H_{\mathrm{crit}}$. The RUL cumulative probability ${\hat{F}}_{\kappa }\left(h\right)$ at h≤H uses all *K* accepted paths as its denominator. Noncrossing paths contribute survival mass, with censoring proportion $1-{\hat{F}}_{\kappa }\left(160\right)$. A *q*-level RUL quantile is reached within the 320 h forecast when ${\hat{F}}_{\kappa }\left(160\right)\geq q$; otherwise it is recorded as exceeding 320 h.

(17) $$\hat{P}\left(T_{f}\leq t+h|H_{t},c,A_{\kappa }\right)=\frac{1}{K}\sum_{k=1}^{K}⊮\;\left(\min_{1\leq j\leq h}H^{\left(k\right)}\left(t+j\right)\leq H_{\mathrm{crit}}\right)$$

(18) $${\mathrm{RUL}}^{\left(k\right)}=\inf\left\{h>0:H^{\left(k\right)}\left(t+h\right)\leq H_{\mathrm{crit}}\right\}$$

At forecast step *h*, the health loss is $L_{h}=78-H\left(t+h\right)$, measured in health percentage points. The common health mapping gives $0\leq L_{h}\leq 18$. Equations (18) and (19) evaluate its upper-loss tail under the accepted law, with risk level γ=0.95. The subscript κ makes the gate dependence explicit: VaR locates the loss quantile, and CVaR evaluates the loss in the selected tail. Both use the accepted-path weights defined above.

(19) $${\mathrm{VaR}}_{\gamma ,\kappa }\left(L\right)=\inf\left\{l:P(L\leq l|H_{t},c,A_{\kappa })\geq \gamma \right\}$$

(20) $${\mathrm{CVaR}}_{\gamma ,\kappa }\left(L\right)=E\;\left(L|L\geq {\mathrm{VaR}}_{\gamma ,\kappa }\left(L\right),H_{t},c,A_{\kappa }\right)$$

For a failure or adverse-tail event *E*, the change in probability induced by screening satisfies

(19a) $$Q_{\kappa }\left(E\right)-Q\left(E\right)=\frac{{\mathrm{Cov}}_{Q}\left({⊮}_{E},G_{\kappa }\right)}{a_{\kappa }}.$$

Event mass increases when acceptance is positively associated with the event and decreases when that association is negative. Event-specific retention, $Q(A_{\kappa }|E)$, thus characterizes the selection of severe futures alongside the overall yield $a_{\kappa }$. VaR and CVaR respond to the corresponding change in the loss distribution. For two threshold vectors, the paired difference $\Delta_{\kappa }R=R\left({\hat{Q}}_{\kappa }\right)-R\left({\hat{Q}}_{\kappa_{0}}\right)$ evaluates a risk functional *R* on retained subsets of the same candidate pool. Holding the generated paths, health boundaries, and loss mapping fixed isolates the contribution of gate thresholds to probability and tail-risk estimates.

At each origin and evaluation step, the unfiltered candidates define the loss quantile $v_{0.95}^{\mathrm{all}}\left(h\right)$ and the fixed tail event $E_{0.95}\left(h\right)=\left\{L_{h}\geq v_{0.95}^{\mathrm{all}}\left(h\right)\right\}$. Tail retention is the number of accepted candidates in this event divided by its unfiltered count. The event definition remains fixed across gate settings, and the unfiltered pool has unit tail retention.

For a fixed origin, scenario, and seed, let $n_{E}=\sum_{k=1}^{B}{⊮}_{E}\left(Z_{k}\right)$ and $n_{E,\kappa }=\sum_{k=1}^{B}G_{\kappa }\left(Z_{k}\right){⊮}_{E}\left(Z_{k}\right)$. The empirical acceptance yield ${\hat{a}}_{\kappa }=K_{\kappa }/B$ and event-specific retention ${\hat{r}}_{\kappa }\left(E\right)$ give the exact relation

(19b) $$\begin{array}{cc} {\hat{r}}_{\kappa }\left(E\right) & =\frac{n_{E,\kappa }}{n_{E}},\\ {\hat{Q}}_{\kappa }\left(E\right) & =\hat{Q}\left(E\right)\frac{{\hat{r}}_{\kappa }\left(E\right)}{{\hat{a}}_{\kappa }}=\frac{n_{E,\kappa }}{K_{\kappa }},\\ {\hat{Q}}_{\kappa }\left(E\right)-\hat{Q}\left(E\right) & =\hat{Q}\left(E\right)\left[\frac{{\hat{r}}_{\kappa }\left(E\right)}{{\hat{a}}_{\kappa }}-1\right]. \end{array}$$

The retention ratio is defined for $n_{E}>0$, and the screened law for $K_{\kappa }>0$. Failure retention uses $E_{f}\left(h\right)=\left\{\min_{1\leq j\leq h}H\left(t+j\right)\leq 65\right\}$; adverse-tail retention uses the fixed unfiltered event $E_{0.95}\left(h\right)$. Preserving event frequency requires event retention to track overall acceptance. Its sensitivity is governed by their ratio, so similar retained counts can accompany different failure and tail probabilities.

Paired risk comparisons distinguish the effect of standard screening from the effect of a threshold perturbation. For *R* equal to horizon-specific failure probability, a first-passage RUL quantile, ${\mathrm{VaR}}_{0.95}$, or ${\mathrm{CVaR}}_{0.95}$, define

(19c) $$\begin{array}{cc} \Delta^{\mathrm{screen}}R & =R\left({\hat{Q}}_{\kappa_{0}}\right)-R\left(\hat{Q}\right),\\ \Delta_{\kappa }^{\mathrm{threshold}}R & =R\left({\hat{Q}}_{\kappa }\right)-R\left({\hat{Q}}_{\kappa_{0}}\right),\;\kappa_{0}=\left(3.0,2.5,0\right). \end{array}$$

The six populations comprise the unfiltered pool, the standard three-gate intersection, physical thresholds 2.7 and 3.3, and dynamic thresholds 2.25 and 2.75; each perturbation changes one threshold, with the remaining rules fixed. Each population is obtained by rescreening the same 500 candidate paths at an origin. Model weights, candidate initializations, generated health paths, loss mapping, and interval-calibration parameters remain fixed. Risk readout uses the generated health values; the affine interval-endpoint calibration does not transform the pathwise loss.

Failure probability counts first crossings by the stated horizon. RUL quantiles use the inverse empirical first-passage distribution on the two-hour grid without interpolation, including survival mass beyond 160 steps (320 h). A quantile whose cumulative event mass is below its level at step 160 is recorded as exceeding 320 h. VaR uses the inverse empirical loss distribution, and CVaR averages losses at or above that population’s VaR, retaining ties. In contrast, event-specific tail retention always uses the unfiltered tail threshold. These definitions preserve a common event for selection analysis while allowing each retained loss distribution to determine its own risk quantile.

**Figure 5:**
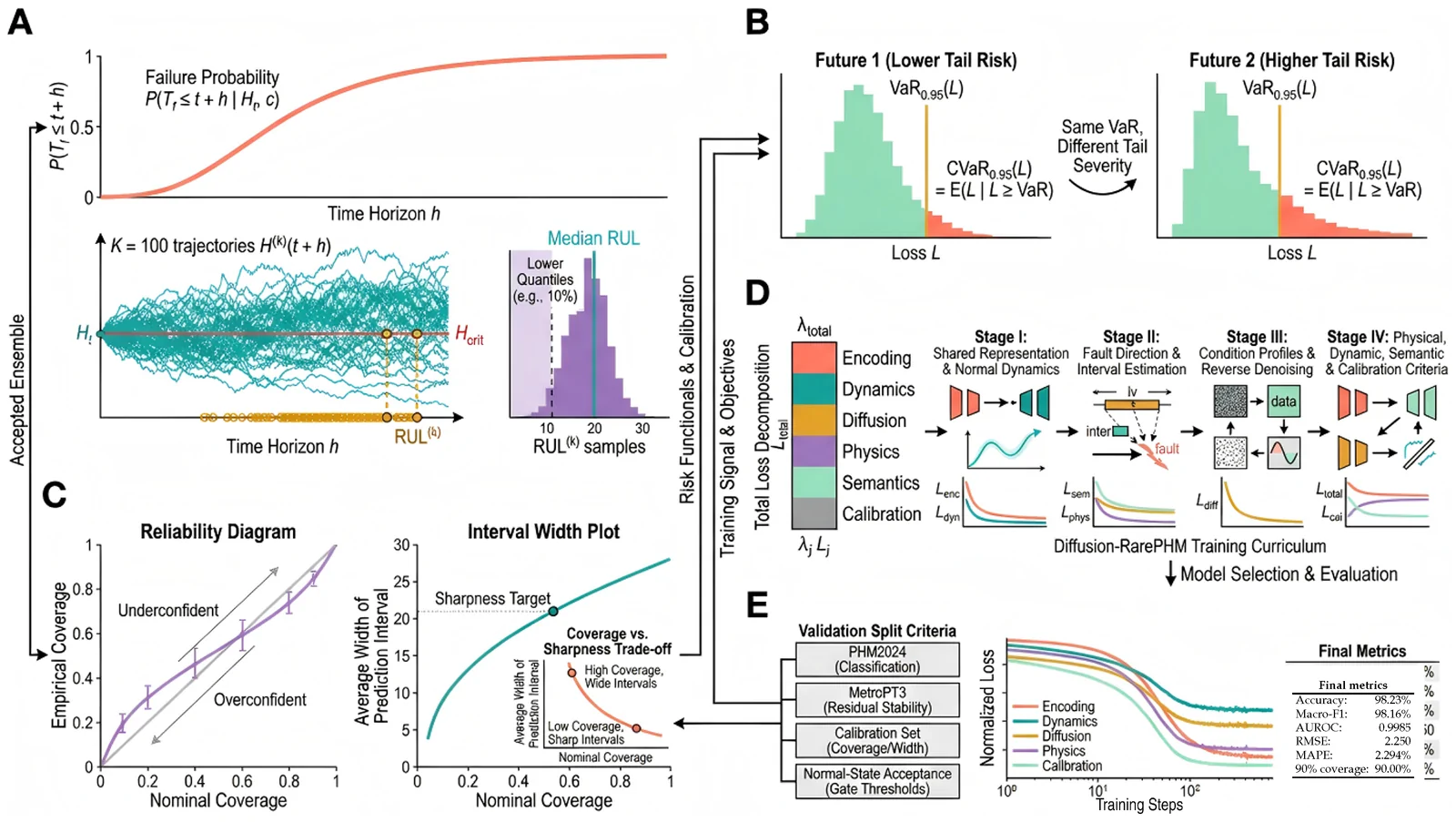
Risk functionals and training structure. Panels (**A**–**D**) schematically illustrate first-passage risk, tail severity, interval assessment, and the curriculum. Panel (**E**) lists the primary results. Trajectory probabilities are conditional on three-gate acceptance. Staged losses refer to MetroPT3; PHM2024 supplies the independently trained diagnostic readout.

Warning, RUL, and tail loss interrogate different features of the same accepted path law in Figure 5. Warning mass measures how widely an adverse threshold is reached, first-passage times locate that event within the horizon, and VaR/CVaR resolve the severity distribution at a specified future time. Scenarios with similar expected health can differ in all three respects when deterioration begins at different times or accumulates at different rates. Computing these quantities from one ensemble preserves their temporal and probabilistic relationships. Gate sensitivity then concerns the frequency of the retained events: rescreening a fixed candidate pool changes normalization and event-specific retention together, providing a direct measure of selection effects on the risk estimates.

Training uses separate optimizers for the PHM2024 and MetroPT3 parameter sets, $\Theta_{P}$ and $\Theta_{M}$. The PHM2024 classifier is fitted to official development records and fault labels by cross-entropy:

$$\begin{array}{cc} L_{P} & =-\frac{1}{N_{P}}\sum_{i=1}^{N_{P}}\log p_{\Theta_{P}}\left(y_{i}|x_{i}\right),\\ \Theta_{P}\cap \Theta_{M} & =⌀,\;\nabla_{\Theta_{P}}L_{M}=0,\;\nabla_{\Theta_{M}}L_{P}=0. \end{array}$$

Here, $p_{\Theta_{P}}$ is the single-record encoder–softmax classifier, and $L_{M}$ is the MetroPT3 criterion in Equation (20). PHM2024 optimization minibatches draw on official development records and fault labels; its synthetic training set is $D_{\mathrm{synth}}^{\left(P\right)}=⌀$. Fault synthesis and path selection act within the MetroPT3 branch. Both optimizers use AdamW with learning rate $2\times 10^{-4}$ and weight decay $10^{-4}$. The MetroPT3 criterion combines encoding, dynamics, diffusion, physics, semantic, and calibration terms. For calibration pair *i*, let $C_{\gamma }\left(x_{i}\right)=\left[\ell_{i},u_{i}\right]$ denote the interval derived from the accepted future ensemble. Optimization uses the product of two sigmoid boundary tests in Equation (21), where $\sigma \left(v\right)={\left(1+e^{-v}\right)}^{-1}$. The temperature ϑ=1.0 acts on differences in health percentage points. Hard interval membership defines empirical coverage at evaluation. The training criterion balances soft coverage discrepancy and width over *N* calibration origin–horizon pairs.

(21) $$L_{\mathrm{total}}=\sum_{j\in \{\mathrm{enc},\mathrm{dyn},\mathrm{diff},\mathrm{phys},\mathrm{sem},\mathrm{cal}\}}\lambda_{j}L_{j}$$

(22) $$\begin{array}{cc} {\tilde{m}}_{\vartheta }\left(y_{i};\ell_{i},u_{i}\right) & =\sigma \;\left(\frac{y_{i}-\ell_{i}}{\vartheta }\right)\sigma \;\left(\frac{u_{i}-y_{i}}{\vartheta }\right),\\ {\tilde{C}}_{\gamma } & =\frac{1}{N}\sum_{i=1}^{N}{\tilde{m}}_{\vartheta }\left(y_{i};\ell_{i},u_{i}\right),\\ L_{\mathrm{cal}} & =\left|{\tilde{C}}_{\gamma }-\gamma \right|+\frac{\beta }{N}\sum_{i=1}^{N}\left(u_{i}-\ell_{i}\right). \end{array}$$

Let $s_{i}^{\ell }=\sigma \left(\left(y_{i}-\ell_{i}\right)/\vartheta \right)$, $s_{i}^{u}=\sigma \left(\left(u_{i}-y_{i}\right)/\vartheta \right)$, and ${\tilde{m}}_{i}=s_{i}^{\ell }s_{i}^{u}$. Writing $\xi =\mathrm{sgn}({\tilde{C}}_{\gamma }-\gamma )$ away from zero coverage discrepancy gives

$$\begin{array}{cc} \frac{\partial L_{\mathrm{cal}}}{\partial \ell_{i}} & =-\frac{\xi }{N\vartheta }{\tilde{m}}_{i}\left(1-s_{i}^{\ell }\right)-\frac{\beta }{N},\\ \frac{\partial L_{\mathrm{cal}}}{\partial u_{i}} & =\frac{\xi }{N\vartheta }{\tilde{m}}_{i}\left(1-s_{i}^{u}\right)+\frac{\beta }{N}. \end{array}$$

The absolute discrepancy uses a subgradient at zero. For seed *s*, $a_{s}=\mathrm{softplus}\left(\alpha_{s}\right)+10^{-6}$, $\ell_{i}=a_{s}q_{0.05,i}+b_{s}$, and $u_{i}=a_{s}q_{0.95,i}+b_{s}$. Here, $\mathrm{softplus}\left(v\right)=\log\left(1+e^{v}\right)$, so positive scale preserves endpoint order and $\partial a_{s}/\partial \alpha_{s}=\sigma \left(\alpha_{s}\right)$. The training quantile estimator applies a Gaussian kernel to the ranks of sorted accepted-path health values. For calibration pair *i*, let ${\hat{H}}_{i,(1)}\leq \cdots \leq {\hat{H}}_{i,(K_{i})}$ denote these values. With $K_{i}\geq 2$,

$$\begin{array}{cc} r_{i,k} & =\frac{k-1}{K_{i}-1},\;h_{q}=0.02,\\ \omega_{i,k}\left(p\right) & =\frac{\exp\;\left[-{\left(r_{i,k}-p\right)}^{2}/\left(2h_{q}^{2}\right)\right]}{\sum_{v=1}^{K_{i}}\exp\;\left[-{\left(r_{i,v}-p\right)}^{2}/\left(2h_{q}^{2}\right)\right]},\\ {\tilde{q}}_{p,i} & =\sum_{k=1}^{K_{i}}\omega_{i,k}\left(p\right){\hat{H}}_{i,(k)},\;p\in \left\{0.05,0.95\right\}. \end{array}$$

The fixed bandwidth $h_{q}$ is expressed in quantile-level units. A singleton accepted set returns its sole health value. Training uses $q_{p,i}={\tilde{q}}_{p,i}$. With *p*, $h_{q}$, and $K_{i}$ fixed, rank weights are independent of sample magnitudes and $\partial {\tilde{q}}_{p,i}/\partial {\hat{H}}_{i,(k)}=\omega_{i,k}\left(p\right)$. Sorting routes piecewise gradients to the corresponding generated values; quantile computation uses no straight-through operation. Evaluation uses the discrete empirical inverse distribution, $q_{p,i}^{\mathrm{emp}}={\hat{H}}_{i,(\left\lceil pK_{i}\right\rceil )}$, on the same health scale. Generated health values and quantile estimates remain connected to the reverse sampling graph; Stage IV retains gradients through this computation. The denoiser contribution follows the chain rule:

$$\nabla_{\theta }L_{\mathrm{cal}}=a_{s}\sum_{i}\left[\frac{\partial L_{\mathrm{cal}}}{\partial \ell_{i}}\nabla_{\theta }q_{0.05,i}+\frac{\partial L_{\mathrm{cal}}}{\partial u_{i}}\nabla_{\theta }q_{0.95,i}\right].$$

MetroPT3 Stage IV updates the denoiser and $\left(\alpha_{s},b_{s}\right)$ with the encoder, SDA2D, and fault direction frozen. Calibration gradients reach the denoiser through the generated health distribution and its quantile estimates. The scale and shift act on interval endpoints; the point forecast remains the mean of the accepted health paths. Thus, generator updates affect point predictions, while endpoint calibration adjusts interval location and width. The calibration ablation retrains the MetroPT3 trajectory branch without $L_{\mathrm{cal}}$ and evaluates raw accepted-path intervals. Its diagnosis reference is the independent PHM2024 classifier. Final coverage in Table 3 uses empirical accepted-set quantiles, frozen $\left(a_{s},b_{s}\right)$, and the hard reference-inclusion indicator. Calibrated endpoints remain untruncated; generated health values retain the [60,78] mapping.

Paired endpoint diagnostics use a fixed final generator, the same accepted paths, and the same *N* calibration cases for each seed. Kernel and empirical readouts share the same frozen interval parameters $\left(a_{s},b_{s}\right)$ within each seed. For a specified quantile readout, raw and calibrated coverage and width are

$$\begin{array}{cc} C_{s}^{\mathrm{hard},\mathrm{raw}} & =\frac{1}{N}\sum_{i=1}^{N}1\;\left[q_{0.05,i}\leq y_{i}\leq q_{0.95,i}\right],\\ C_{s}^{\mathrm{hard},\mathrm{cal}} & =\frac{1}{N}\sum_{i=1}^{N}1\;\left[\ell_{i}\leq y_{i}\leq u_{i}\right],\\ {\bar{W}}_{s}^{\mathrm{raw}} & =\frac{1}{N}\sum_{i=1}^{N}\left(q_{0.95,i}-q_{0.05,i}\right),\\ {\bar{W}}_{s}^{\mathrm{cal}} & =\frac{1}{N}\sum_{i=1}^{N}\left(u_{i}-\ell_{i}\right)=a_{s}{\bar{W}}_{s}^{\mathrm{raw}}. \end{array}$$

Coverage here is a proportion. Soft coverage applies Equation (21) to the corresponding raw or calibrated endpoints on these same cases. The shift $b_{s}$ changes the interval location and cancels from its width. This paired comparison isolates the endpoint transformation within the final model; the calibration-removal ablation assesses a separately retrained configuration.

The membership temperature ϑ acts in health percentage points. For $w_{i}=u_{i}-\ell_{i}\geq 0$, the sigmoid product is maximized when the reference lies at the interval midpoint, giving the pointwise and batch bounds

$$\begin{array}{cc} {\tilde{m}}_{\vartheta }\left(y_{i};\ell_{i},u_{i}\right) & \leq \sigma \;{\left(\frac{w_{i}}{2\vartheta }\right)}^{2},\\ {\tilde{C}}_{\gamma } & \leq U_{s,\vartheta }=\frac{1}{N}\sum_{i=1}^{N}\sigma \;{\left(\frac{w_{i}}{2\vartheta }\right)}^{2}. \end{array}$$

For an individual 2.05-point interval at ϑ=1.0, maximum soft membership is 0.542 at the midpoint and 0.443 at either endpoint, where hard membership remains one. The surrogate therefore measures smooth boundary proximity. Its discrepancy from γ=0.90 is an optimization penalty, and hard inclusion supplies the reported coverage. The derivative bound $d\{\sigma {\left(w/2\vartheta \right)}^{2}\}/dw\leq 4/\left(27\vartheta \right)$ also yields

$${\tilde{C}}_{\gamma }\leq \frac{1}{4}+\frac{4\bar{W}}{27\vartheta },\;\bar{W}=\frac{1}{N}\sum_{i=1}^{N}w_{i}.$$

For any width distribution with mean 2.05 points and ϑ=1.0, this bound is 0.554. Thus, 90% hard inclusion and a soft score near 0.54 refer to different quantities. The accompanying reference implementation evaluates kernel and empirical readouts, hard and soft membership, width, and its distribution-dependent soft bound using fixed seed-specific parameters.

The MetroPT3 curriculum activates encoding and dynamics in Stage I, fault-direction estimation through Equation (12) in Stage II, diffusion, physical, and semantic terms in Stage III, and physical, semantic, and calibration terms in Stage IV. The semantic term counts event-order violations. PHM2024 classification follows its independent cross-entropy objective. At the gate-score level, the straight-through operation combines hard passage with a sigmoid relaxation:

$$\begin{array}{cc} b_{u} & =1\left[g_{u}\leq \kappa_{u}\right],\;p_{u}=\sigma \;\left(\frac{\kappa_{u}-g_{u}}{\tau_{u}}\right),\\ b_{u}^{\mathrm{ST}} & =b_{u}+p_{u}-\mathrm{sg}\left(p_{u}\right),\;\tau_{u}=1.0,\\ {\left.\frac{\partial b_{u}^{\mathrm{ST}}}{\partial g_{u}}\right|}_{\mathrm{backward}} & =-\frac{p_{u}\left(1-p_{u}\right)}{\tau_{u}}. \end{array}$$

Here, sg preserves the forward value and stops its gradient. The straight-through derivative is defined with respect to the gate score. For differentiable physical and dynamic scores, it composes with the score’s trajectory derivative. A semantic score computed from integer first-crossing indices has zero derivative through those hard indices. Its count enforces event order in forward validation and enters the reported semantic objective; hard index extraction is not assigned a continuous trajectory gradient. Sorting and kernel quantiles route gradients through the values retained by the forward decisions. Gate temperature $\tau_{u}$ acts on its score scale, whereas membership temperature ϑ acts in health percentage points. Normal calibration cases determine the final hard thresholds (3.0,2.5,0) before evaluation.

**Figure 6:**
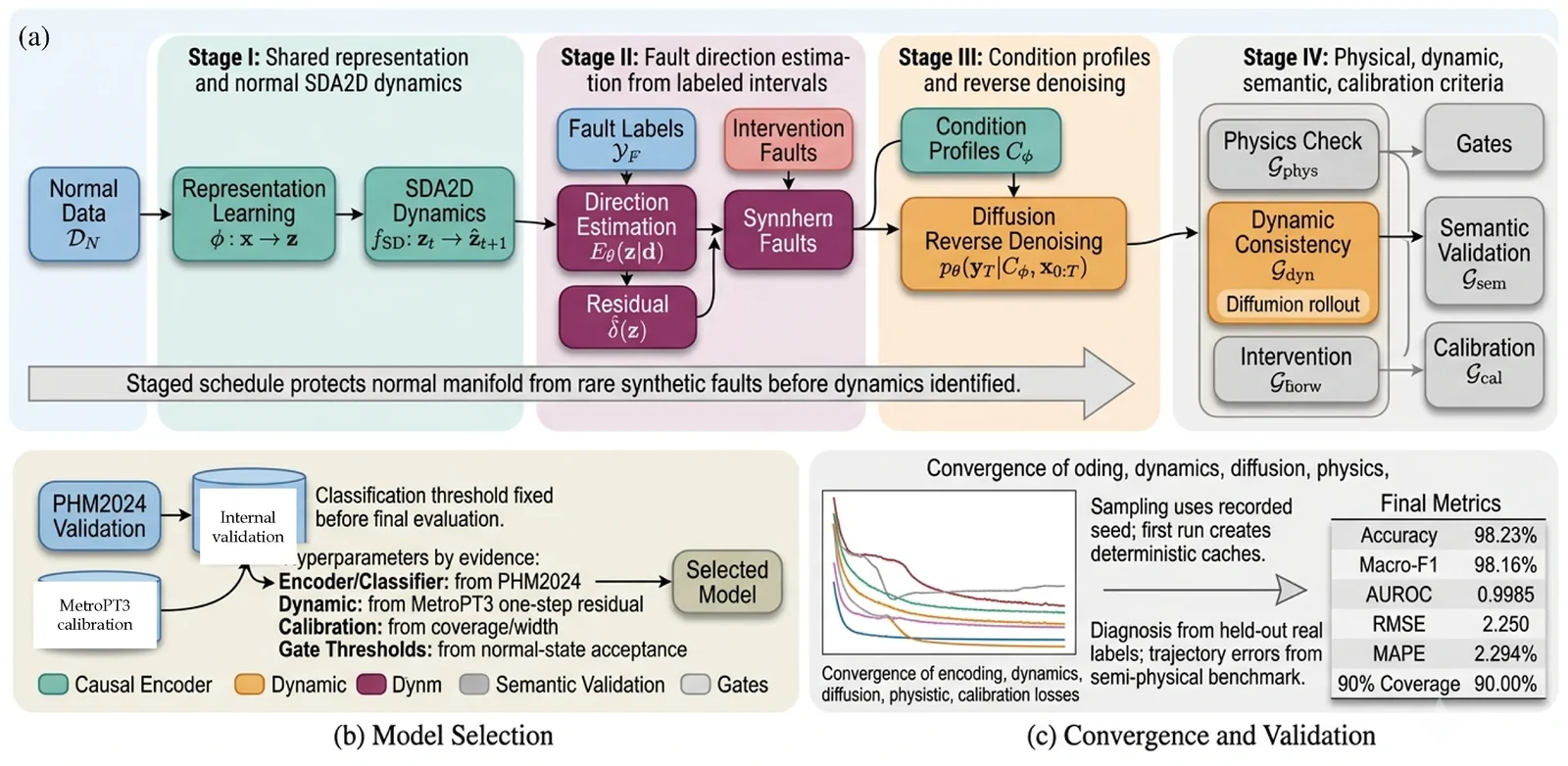
Four-stage training curriculum, model selection, and convergence validation. Asset-specific representation fitting within the common health interface is followed by fault-direction estimation, conditional reverse generation, multi-gate validation, and evidence-specific hyperparameter selection. The trajectory curriculum refers to MetroPT3; PHM2024 diagnosis uses independent cross-entropy optimization. Model selection uses PHM2024 development validation and MetroPT3 calibration blocks. Held-out scores enter final evaluation.

Fixing normal transition geometry before introducing fault-directed generation gives the curriculum in Figure 6 a clear statistical structure. Representation fitting establishes the sensor-to-state relation, and direction estimation identifies the departure used for intervention. Conditional diffusion then models variation around this reference geometry. During Stage IV, denoiser updates and interval calibration act on generated distributions while the encoder and SDA2D fit remain fixed, preserving the physical interpretation of latent motion. The designated development and calibration partitions determine model selection before final evaluation.

Each PHM2024 protocol fits medians, MAD scales, projection, and reference-health endpoints within development; classifier hyperparameters use its internal model-selection records. The record-level partition comprises 112,500 development and 37,500 held-out observations. Operating-condition-held-out models use their separately defined development partition. Both protocols independently retrain diagnosis at seeds 2026–2030 and score held-out labels with frozen transforms and classifiers.

For the record-level full model, the independent PHM2024 Stage I fit jointly optimizes its health encoder and diagnosis head for 50 epochs. The diagnostic readout accompanying the deterministic MetroPT3 configuration instead freezes the PHM2024 encoder at its Stage I checkpoint and trains a newly initialized diagnosis head for 50 epochs. Appendix B reports the seed-level macro-F1 scores for these two diagnostic protocols.

MetroPT3 normal representation, SDA2D, and deterministic reference construction use one seed-2026 fit on blocks 0–421, shared across the five realizations. Conditional diffusion is independently retrained at seeds 2026–2030 with common data and distinct initialization and sampling streams. Each realization fits its global interval scale and shift on the same 128 normal calibration origins from blocks 422–843: N=128×160=20,480 origin–horizon pairs, nominal coverage 0.90, and width coefficient β=0.1. Equation (21) supplies the differentiable objective; calibration selection evaluates hard coverage and width on these cases. Models, mappings, gate thresholds, and interval parameters are frozen after Stage IV. Final evaluation origins and their futures lie in blocks 844–2106.

The trajectory protocol uses 256 origins and 500 candidates per origin and seed: 128,000 proposals per scenario and seed, or 640,000 over five seeds. Within each seed, scenario metrics pool origins and forecast steps; the five seed values are averaged, followed by equal weighting of the nine scenarios. Errors and coverage use the common semi-physical reference. Accepted-set admissibility counts valid paths within the retained population, whereas candidate yield uses all proposals as its denominator.

Parameter selection follows the statistical role of each component. Encoder and classifier settings use PHM2024 development evidence; dynamic regularization uses MetroPT3 one-step residual stability; interval parameters balance coverage and width on the designated calibration cases; and gate thresholds use normal-state acceptance. This separation preserves the different scales on which state discrimination, transition consistency, and interval concentration are assessed [5,7,22,73]. Section 4.5 examines how gate perturbation and calibration removal affect their corresponding evaluation quantities.

## 4. Results and Validation

Diagnosis and future propagation impose complementary demands on the health state. Fault-label discrimination tests whether operating variation and condition evidence remain distinguishable; transition fidelity tests whether those relations persist during evolution. Complete future paths additionally preserve dependence between onset, acceleration, and eventual severity. The following evidence relates these properties to prediction error, retained-ensemble composition, and interval and risk statistics within the respective asset tasks. In the trajectory panels, curves labeled “Observed” denote the constructed benchmark health reference $H^{\mathrm{ref}}$; measured sensor chronologies provide the historical evidence used for initialization and transition fitting.

Joint screening retains 5,398,400 of the 5,760,000 candidates generated across nine scenarios and five seeds. Scenario acceptance ranges from 91.5% for compound faults to 97.0% for normal operation, with a nine-scenario mean of 93.72%. Each origin proposes 500 paths; scenario-mean retained sizes are 457.5–485.0, the minimum is 440, and no origin has an empty accepted set. Within-origin health dispersion remains 2.59–2.77 percentage points after screening, compared with 3.06–3.21 before screening. Table A4, Table A5 and Table A6 report the joint gate counts, marginal passage, exclusive rejection patterns, and retained-ensemble summaries. The 100.00% accepted-set admissibility records satisfaction of the three defining criteria within this retained population.

### 4.1. Common Health Interface and Asset-Specific Dynamic Fidelity

**Figure 7:**
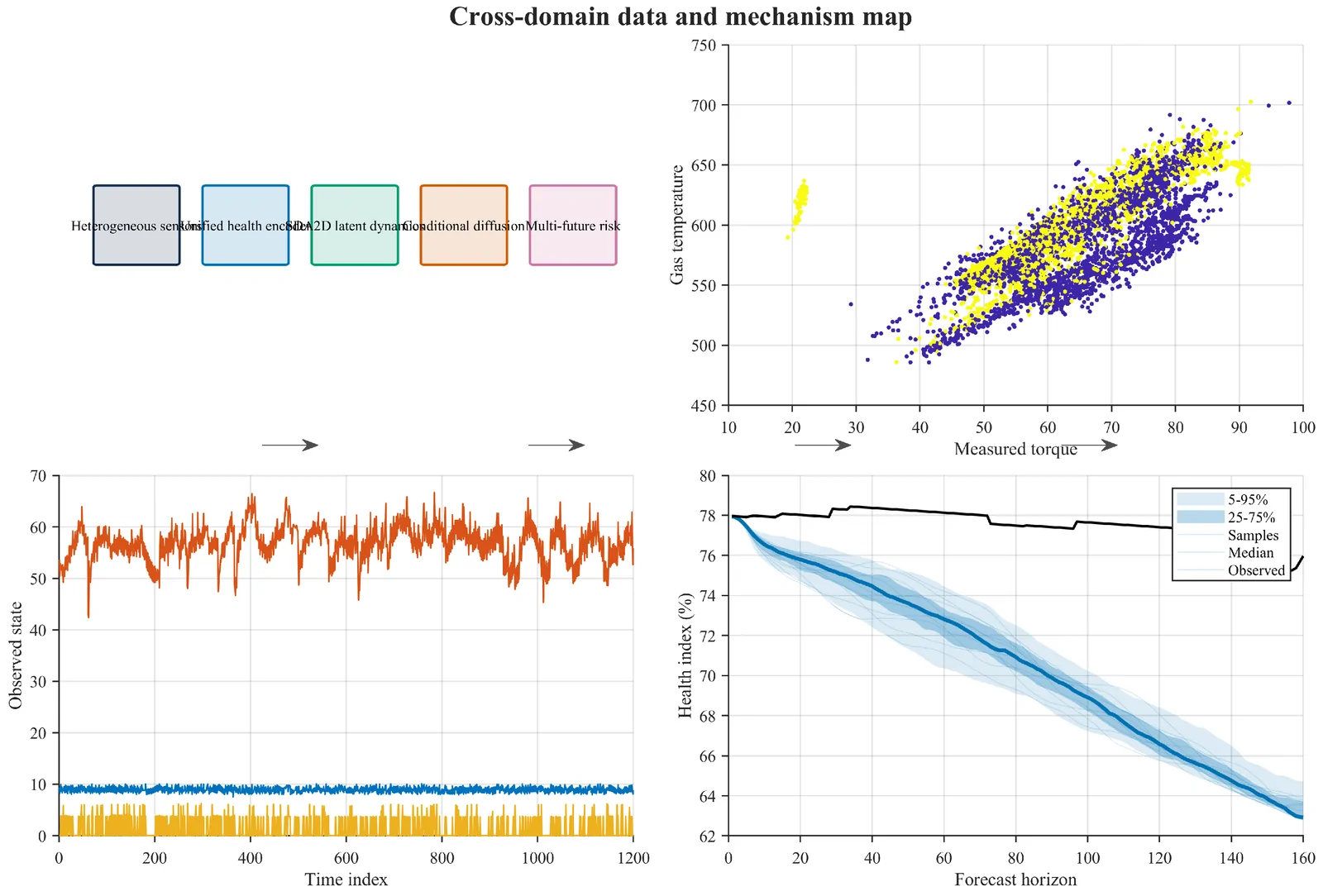
Asset-native evidence and mechanism map linking engine and compressor observations through the common health interface to their task-specific outputs. In the upper-right PHM2024 scatter panel, yellow denotes normal observations (label 0) and purple denotes faulty observations (label 1).

Figure 7 maps engine torque–thermal–speed relationships and compressor pressure–current–control relationships into separately fitted eight-dimensional states. PHM2024 evaluates measured-label discrimination; MetroPT3 supplies chronological transition fitting and controlled future generation. The common state format preserves these asset-specific sensing and supervision roles.

**Figure 8:**
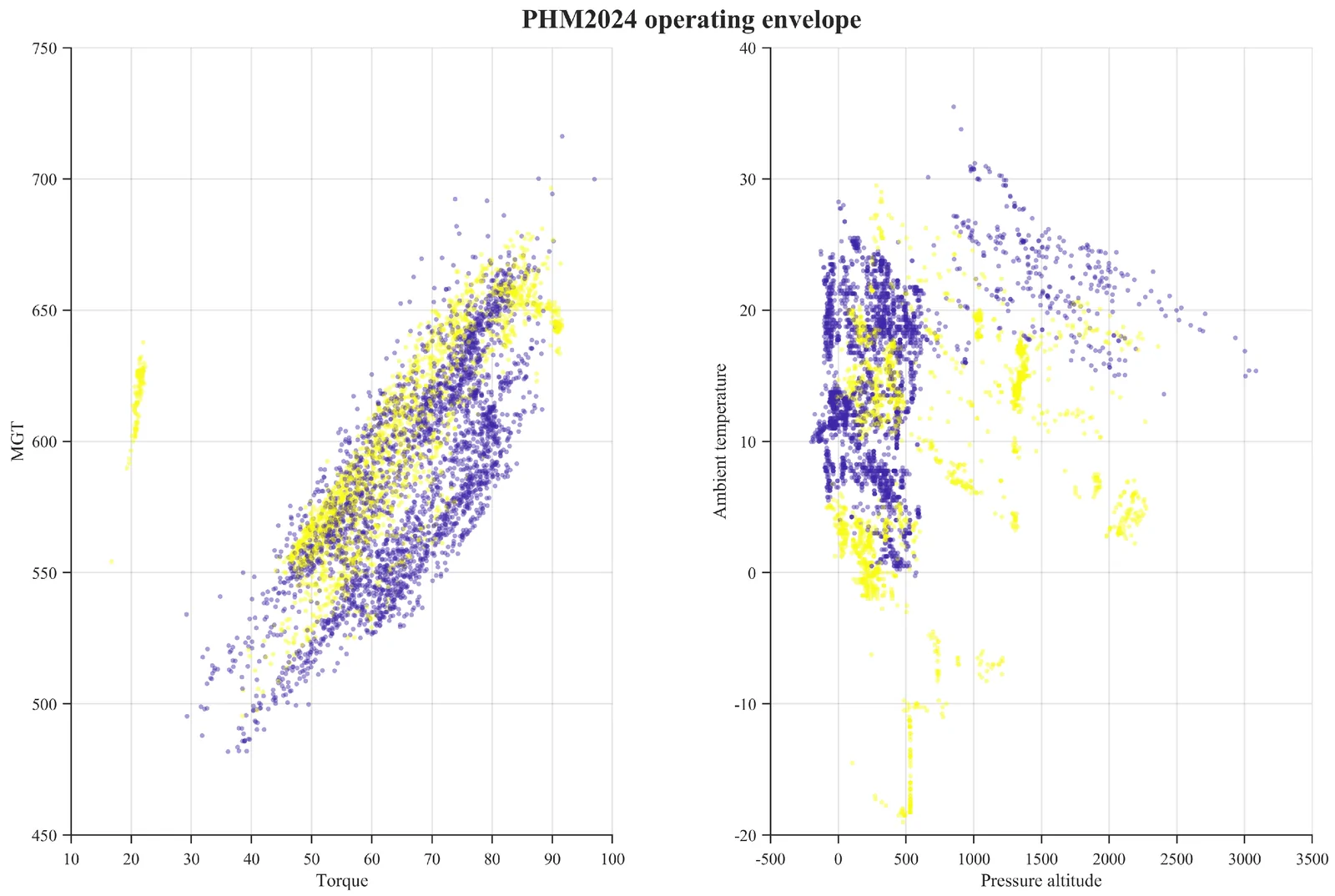
PHM2024 operating envelope showing condition-dependent overlap and multivariate separation of normal and faulty observations. In both scatter panels, yellow denotes normal observations (label 0) and purple denotes faulty observations (label 1).

The overlapping operating envelopes in Figure 8 make diagnosis depend on conditional sensor relationships. Accuracy is 98.23% under the record-level protocol and 97.86% under operating-condition separation, a 0.37-percentage-point difference. The held-out condition groups change the ambient and rotational configurations available during training.

**Figure 9:**
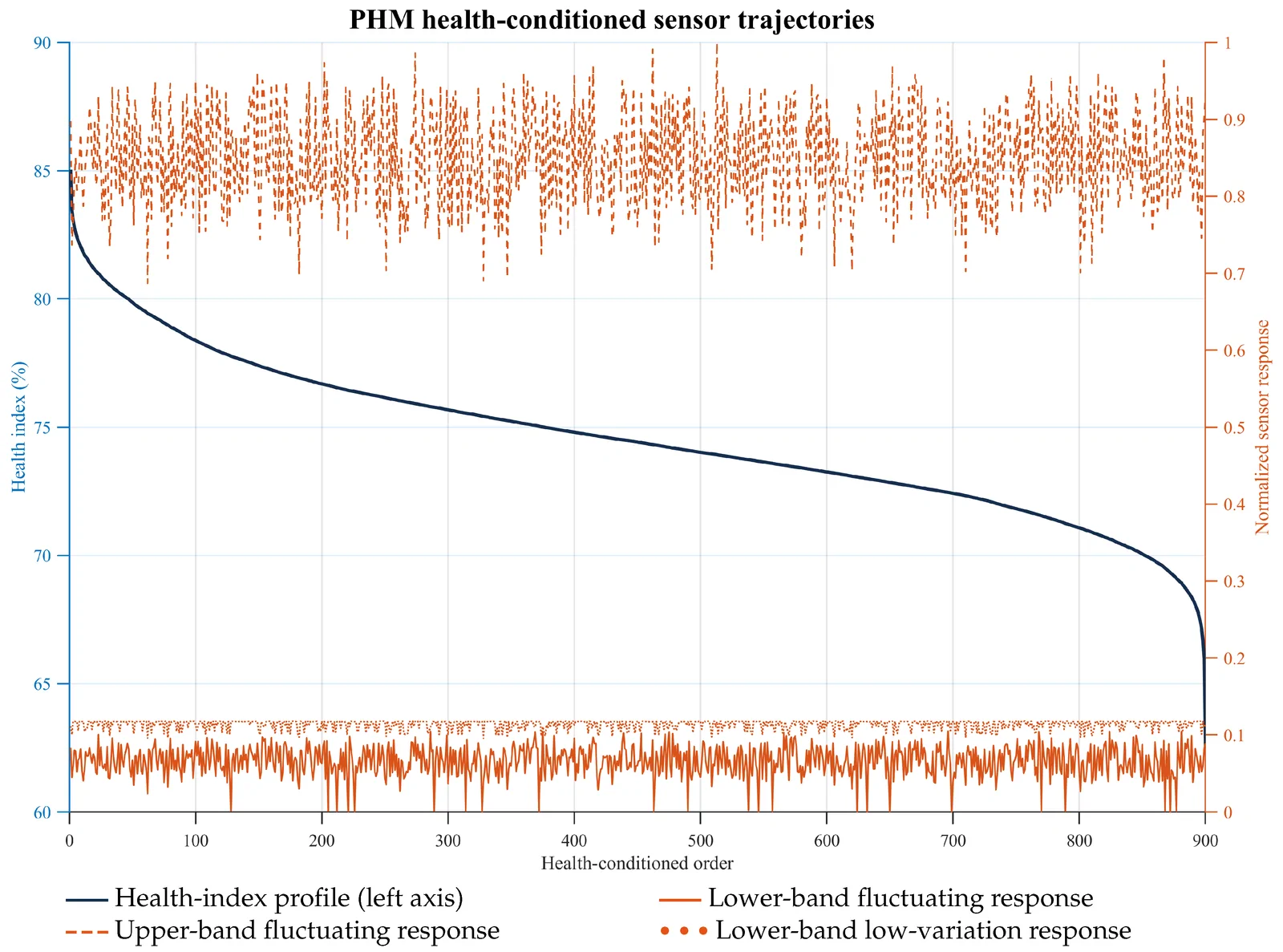
Health-conditioned PHM2024 profiles. The health curve uses the left axis and the three normalized sensor responses use the right axis. The horizontal coordinate orders observations by health rather than acquisition time.

Figure 9 separates broad sensor-response variation from the ordered health reference. Torque margin and thermal evidence define that reference, while measured operating and shaft-speed variables enter diagnosis. The official fault indicator supervises classification; chronological transition learning uses the compressor histories.

**Figure 10:**
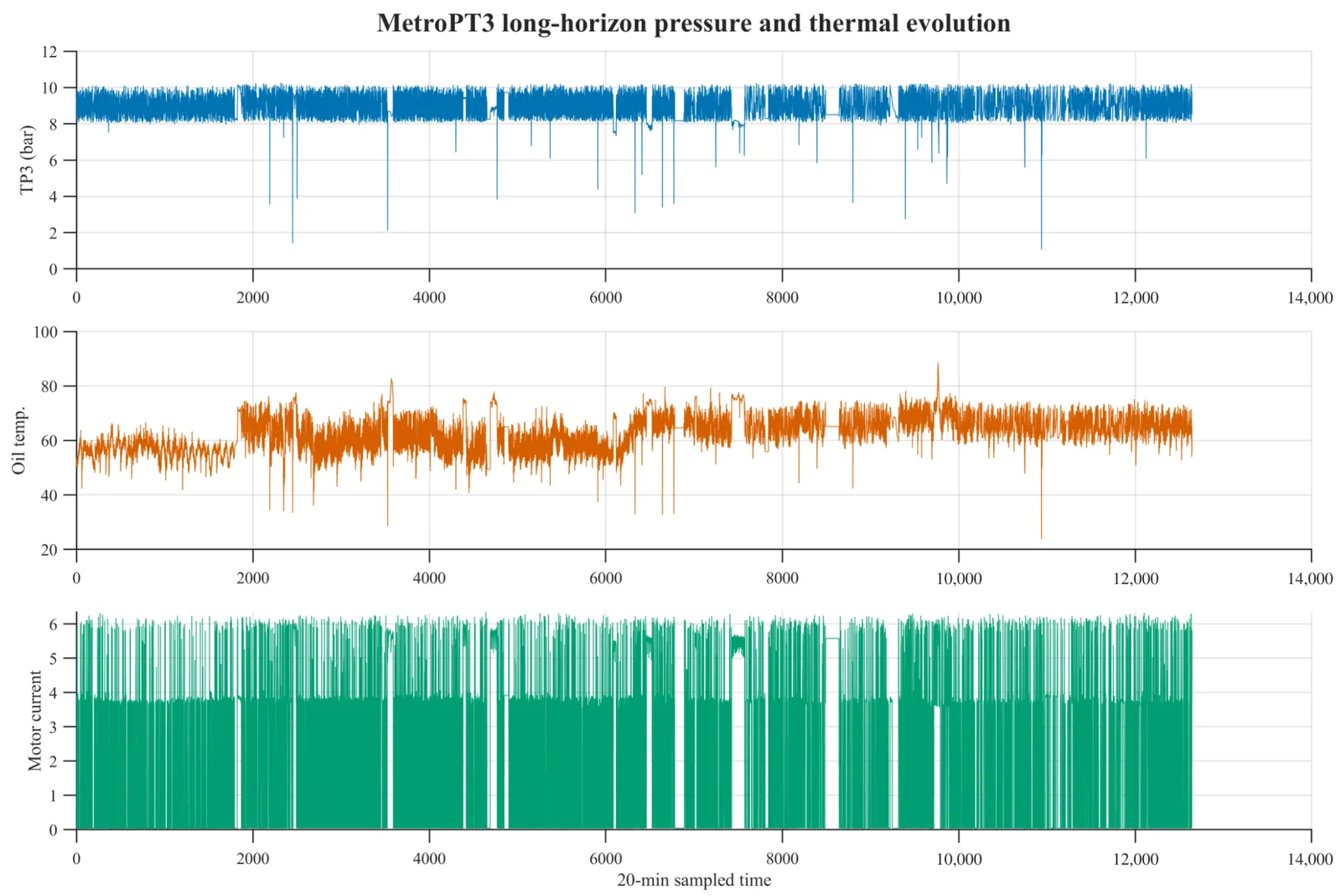
Seven-month MetroPT3 pressure, thermal, and electrical chronology-preserving regulation cycles, slow drift, and operational discontinuities.

Pressure regulation, thermal variation, and electrical loading coexist in the MetroPT3 chronology (Figure 10). Twenty-minute retention preserves these operating responses; six-sample aggregation defines the two-hour forecast grid. Level–increment fitting separates local reversible motion from sustained state displacement under the common normal-transition reference.

**Figure 11:**
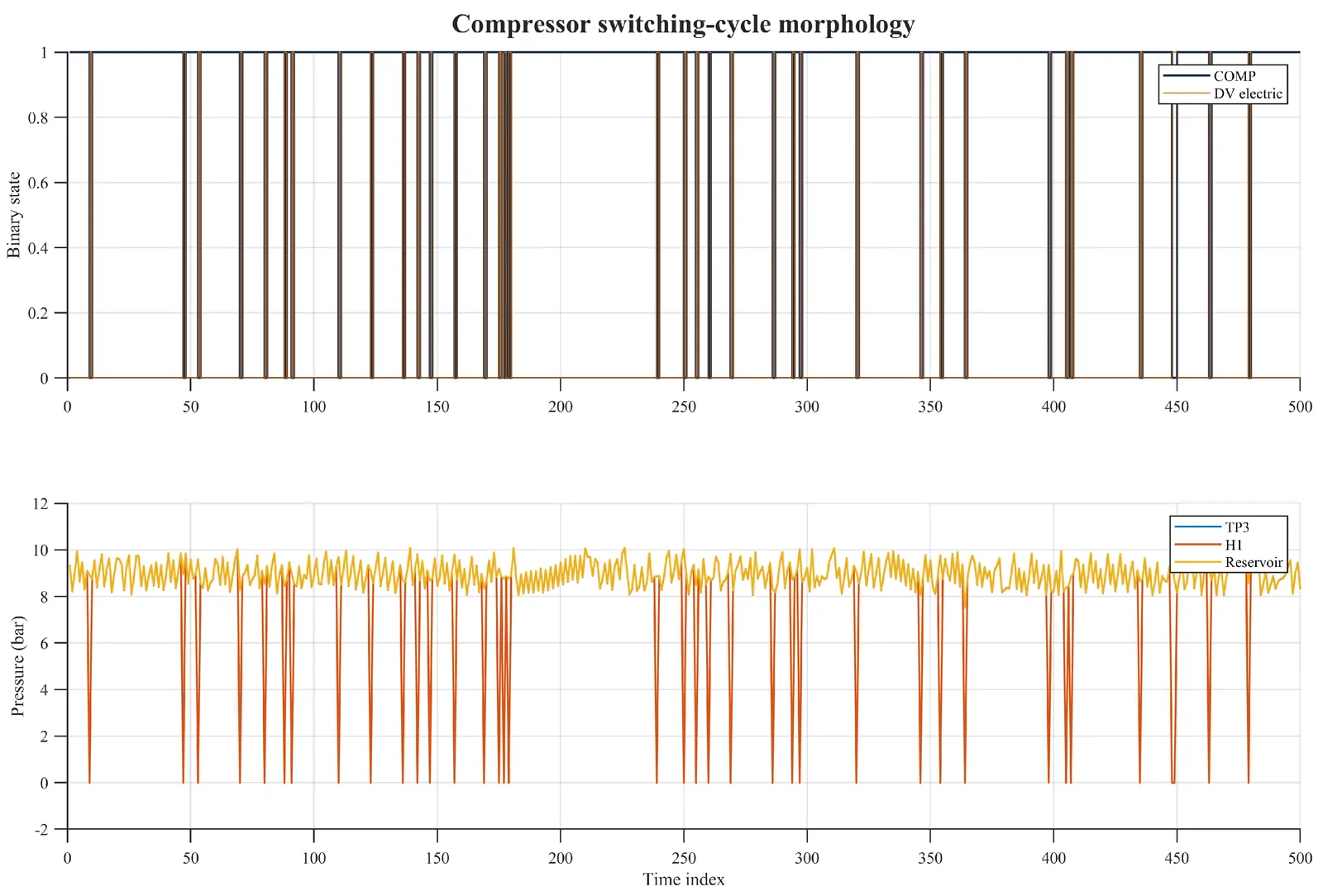
Compressor switching-cycle morphology coupling binary control states with pressure collapse and recovery.

Controller switching in Figure 11 coincides with sharp pressure responses within regulated operating bands. SDA2D represents their local motion through level–increment coupling, while temporal predicates assess control-response order. Persistent departures across control cycles and brief switching excursions consequently enter different parts of the trajectory evidence.

**Figure 12:**
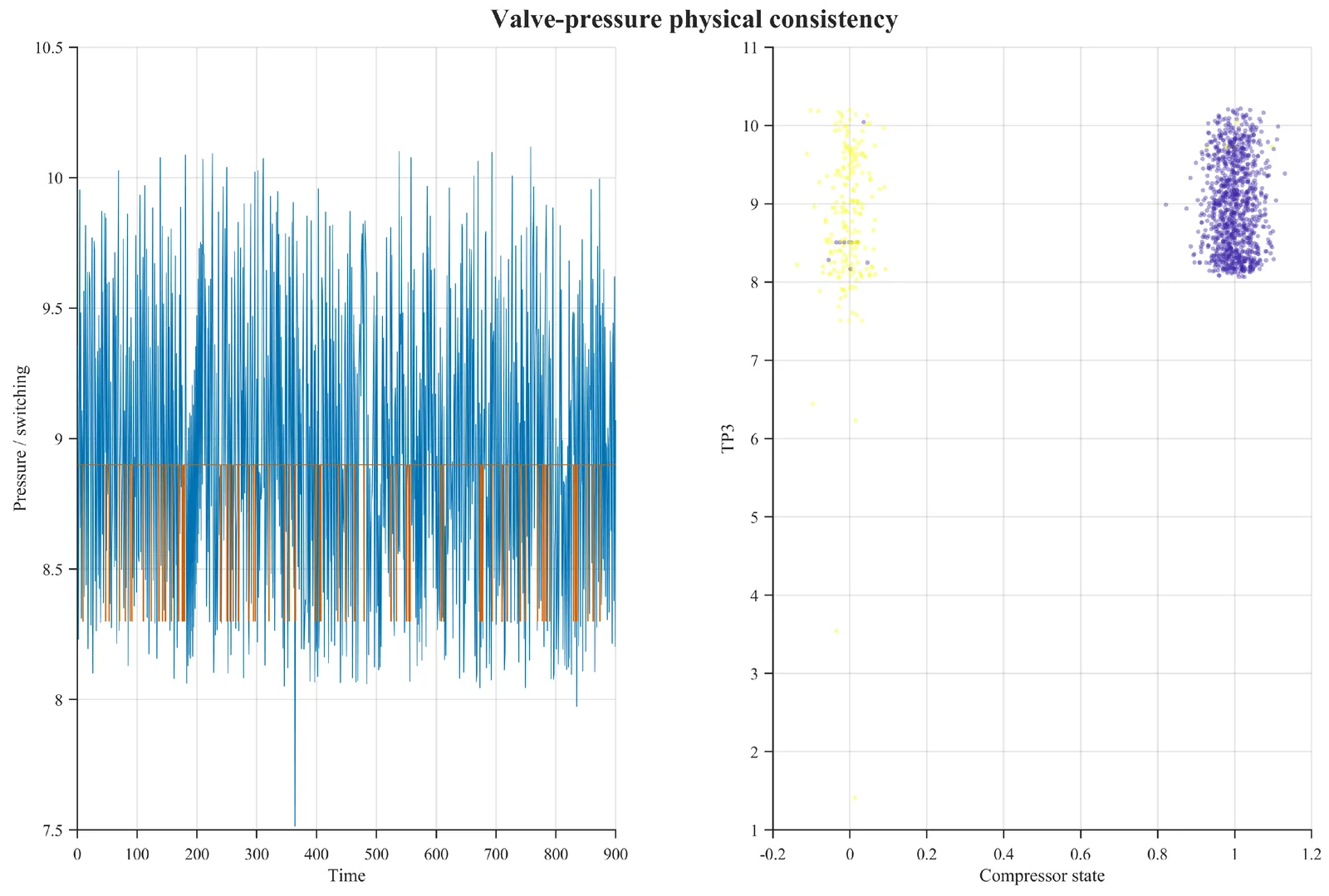
Valve-pressure consistency across loaded, unloaded, and transitional compressor states. The right-hand scatter panel uses yellow for compressor-control state 0 and purple for state 1, as identified by its horizontal coordinate.

In Figure 12, state 0 has a broader low-pressure extension and state 1 a concentrated operating band. The fitted valve–pressure residual evaluates channel compatibility within this control context. Temporal predicates additionally assess switching and recovery order along a generated path.

**Figure 13:**
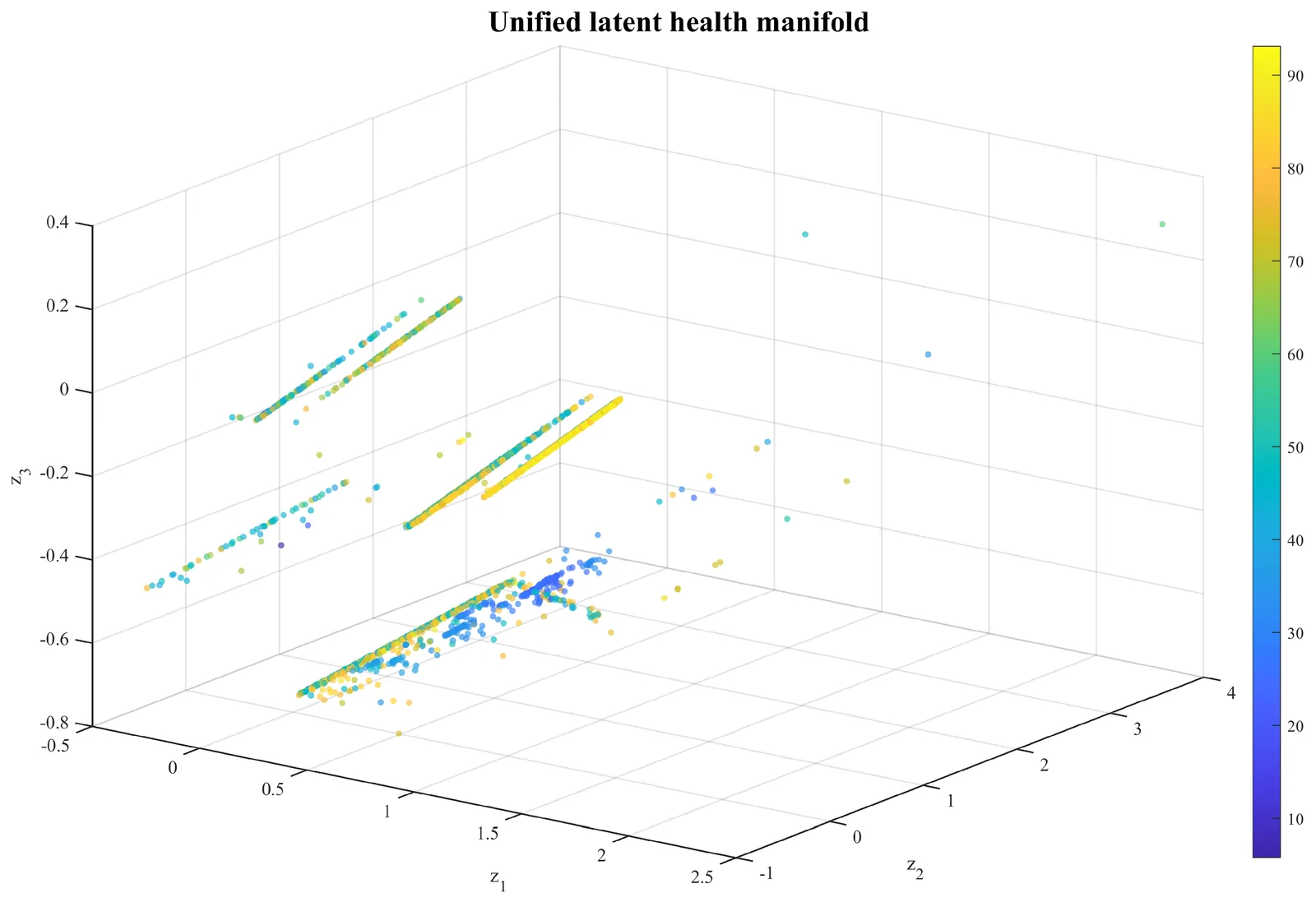
Unified latent health manifold preserving operating regimes while orienting the principal coordinate along degradation. Yellow identifies the normal end of the displayed state ordering and violet/purple the adverse end; blue, cyan, and green resolve intermediate states along the continuous colorbar.

The MetroPT3 manifold in Figure 13 retains pressure, effort, and control regimes alongside the increasing-health coordinate. Fault displacement is interpreted within this compressor-fitted basis; the scalar health readout supplies the common orientation across assets.

Table 4 separates record-level PHM2024 diagnosis from MetroPT3 controlled health-path reconstruction. Diffusion-RarePHM attains 98.23% accuracy, 98.16% macro-F1, and 0.9985 AUROC; trajectory averages are 2.250 health percentage-point RMSE, 2.294% MAPE, and 90.00% reference coverage. The 44.8 M-parameter forecast pipeline has a latency of 46.2±1.1 ms (mean ± execution-call SD) for 500 candidates and 100 DDIM updates. Appendix C specifies timed tasks and measurement boundaries.

The shared-operator deterministic rollout is the construction comparator: with the reference-side initial state and exact prescribed intervention, it gives ${\hat{H}}^{\mathrm{SO}}=H^{\mathrm{ref}}$, hence zero RMSE and MAPE by construction. Table 4 compares learned reconstruction of these specified paths. Its learned latent dynamics world model and Table 5’s retrained deterministic decoder are distinct prediction branches. Diffusion-RarePHM’s trajectory error, candidate acceptance, interval coverage, and risk statistics characterize reconstruction and accepted-ensemble behavior under the shared dynamics and intervention laws.

References in the method column of Table 4 identify the architectural sources. The authors computed every reported metric in the common implementation framework, using the shared PHM2024 manifest for diagnosis and the shared MetroPT3 origins, reference paths, time grid, and seeds for prognosis. Table A11 and Table A12 specify the configured cores and author-implemented task adapters; Appendix C defines optimization, model selection, and timing. Table 6 provides the complete model’s numerical configuration. The citations therefore establish method identity, while the declared experimental protocol establishes the basis for comparing the reported values.

Table A7 evaluates operating-condition separation. Diffusion-RarePHM attains 97.86±0.12% accuracy, 97.77±0.13% macro-F1, and 0.9979±0.0003 AUROC. Its accuracy remains within 0.37 percentage points of the record-level result and exceeds the PHM2024 encoder ablation by 3.65 points, with a macro-F1 gain of 3.79 points. Table A7 additionally reports three alternative held-out cluster pairs. Across the four pairs, accuracy ranges from 97.65% to 97.90%, macro-F1 from 97.55% to 97.82%, and AUROC from 0.9975 to 0.9980. Table A9 compares the primary pair with CatBoost on the same eight input features; its accuracy and macro-F1 exceed CatBoost by 1.45 and 1.54 percentage points, respectively, with an AUROC gain of 0.0023.

### 4.2. Conditional Multi-Future Generation and Uncertainty Geometry

Present-state discrimination does not determine how a trajectory evolves under prospective exposure. That evolution depends on the local increment and on the transition geometry inherited from normal operation. Conditional generation tests how this geometry shapes both the forecast center and the dependence among future states, before fault-directed displacement changes the underlying path.

**Figure 14:**
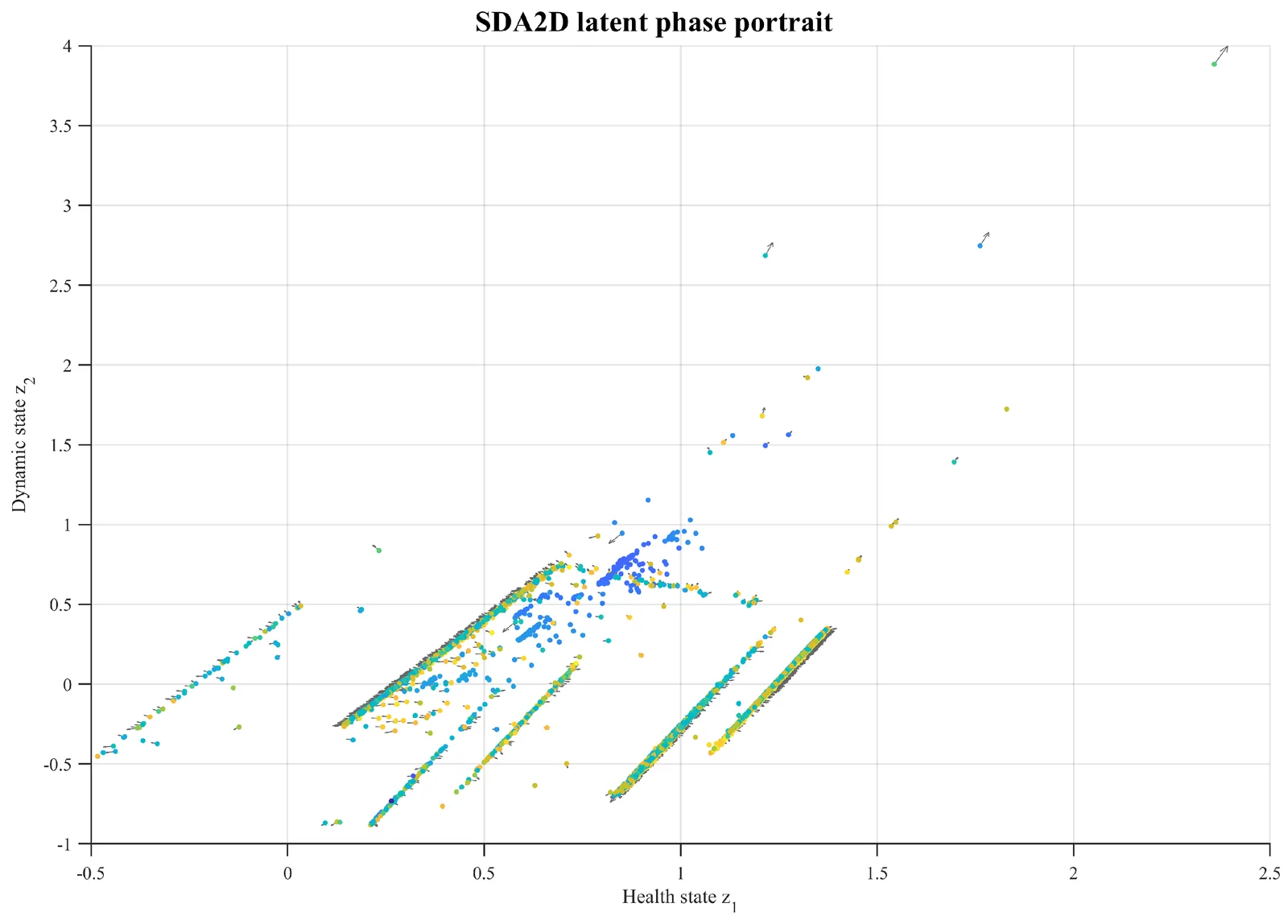
SDA2D latent phase portrait distinguishing reversible mode transitions from accumulated health evolution through level-increment coupling. Point colors retain the normal-to-adverse ordering in Figure 13: yellow marks the normal end, violet/purple the adverse end, and intervening colors resolve intermediate states. Gray arrows indicate local transition directions.

At branch intersections in Figure 14, comparable latent levels can inherit different recent trajectories. The increment resolves this local motion, and duty and environment condition its propagation. Level–increment coupling therefore represents switching, recovery, and accumulated displacement within the same state geometry.

**Figure 15:**
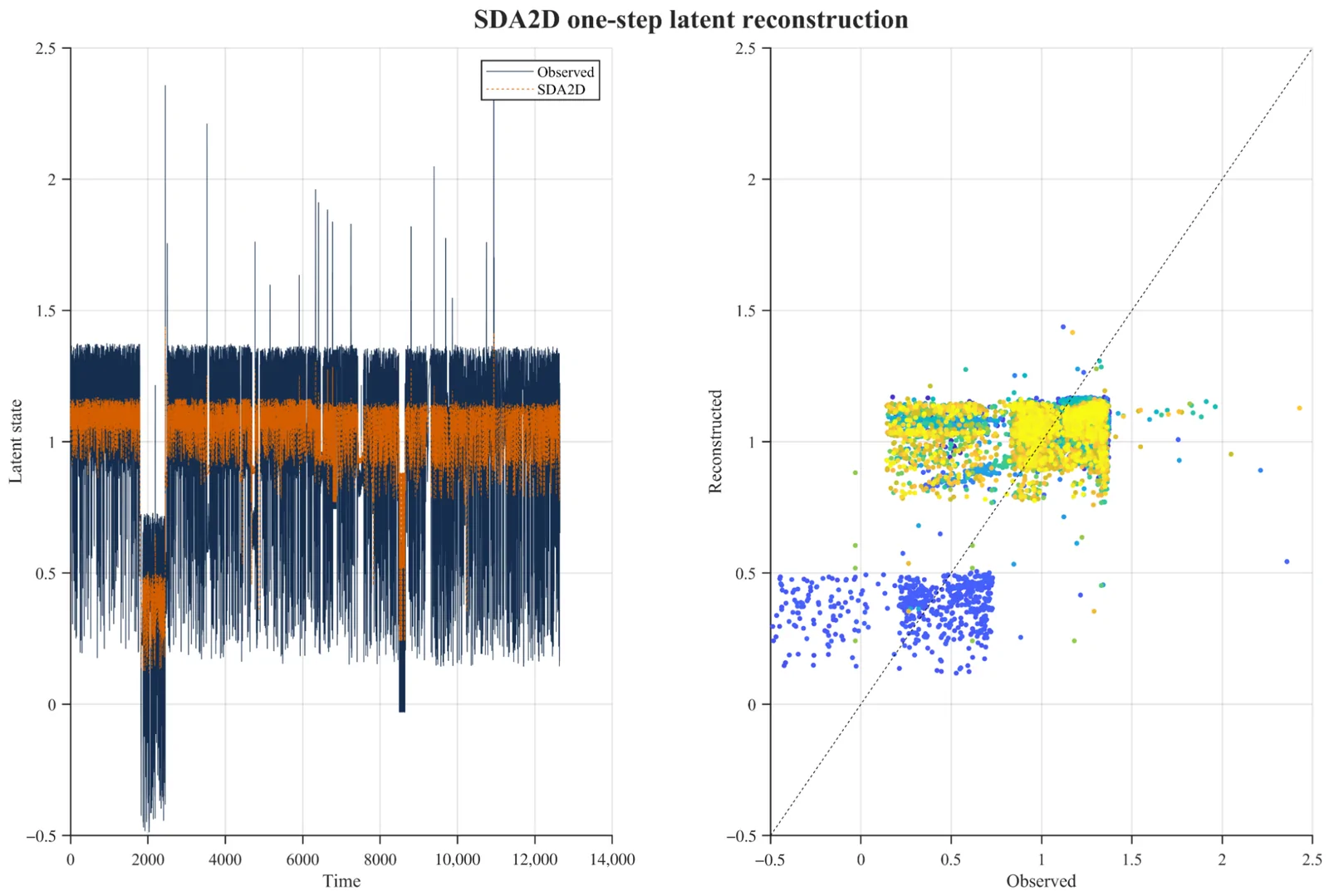
SDA2D one-step latent reconstruction and residual geometry under normal compressor dynamics. The reconstruction scatter follows the state-color convention in Figure 13, with yellow at the normal end and violet/purple at the adverse end.

Figure 15 resolves normal operating-band transitions and within-band residual variation. These residuals determine the innovation covariance and dynamic-gate scale. Holding that covariance fixed across scenarios makes differences in generated spread attributable to conditional propagation around a common stochastic geometry.

**Figure 16:**
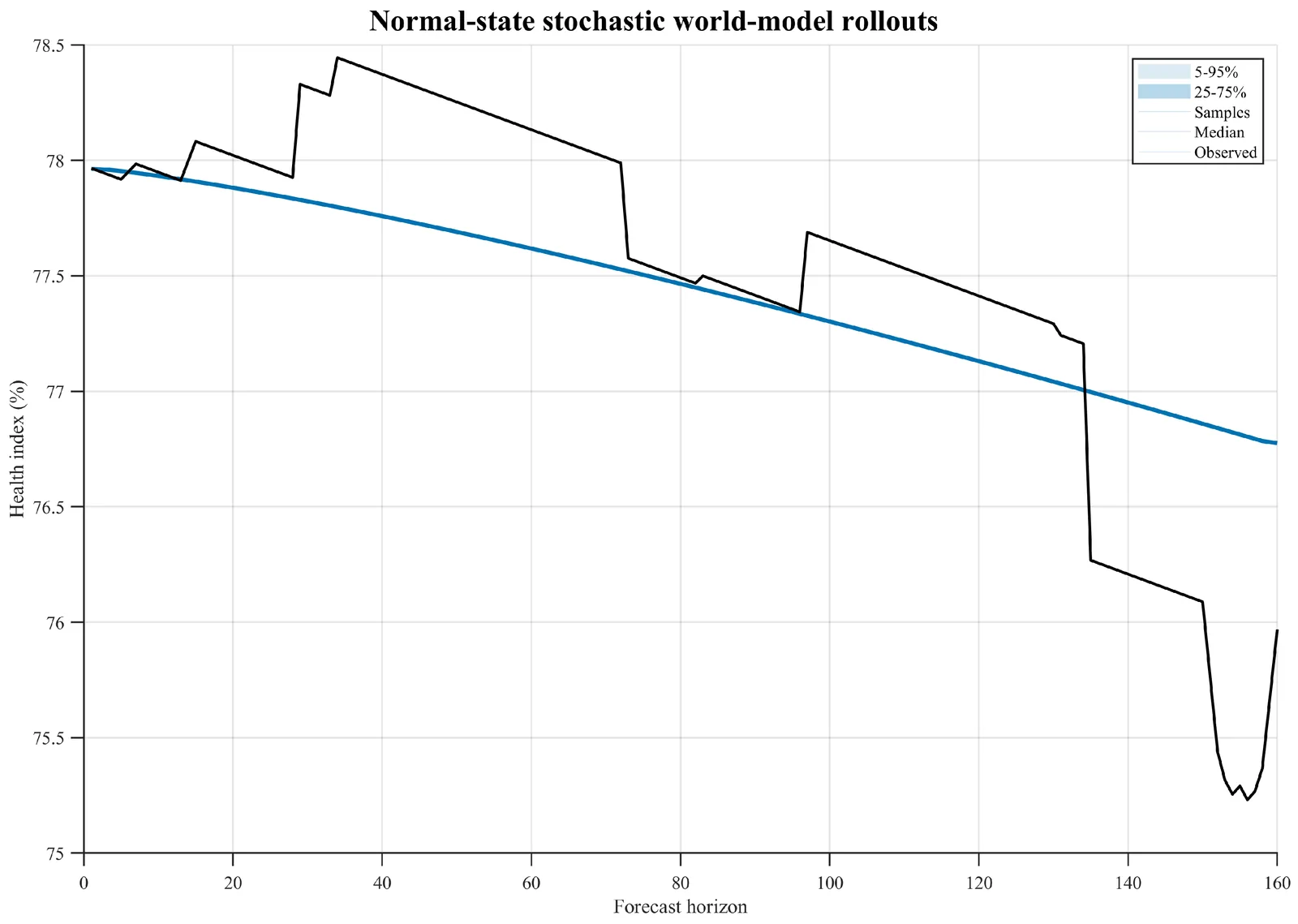
Normal-state stochastic rollouts and constructed health reference under the shared-operator dynamics, with nested predictive intervals.

The normal rollout in Figure 16 starts from a fixed state and develops conditional path dispersion around the fitted transition. Its RMSE is 0.85 health percentage points and candidate acceptance is 97.0%, providing the controlled reference for the intervention comparisons.

**Figure 17:**
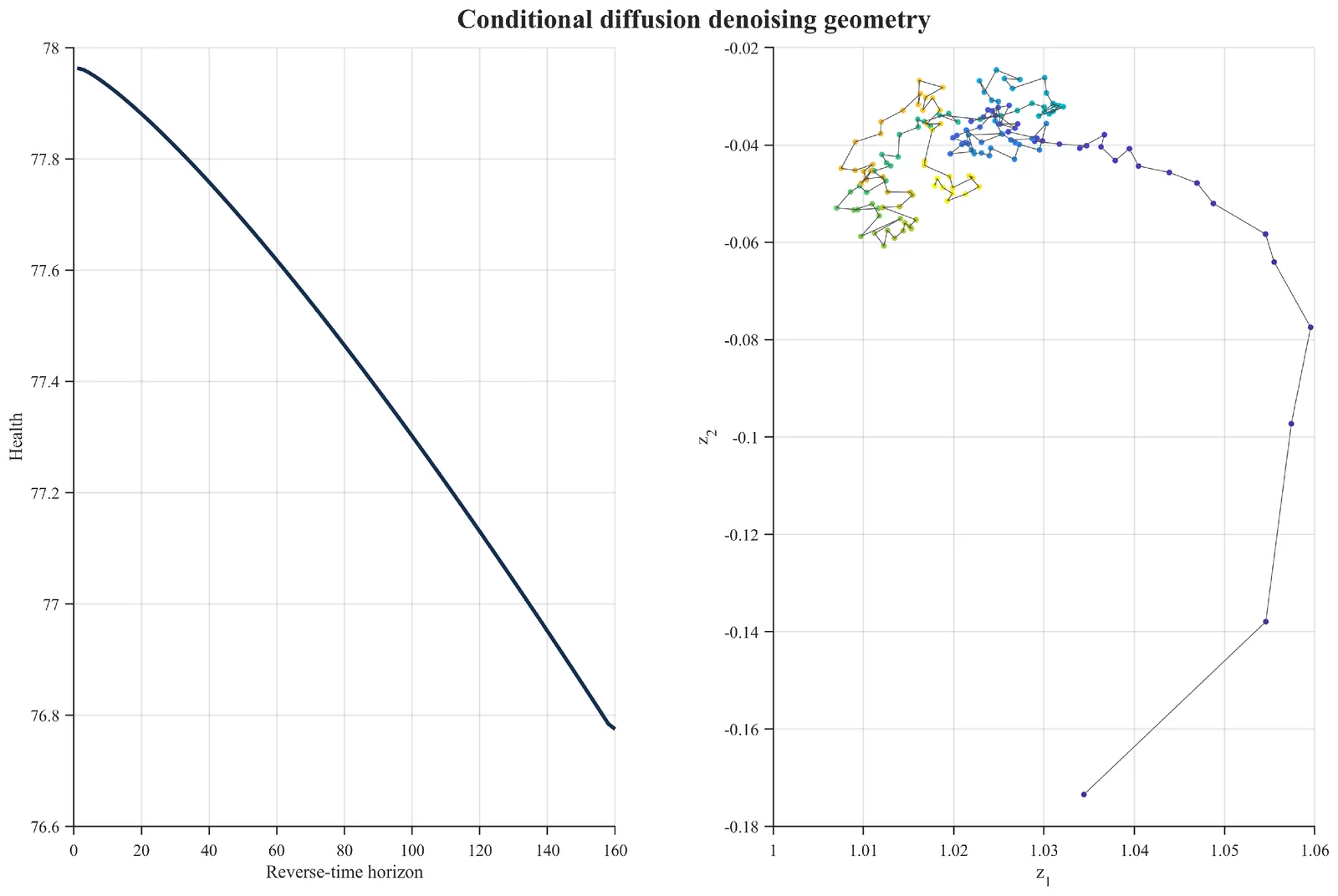
Latent geometry of a conditionally generated future, combining global evolution with local state variation.

The curved path in Figure 17 combines sustained displacement with local operating motion. Conditional generation retains this temporal structure across candidates whose transition details and terminal severity differ. Physical and semantic screening evaluate their complete sequences.

**Figure 18:**
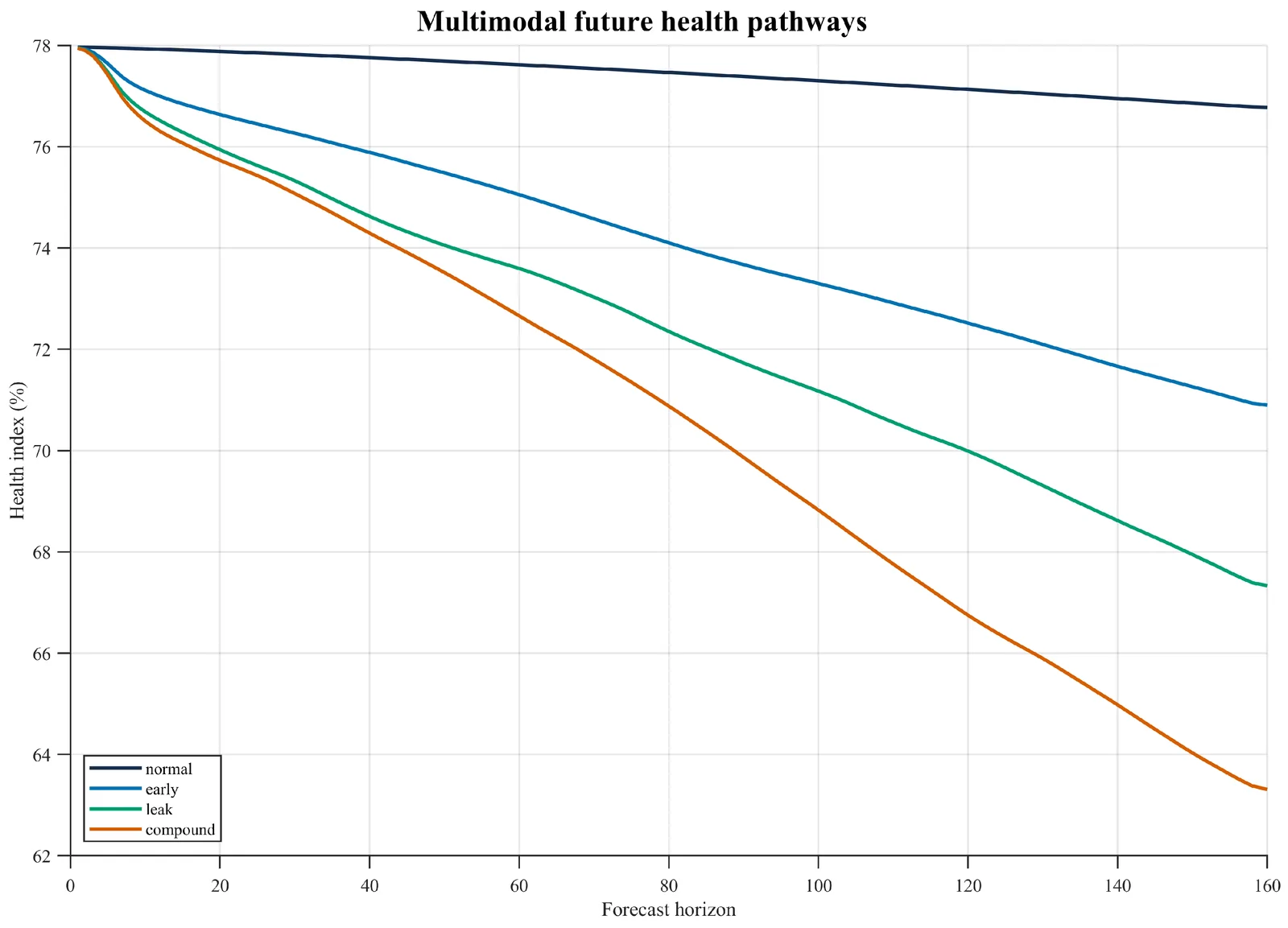
Multimodal health futures under normal, incipient-fault, leakage, and compound-fault conditions.

Figure 18 compares normal, incipient, leakage, and compound futures from a common starting state and innovation geometry. Prescribed onset and accumulation profiles produce different curvature and threshold-crossing timing. Warning, first-passage, and terminal-loss statistics retain these temporal differences within the controlled construction.

**Figure 19:**
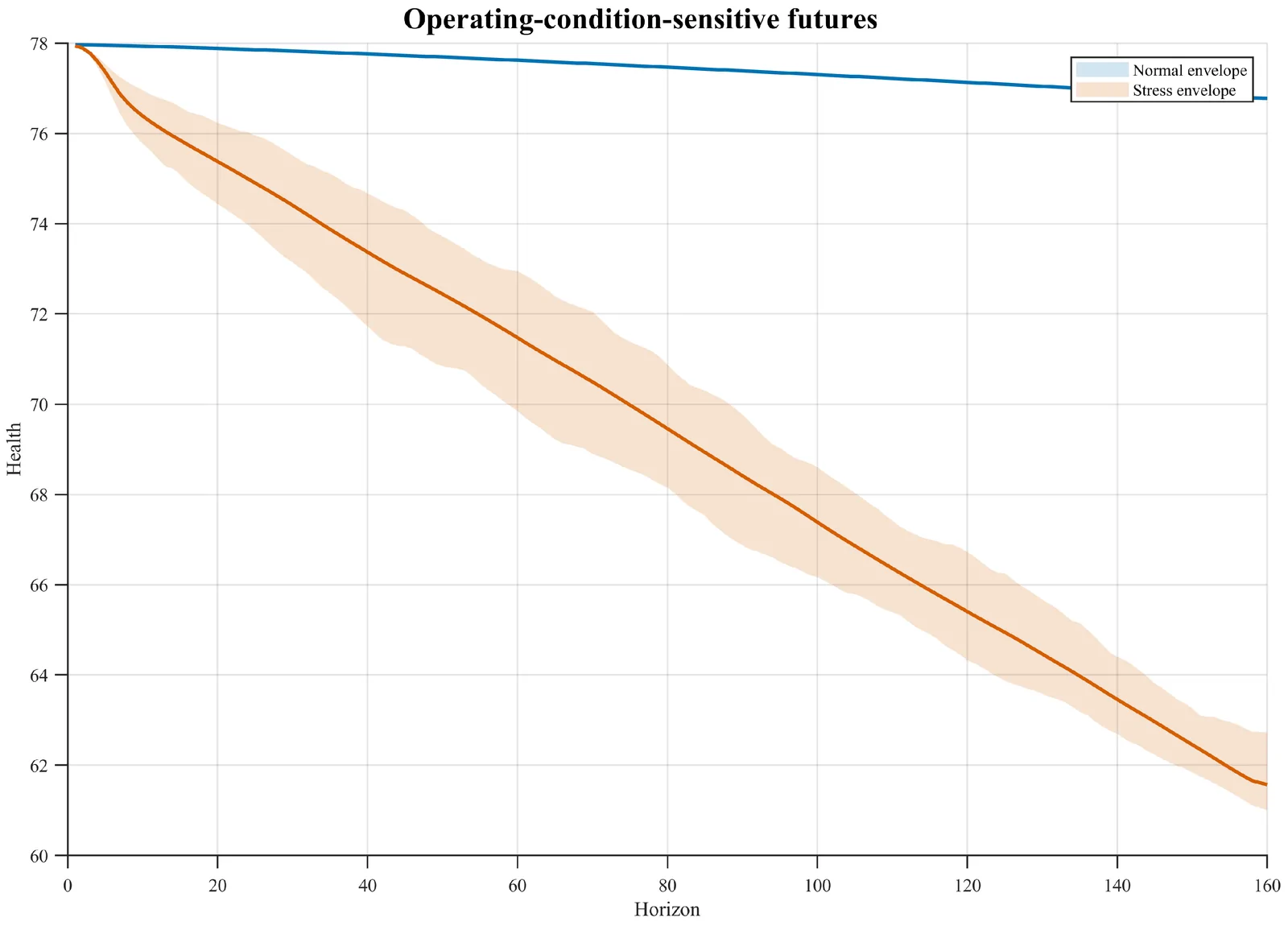
Operating-condition-sensitive futures under coupled thermal and load stress.

Duty and thermal exposure alter the future transition in Figure 19, while the initial evidence and innovation covariance remain common. Successive condition-dependent transitions can amplify or contract latent variation, changing both trajectory location and ensemble spread.

**Figure 20:**
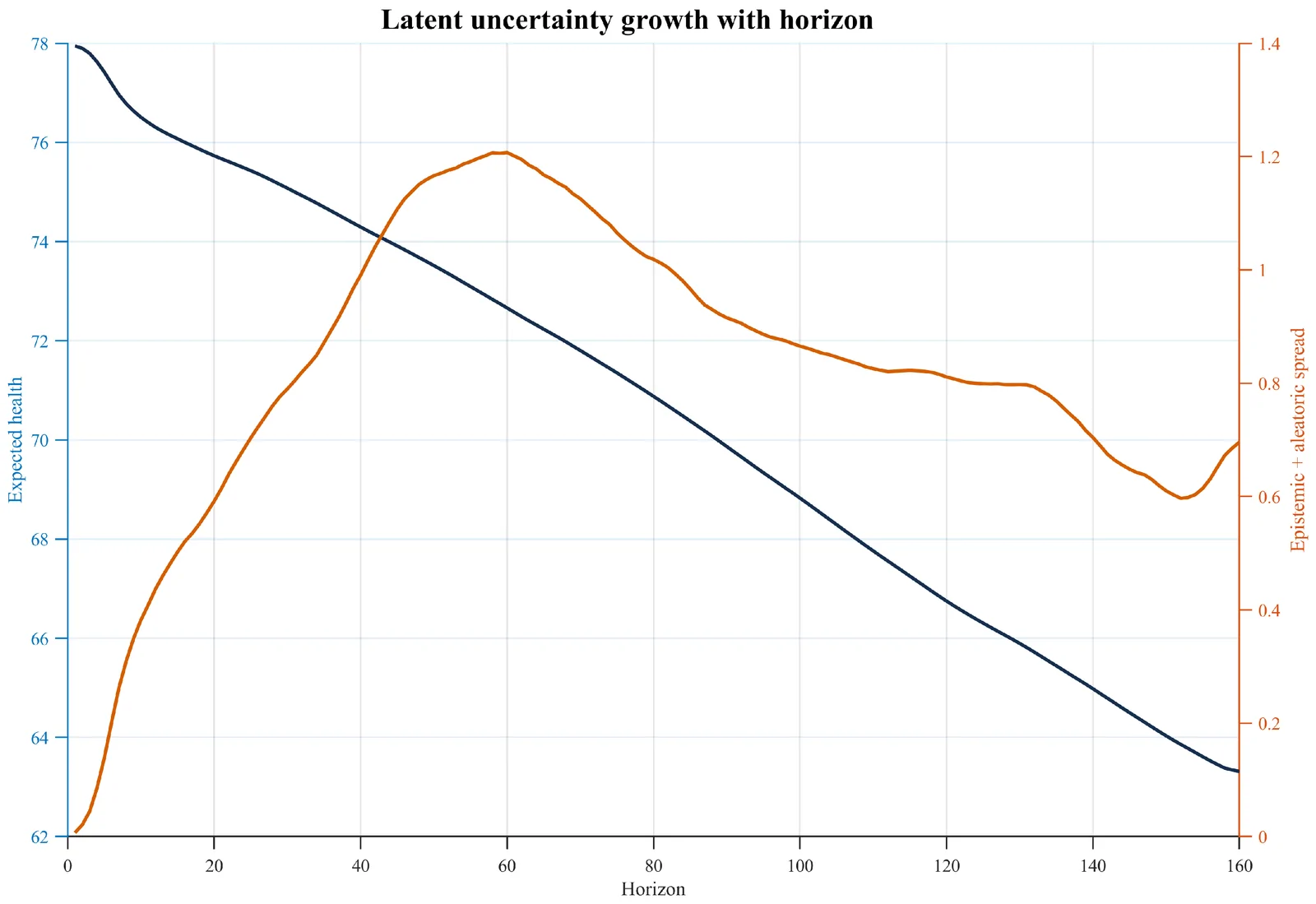
Horizon-dependent uncertainty growth constrained by SDA2D dynamics and physical health bounds.

Dispersion in Figure 20 first increases and then contracts while mean health continues to decline. This response reflects state-dependent propagation and the bounded health readout. Its risk interpretation depends on adverse quantiles and distance to the health thresholds.

**Figure 21:**
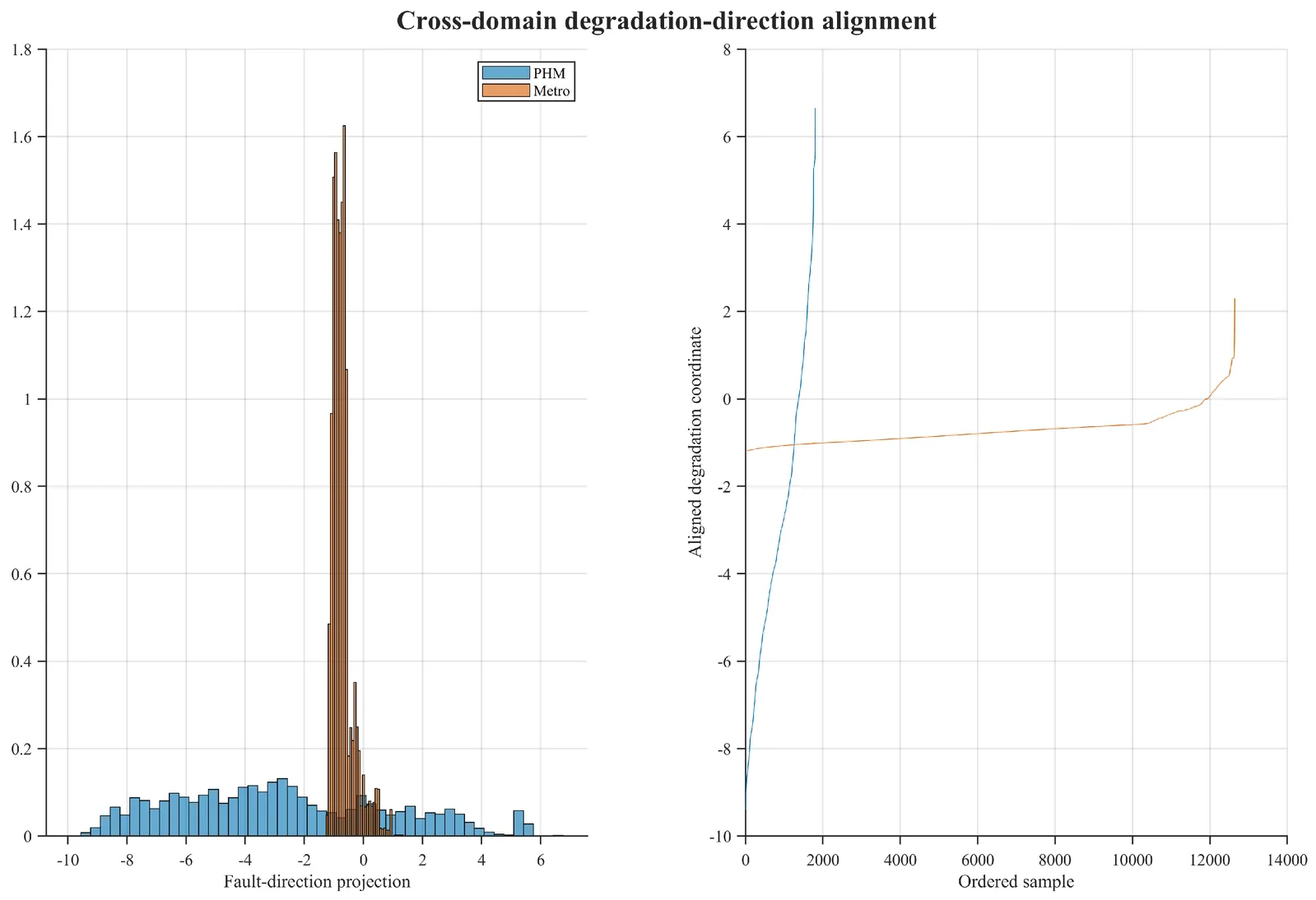
Asset-specific latent densities and ordered fault-direction coordinates within the common health interface.

Figure 21 contrasts the engine’s broad operating envelope with the compressor’s recurrent regime geometry. Cross-asset comparison uses component-wise health orientation, while local displacements retain their fitted bases. Condition-held-out diagnosis and asset-specific evidence configurations are reported in Table 2 and Table A7.

Table 5 compares the fitted diagnosis and forecast branches associated with each ablation configuration. Replacing the unified encoder with direct sensor fusion decreases PHM2024 diagnostic accuracy by 5.89 percentage points. Replacing the MetroPT3 SDA2D transition with a state-only predictor increases trajectory RMSE by 0.64 points. Deterministic extrapolation lowers 90% interval coverage to 85.6%. The gate-removal rows are marked with an em dash because those cells are not gate-pass estimates for the modified acceptance sets. Uncalibrated intervals attain 82.30% coverage. The PHM2024 entries quantify held-out fault-label discrimination by the corresponding classifiers; the MetroPT3 entries quantify trajectory reconstruction, reference inclusion, and candidate passage under the declared forecast configuration.

For Table 2 and Table 5, superscript *A* identifies $100N_{\mathrm{full}-\mathrm{pass},\mathrm{retained}}/N_{\mathrm{retained}}$, evaluated within accepted paths. Superscript *C* identifies $100N_{\mathrm{full}-\mathrm{pass},\mathrm{candidate}}/N_{\mathrm{generated}}$, auditing all proposals against the original physical, dynamic, and semantic rules. Each candidate denominator is 128,000 per scenario and seed, and reported rates average those scenario–seed audits. The full-model $100.0{\%}^{A}$ coincides with Table 3’s accepted-set admissibility; its candidate intersection rate is instead 93.72%. The three individual gate-removal rows report retained counts but no full-rule audit percentage.

At the selection stage, removing gate *u* replaces Equation (15) by $G_{-u}\left(\hat{Z}\right)=\prod_{v\in U\setminus \{u\}}⊮\left[g_{v}\left(\hat{Z}\right)\leq \kappa_{v}\right]$, where U={phys,dyn,sem}. Removing the complete validator retains the unscreened candidate pool. These changes alter the conditioning event in Equation (16) and the population used for interval and risk readout. The calibration ablation instead removes $L_{\mathrm{cal}}$ from Equation (20), retrains the model, and reads raw intervals from its accepted paths. It therefore tests the calibration objective’s effect on the learned forecast as well as on its interval endpoints.

All ablations are scored against the same physical health reference $H^{\mathrm{ref}}$, constructed by the deterministic normal SDA2D rollout and prescribed interventions using the full-model parameters fitted on blocks 0–421. Encoder, transition, and fault-direction substitutions alter the prediction branch while preserving this reference. RMSE, MAPE, and coverage therefore compare predictions against identical origin–horizon targets.

The three individual gate removals reuse the full-model candidate pool and the remaining fixed hard predicates. Their interval-calibration stages are refitted. Table A4 gives their retained counts directly: deleting physical, dynamic, or semantic validation adds $n_{011}$, $n_{101}$, or $n_{110}$ to $n_{111}$, respectively. The resulting mean counts per scenario and seed are 120,768.000, 121,651.911, and 123,741.156. The deterministic variant trains a forecast decoder and obtains its intervals from calibrated residuals. Table 7 applies sensor perturbations only during evaluation, using the trained configurations. Its complete-validator removal retains all proposals, whereas the weighted-sum configuration in Table 5 replaces the intersection with score-based selection.

The PHM2024 encoder ablation retrains the sensor-fusion representation and diagnosis head on official development records. Its frozen-encoder reference retains the Stage I checkpoint and refits a newly initialized head. MetroPT3 ablations change its history encoder, transition, decoder, displacement, selector, or calibration objective within a separate parameter block. Their synthetic trajectories and selected paths never enter the PHM2024 loader; the synthetic diagnosis proportion is zero. The derivative $\partial L_{M}/\partial \Theta_{P}=0$ excludes a diagnostic training effect from these components. Table 5 and Table 7 therefore restrict diagnosis cells to specified classifier comparisons. Prognostic substitutions are interpreted through trajectory error, constructed-reference coverage, and candidate passage. The full model retains 119,964.44 candidates per scenario and seed on average from 128,000 proposals.

The weighted selector uses $s_{\mathrm{phys}}=\exp\left(-g_{\mathrm{phys}}/\kappa_{\mathrm{phys}}\right)$, $s_{\mathrm{dyn}}=\exp\left(-g_{\mathrm{dyn}}/\kappa_{\mathrm{dyn}}\right)$, and $s_{\mathrm{sem}}=\exp\left(-g_{\mathrm{sem}}\right)$. All three scores decrease with violation magnitude. A path is retained when $(s_{\mathrm{phys}}+s_{\mathrm{dyn}}+s_{\mathrm{sem}})/3>0.5$.

### 4.3. Controlled Rare-Fault Demonstrations and World-Model Self-Validation

A coherent normal forecast supplies the local reference for interpreting fault interventions. Prescribed displacement changes the mechanical path; drift and missingness change the evidence used to infer it; maintenance modifies accumulated displacement while preserving prior events. Comparing these responses under common dynamics and covariance reveals whether generation preserves their distinct temporal and mechanistic roles.

**Figure 22:**
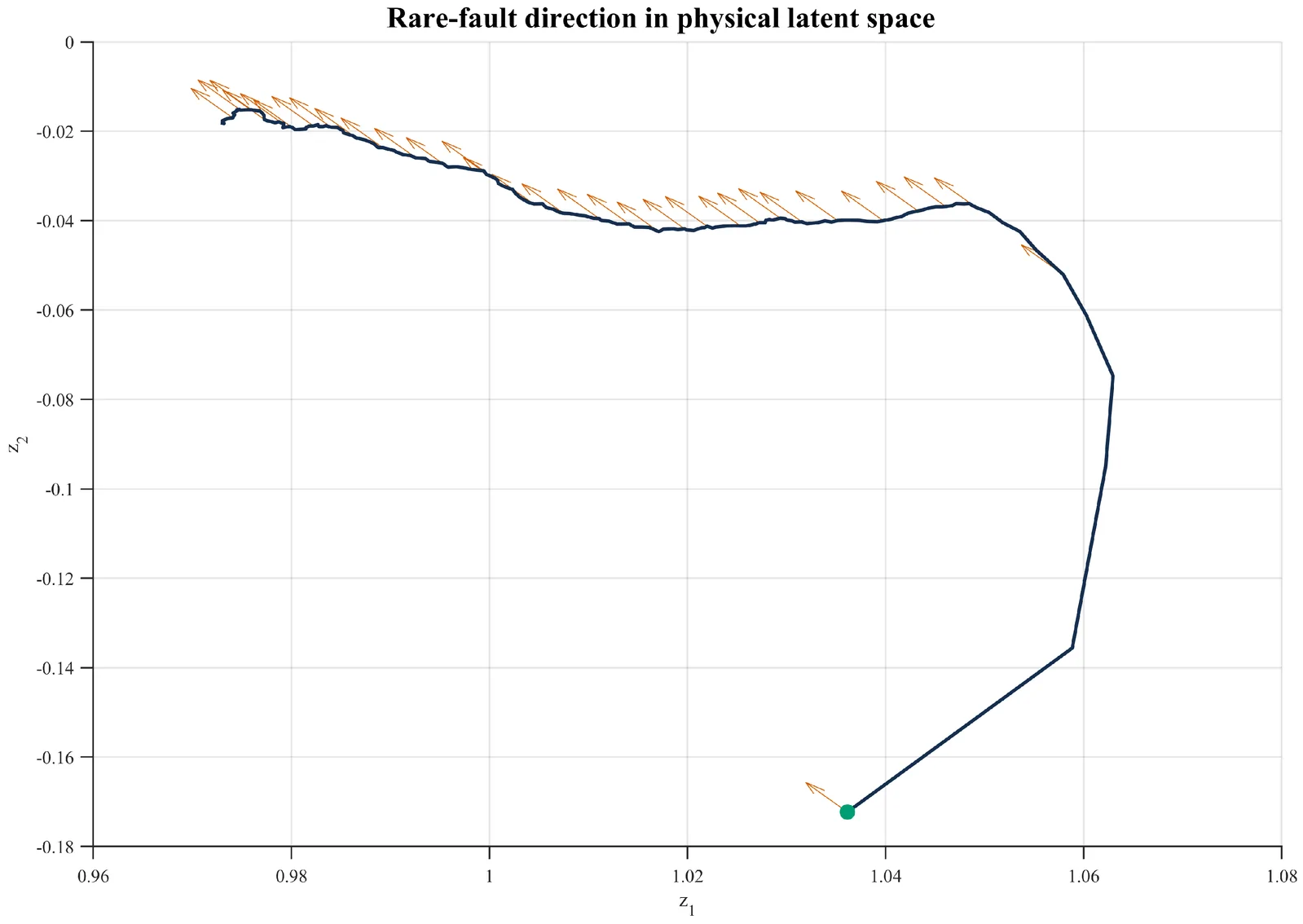
Controlled fault-direction displacement along dynamically reachable regions of latent space.

**Figure 23:**
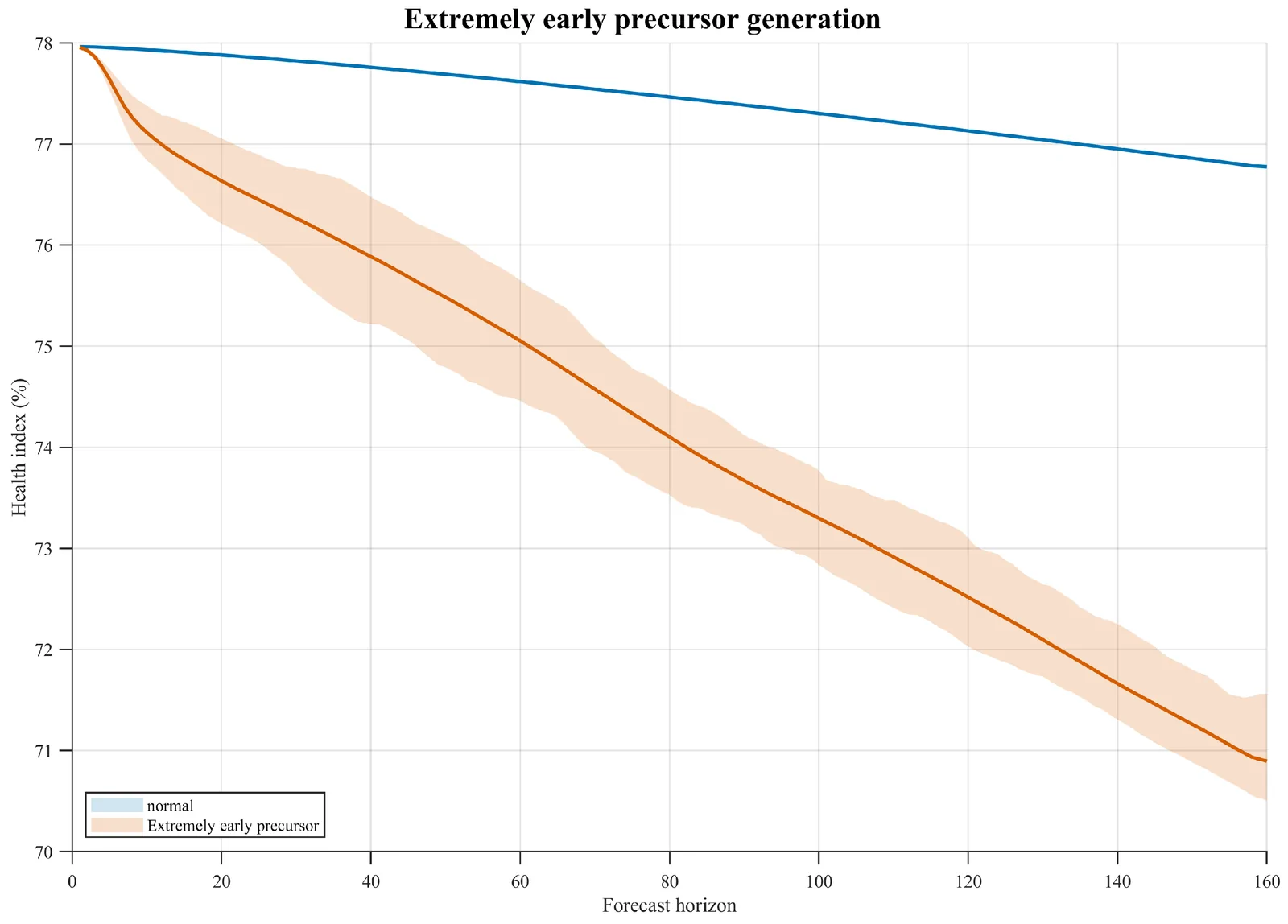
Controlled incipient-fault trajectories separating from normal evolution under the prescribed onset profile.

**Figure 24:**
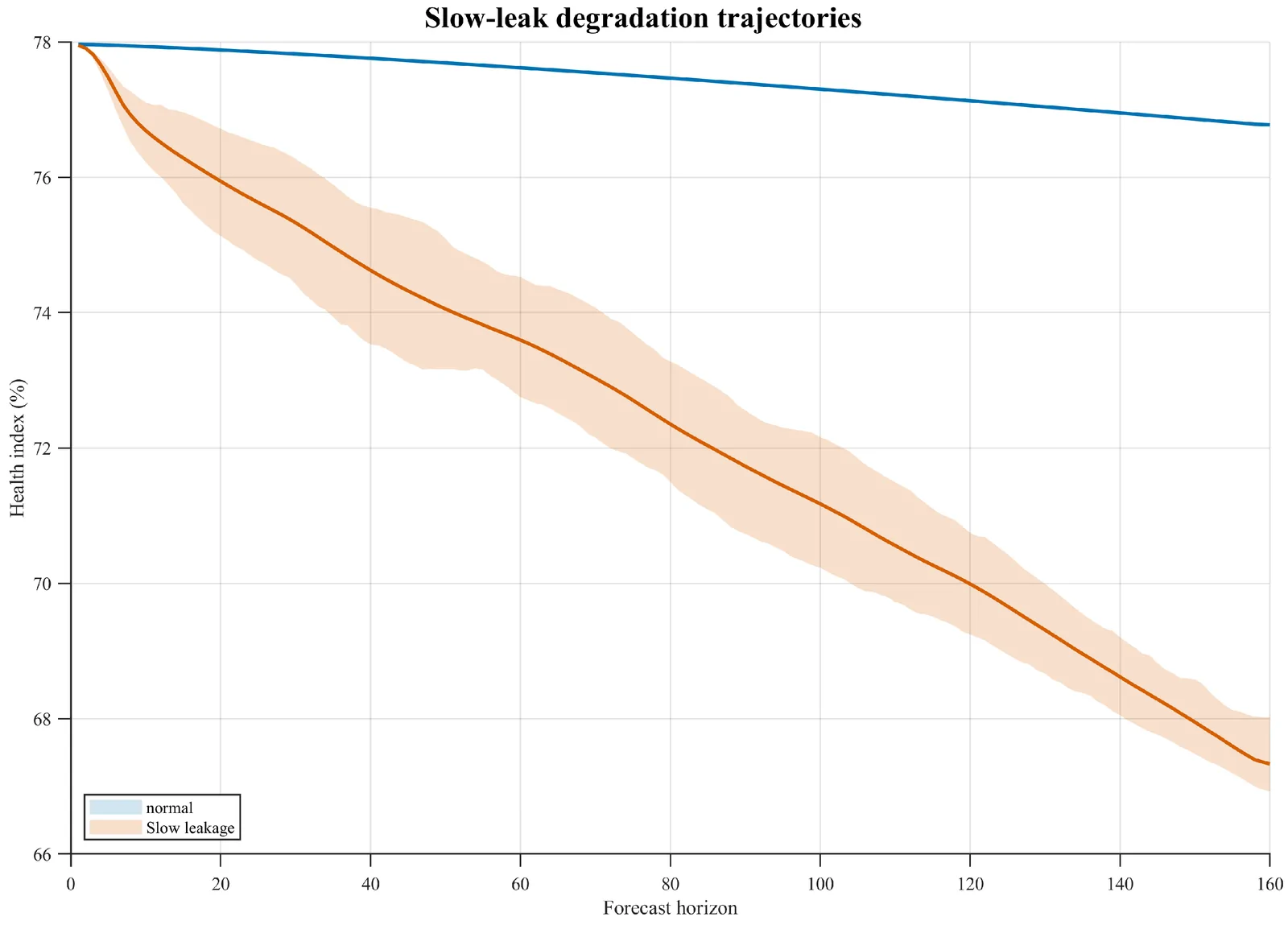
Controlled slow-leak trajectories under the prescribed cumulative degradation profile.

**Figure 25:**
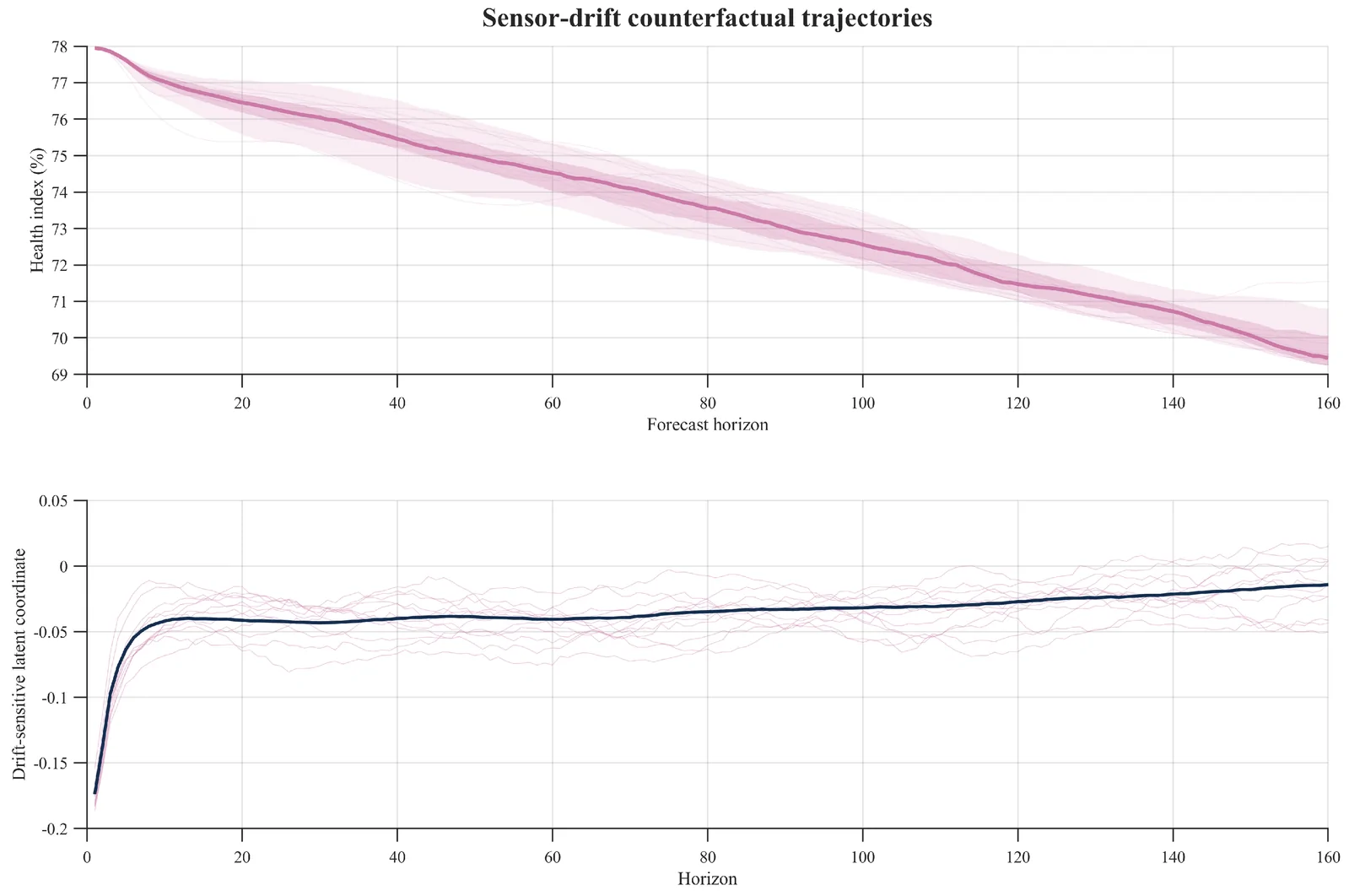
Controlled sensor-drift counterfactuals. The plotted health response is inferred from TP2-contaminated observations; the mechanical reference remains the uncorrupted normal trajectory.

**Figure 26:**
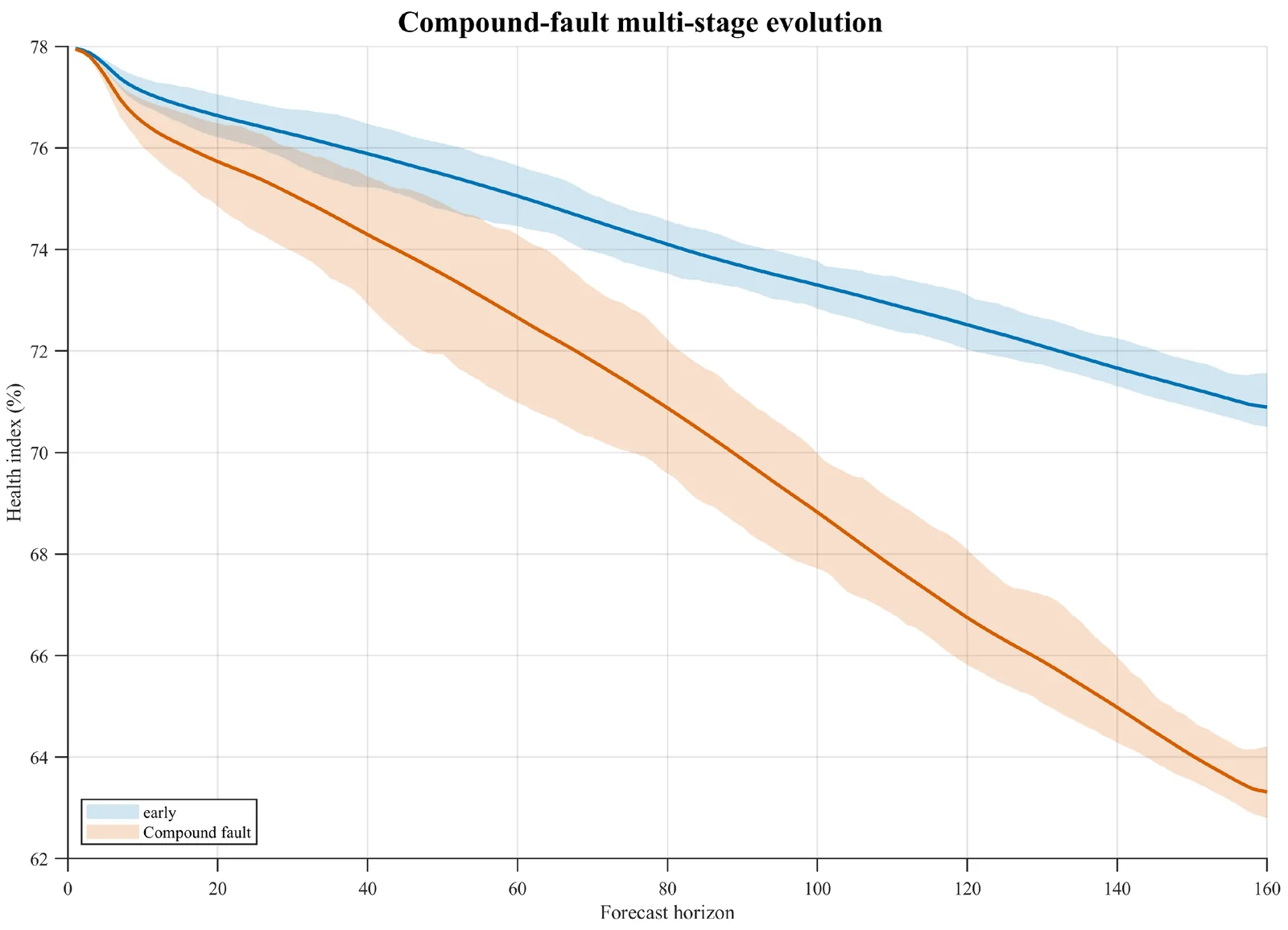
Controlled compound-fault trajectories under the prescribed precursor and secondary acceleration profiles.

**Figure 27:**
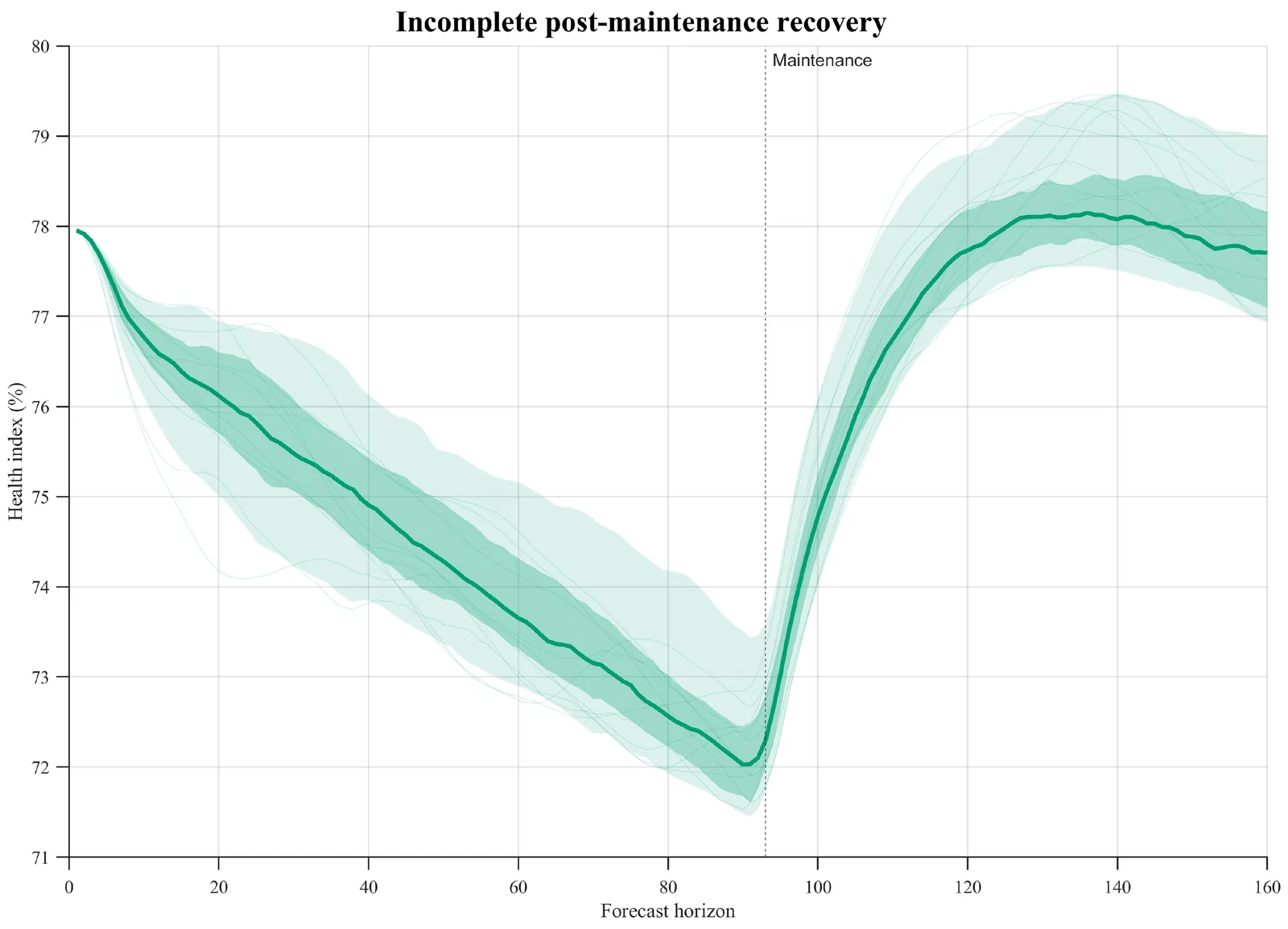
Controlled post-maintenance trajectories under the prescribed partial restoration and recovery law, with residual predictive uncertainty.

**Figure 28:**
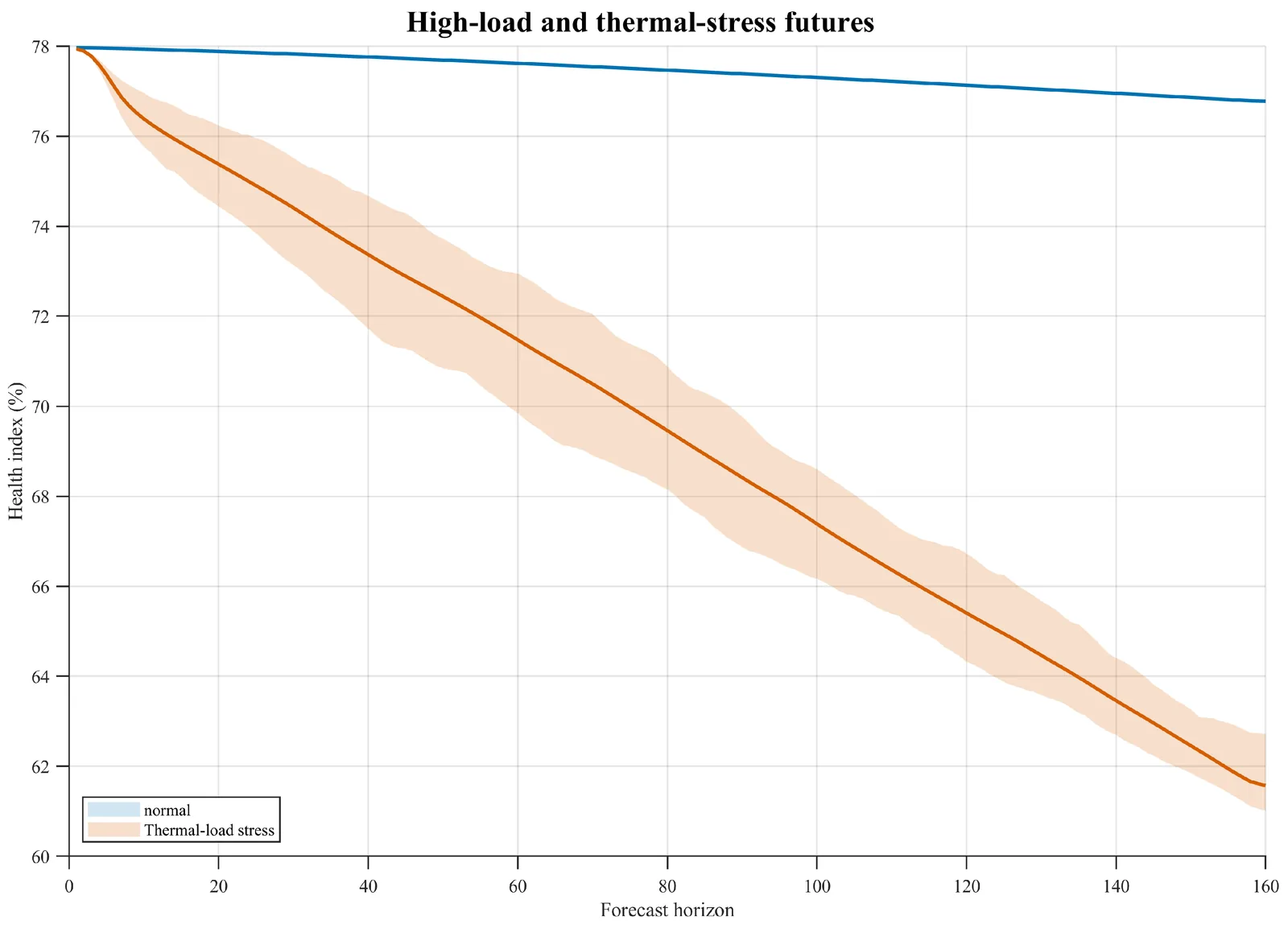
Controlled health futures under coupled high-load and thermal-stress conditioning.

**Figure 29:**
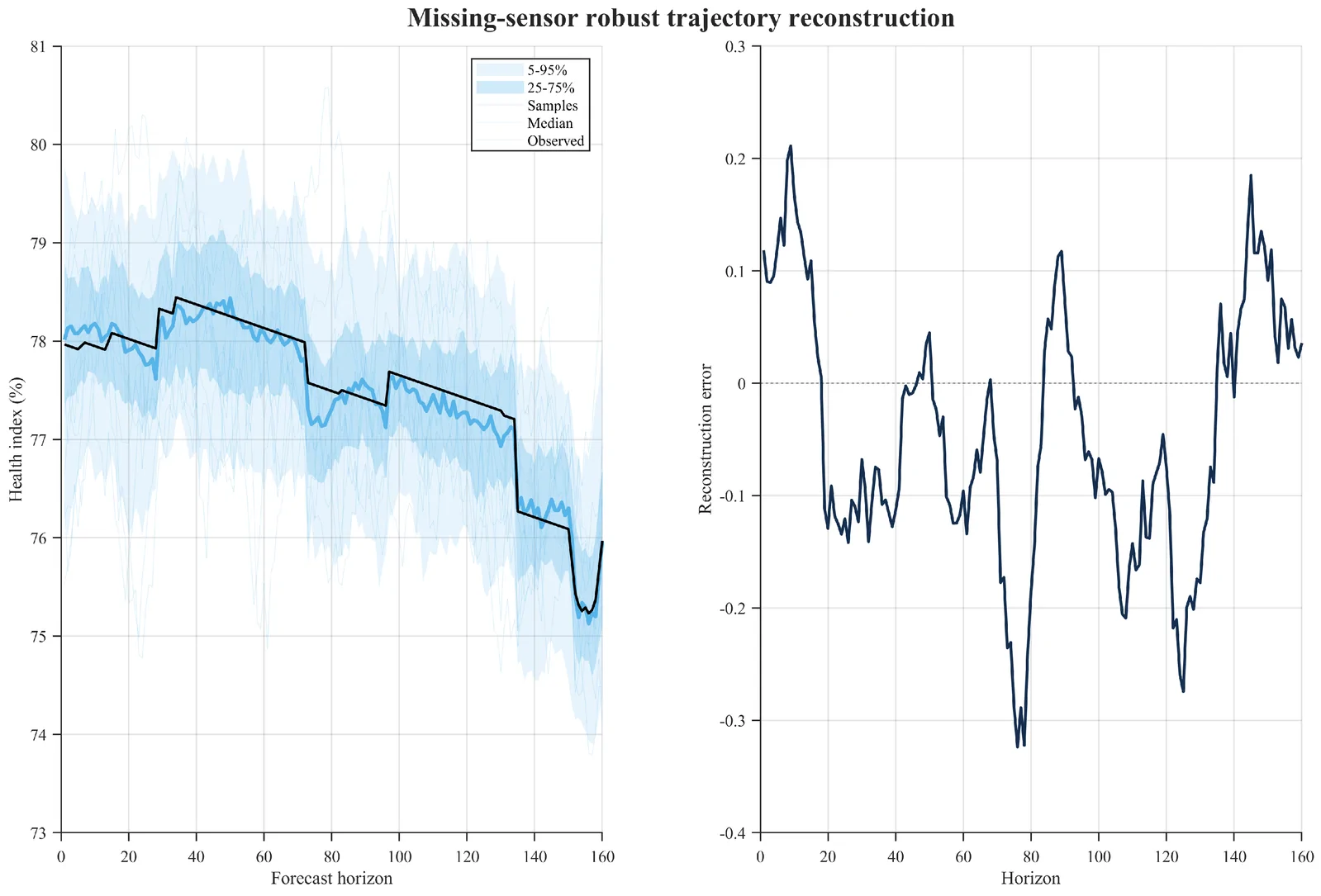
Controlled sensor-dropout reconstruction with uncertainty around the inferred health path and an unchanged mechanical reference.

Figure 22, Figure 23, Figure 24, Figure 25, Figure 26, Figure 27, Figure 28 and Figure 29 examine the generator under the prescribed interventions in Appendix A. MetroPT3 supplies the observed initial histories and the fitted normal dynamics; subsequent fault evolution, maintenance response, and mechanical health references follow the declared semi-physical construction. The experimental endpoints are the response to intervention severity and timing, temporal composition, recovery behavior, and observation corruption. Warning crossings and first-passage times refer to these constructed futures and their specified health thresholds.

Table 5 quantifies the contribution of estimated fault directions on the same reference trajectories. Relative to random-direction augmentation, the full model reduces RMSE from 2.560 to 2.250 percentage points and MAPE from 2.450% to 2.294%, while nominal 90% coverage increases from 88.10% to 90.00%. This comparison measures forecast fidelity under the benchmark’s specified mechanisms. The PHM2024 columns separately measure diagnosis against observed fault labels.

The vectors in Figure 22 displace the contemporaneous normal state along a fitted fault direction. Equation (13) specifies total displacement at each time, preserving the intervention profile without repeated offset accumulation. Residual generation adds variation around this mechanism-conditioned path.

The incipient trajectories in Figure 23 retain early overlap before sustained separation develops. Lower-tail warning crossings can therefore precede separation of the central paths. Fixed intervention laws and gates connect this precursor response to warning and critical first passage within the accepted ensemble.

The leakage law in Figure 24 combines an exponentially developing contribution with a quadratic later departure. Accepted-path variation distributes the resulting pressure-efficiency loss across crossing times. Location and adverse mass jointly describe its progression from early overlap toward the critical boundary.

Figure 25 perturbs TP2 while retaining the clean mechanical reference. The resulting forecast change measures how biased pressure evidence affects state inference and pressure-per-current health readout. Saturation of the imposed bias and continued latent propagation can produce different temporal responses.

The compound path in Figure 26 adds secondary acceleration after the precursor phase. Displacement coefficients compose in latent space, while the component-wise health map produces a state-dependent response. Semantic predicates assess this ordering, and the resulting paths determine warning duration, critical first passage, and terminal loss.

At $95^{+}$ in Figure 27, maintenance removes 55% of accumulated fault displacement; the remaining 45% decays with a 25-step time constant. Post-action health reflects both restoration and relaxation. First-passage statistics retain crossings before maintenance, whereas terminal loss records the subsequently attained condition.

Thermal-load exposure in Figure 28 changes successive transition means from a common initial state. The innovation covariance and acceptance predicates remain fixed, so the altered trajectory and adverse tail reflect condition-dependent propagation under the prescribed exposure.

Figure 29 changes observation availability while preserving the mechanical reference. For the declared eligible-channel masks, the within-two-point rate decreases from 94.5% at 20% dropout to 89.2% at 50% dropout (Table 7), a 5.3-percentage-point change under common forecast targets.

**Figure 30:**
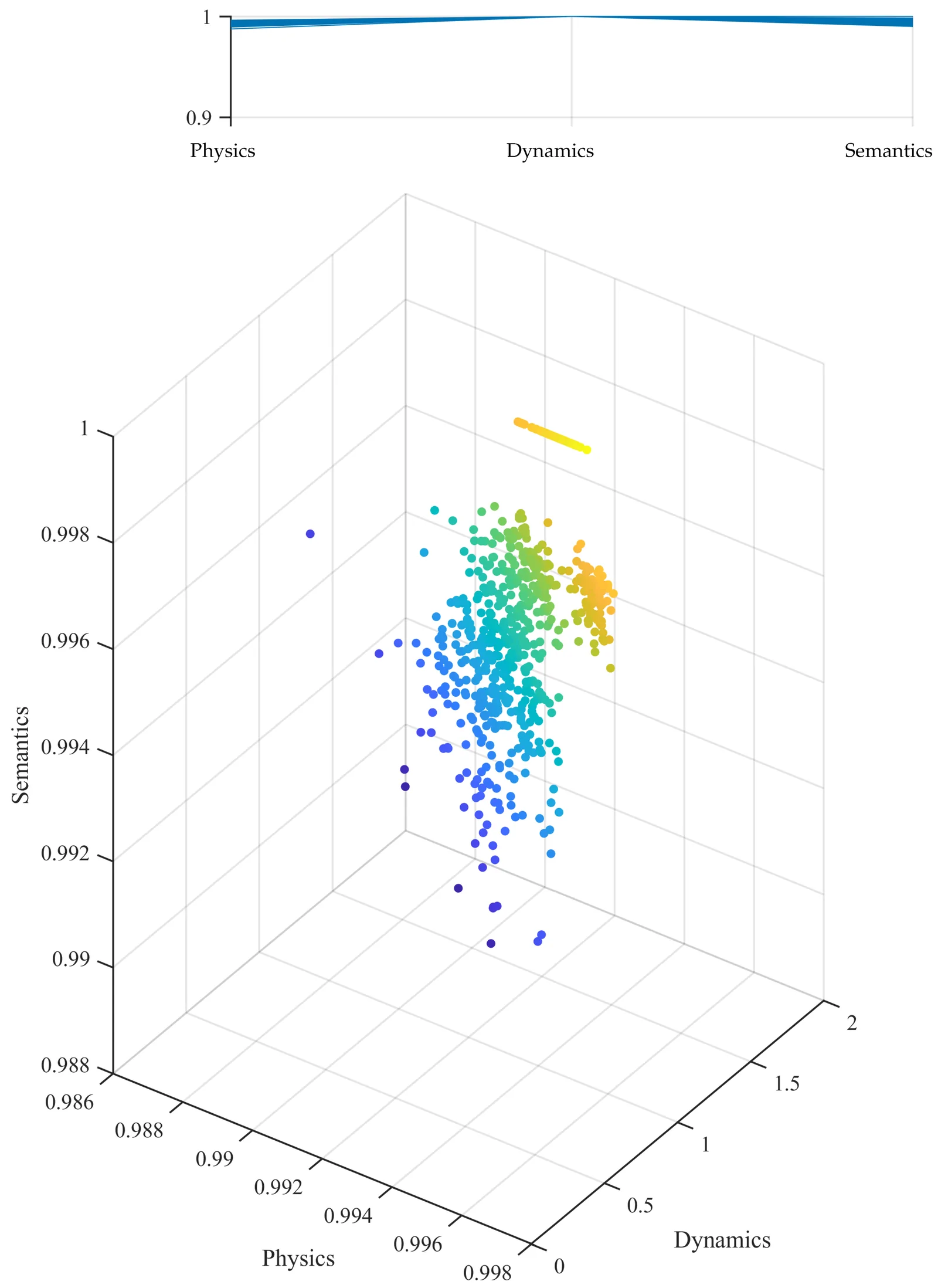
Triple-gate trajectory validation. Upper panel: enlarged criterion profile in its 0.9–1.0 coordinate band. Lower panel: joint criterion-coordinate geometry, with displayed axes spanning 0.986–0.998 for physics, 0–2 for dynamics, and 0.988–1.000 for semantics.

Figure 30 separates pressure compatibility, dynamic residuals, and event order. Semantic-only and dynamic-only failures account for 47% and 21% of rejected proposals. Joint acceptance is 93.72%, with at least 440 of 500 candidates retained at every origin; joint signatures avoid double-counting simultaneous failures.

### 4.4. Integrated Early Warning, RUL, Tail Risk, and Robustness

Path screening determines which temporal realizations contribute to decisions. Warning probability measures the accumulated crossing mass, RUL locates critical first passage, and tail loss describes severity at a selected horizon. Reading these quantities from one accepted population preserves their relationships as intervention timing and observation quality change.

**Figure 31:**
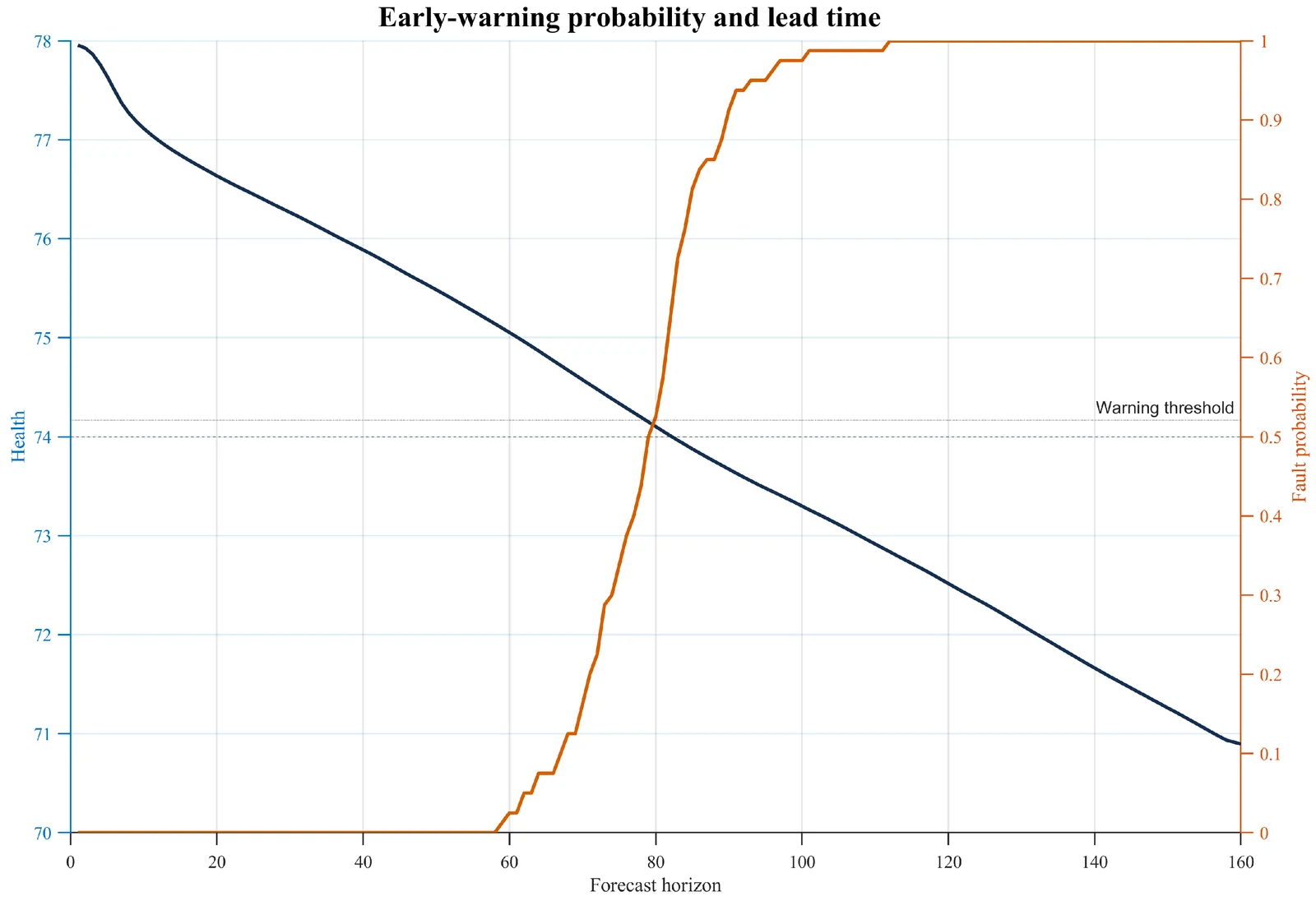
Pathwise early-warning probability and lead time derived from ensemble threshold-crossing events.

In Figure 31, adverse-path crossings raise warning mass before the central trajectory reaches $H_{\mathrm{warn}}=74$. The cumulative curve resolves the timing and dispersion of those crossings. Critical first passage and tail loss describe subsequent outcomes of the same accepted paths.

For the MetroPT3 trajectory dropout experiments, the 20% and 50% rates refer to observation cells in $C_{\mathrm{miss}}=\left\{TP2,TP3,H1,\mathrm{motor}\_\mathrm{current}\right\}$, corresponding to 5.33% and 13.33% of the full 15-channel input. Masked runs span 4–16 h.

The PHM2024 noise experiments add Gaussian perturbations with standard deviations σ=0.1 and 0.3 to the seven measured channels in standardized input space. The fitted preprocessing and classifier remain fixed during perturbation evaluation. The final two columns of Table 7 instead evaluate MetroPT3 intervals under Gaussian observation noise with σ=0.3 on all 15 standardized channels, using perturbation seed 2026. MetroPT3 masking operates on the four eligible channels specified above. These test-time observation perturbations preserve the mechanical reference, fitted model parameters, diffusion schedule, and shared innovation covariance $\Sigma_{\eta }$.

For a masking setting δ and seed *s*, the MetroPT3 within-tolerance rate is $A_{2,s}^{\left(\delta \right)}=\frac{100}{9\times 256\times 160}\sum_{f=1}^{9}\sum_{i=1}^{256}\sum_{h=1}^{160}⊮[|{\bar{H}}_{i,f,s,h}^{\left(\delta \right)}-H_{i,f,h}^{\mathrm{ref}}\left|\leq 2\right]$. Here, $\bar{H}$ is the configuration’s point forecast and the tolerance is two health percentage points. The table reports the mean and sample standard deviation of the five seed-level rates. Their equal-weight mean is also the pooled rate over 9×5×256×160=1,843,200 forecast cases.

Table 7 shows robustness within the two asset tasks. PHM2024 diagnosis retains 97.12±0.10% accuracy at σ=0.1 and 94.34±0.15% at σ=0.3. Under stronger noise, the PHM2024 encoder variant attains 88.34±0.20%. For MetroPT3, 94.5±0.2% of point forecasts remain within two health percentage points of the reference under 20% eligible-channel masking, and 89.2±0.3% do so under 50% masking. The full configuration exceeds raw fusion by 3.3 and 3.6 percentage points in these two settings. Its interval result combines 92.78±0.35% coverage with width 2.12±0.04 percentage points, compared with 83.56±0.65% and 3.45±0.07 for the raw-interval configuration. Together, the tolerance rate and interval statistics quantify the preservation of pointwise health accuracy and uncertainty concentration under sensor perturbation.

**Figure 32:**
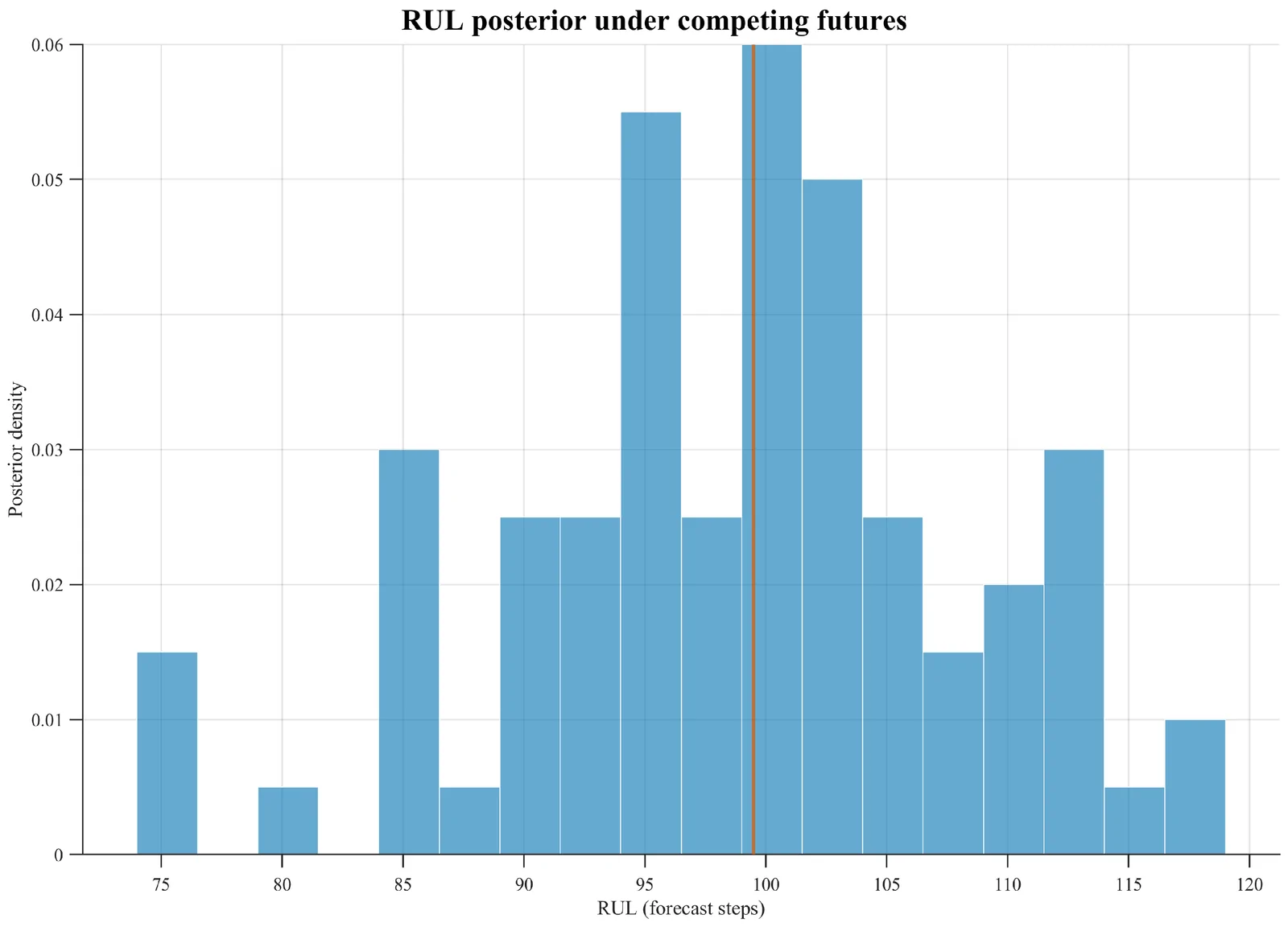
Remaining-useful-life posterior induced by competing first-passage trajectories.

Figure 32 resolves finite crossings of $H_{\mathrm{crit}}=65$ alongside paths right-censored at 320 h. Censored paths retain their distributional weight, and a RUL quantile remains beyond the horizon when its cumulative event mass is not reached. This preserves agreement between reported RUL and failure probability.

**Figure 33:**
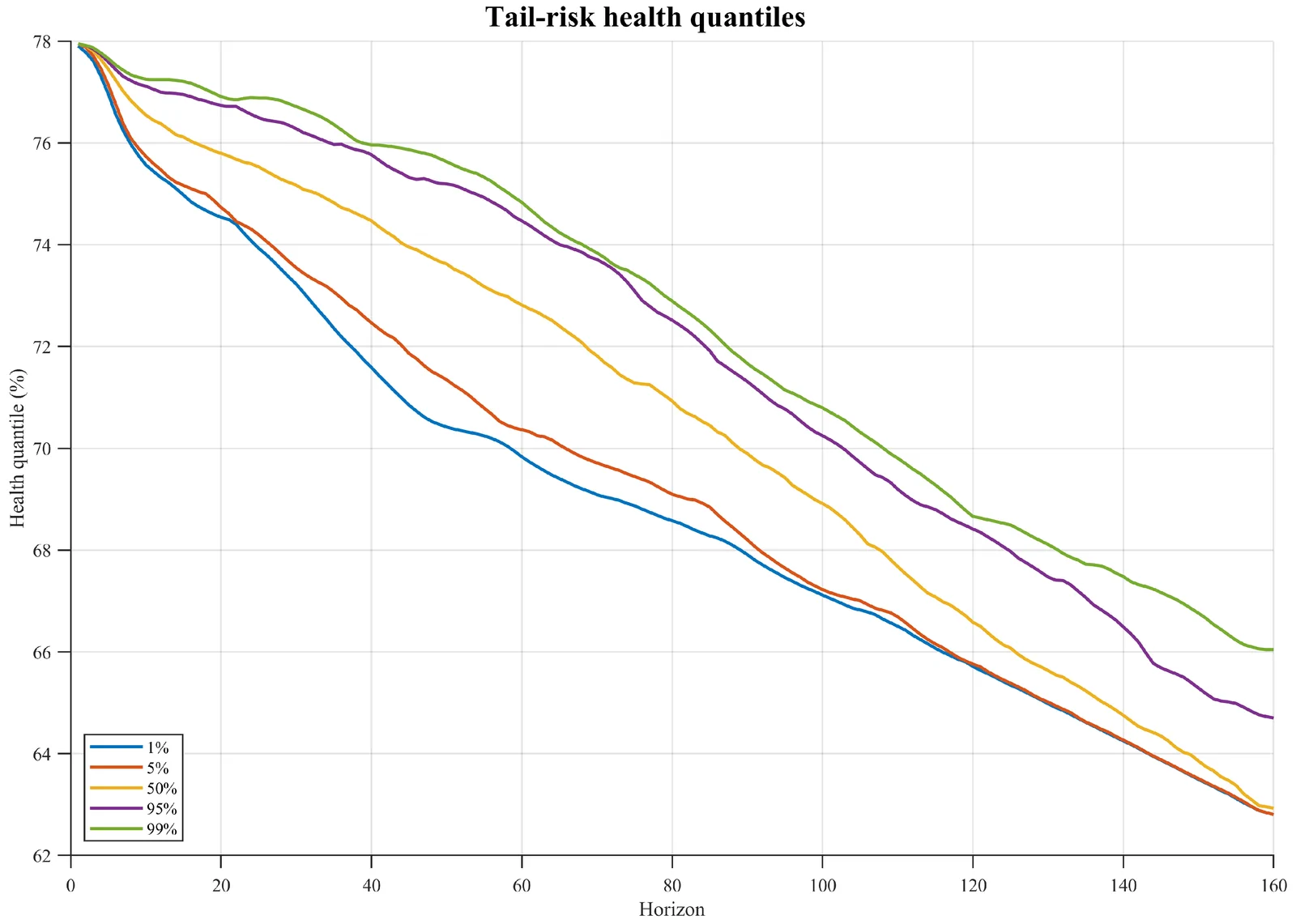
Tail-risk health quantiles derived directly from the accepted future ensemble.

Under L=78−H, the lower health tail in Figure 33 becomes the upper loss tail. VaR locates its threshold, and CVaR summarizes adverse severity. Rescreening common candidates separates gate-induced tail retention from changes in the generated trajectories or loss definition.

**Figure 34:**
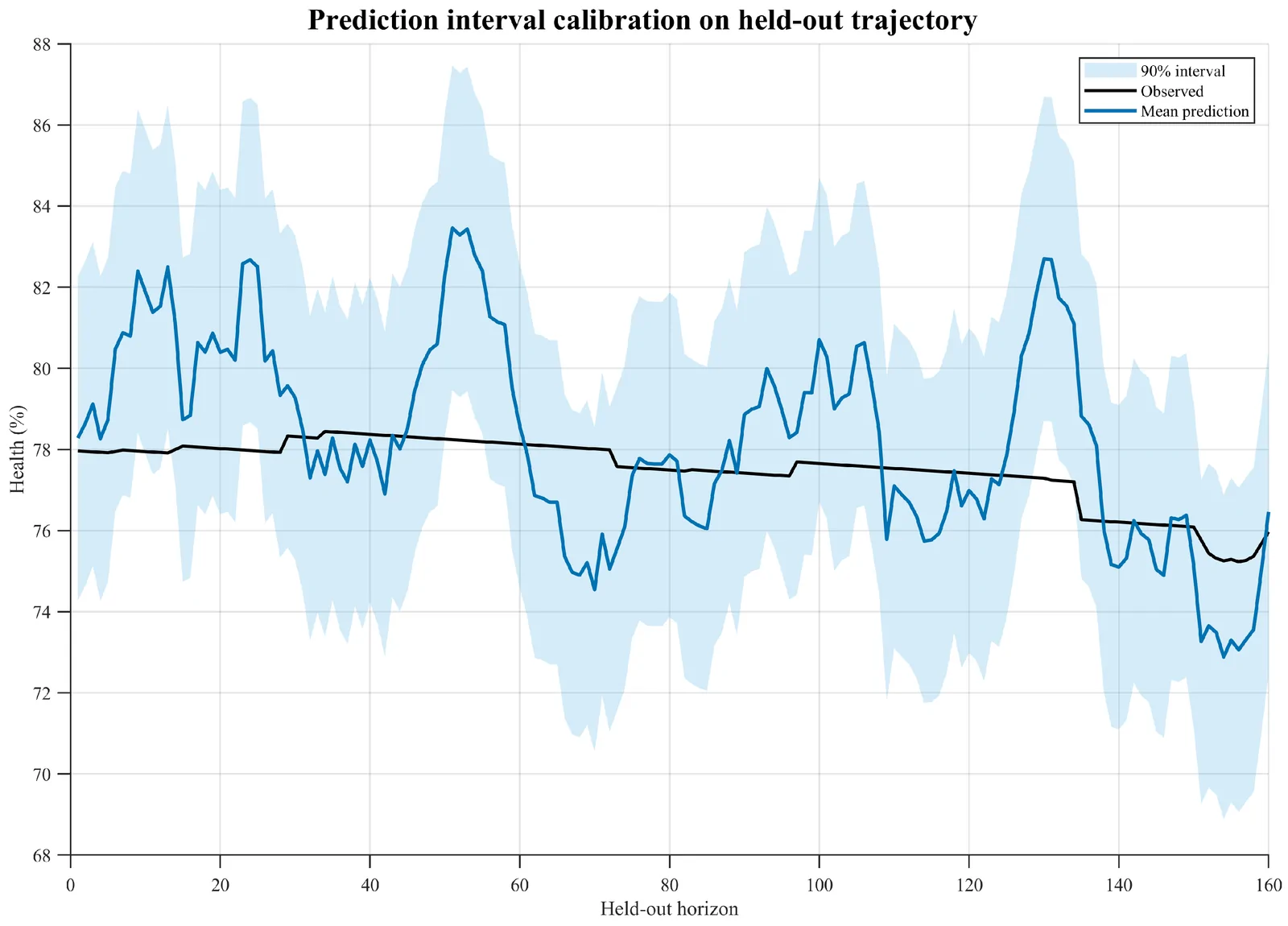
Health prediction, reference trajectory, and nominal 90% interval across forecast horizons.

Figure 34 illustrates interval location and spread; quantitative reference inclusion uses the full rolling-origin population. For scenario *f*, seed *s*, and forecast step *j*, let $q_{p,i,f,s}\left(j\right)$ denote the accepted-health empirical quantile. The fitted interval is $I_{i,f,s}\left(j\right)=\left[a_{s}q_{0.05,i,f,s}\left(j\right)+b_{s},\;a_{s}q_{0.95,i,f,s}\left(j\right)+b_{s}\right]$. Coverage and mean width are evaluated as

(21a) $$\begin{array}{cc} {\hat{C}}_{f,s}\left(j\right) & =\frac{100}{256}\sum_{i=1}^{256}⊮\;\left[H_{i,f}^{\mathrm{ref}}\left(j\right)\in I_{i,f,s}\left(j\right)\right],\\ {\hat{W}}_{f,s}\left(j\right) & =\frac{1}{256}\sum_{i=1}^{256}a_{s}\left[q_{0.95,i,f,s}\left(j\right)-q_{0.05,i,f,s}\left(j\right)\right]. \end{array}$$

Coverage is in percent and width is in health percentage points. Each scenario–seed run supplies 256 reference comparisons at each of 160 forecast steps, or 40,960 origin–horizon indicators. Across nine scenarios and five seeds, 11,520 predictive ensembles supply 1,843,200 such indicators. The 500 candidates forming each ensemble determine its quantiles; temporal dependence is carried by the origin histories and horizon sequences. The fixed initial state at j=0 is excluded from coverage and width summaries.

Horizon-specific scenario statistics average the five seed values. Table 3 then averages coverage equally over the nine scenarios and 160 forecast steps:

(21b) $$\bar{C}=\frac{1}{9\times 5\times 160}\sum_{f=1}^{9}\sum_{s=2026}^{2030}\sum_{j=1}^{160}{\hat{C}}_{f,s}\left(j\right).$$

The reported 90.00% is this rounded aggregate pointwise coverage. Scenario coverage ranges from 89.5% under 50% modality masking to 90.4% under sensor drift, as reported in Table A3. Coverage and width describe complementary properties: reference inclusion measures pointwise coverage of the constructed health path, while interval width measures the concentration of the accepted future distribution.

**Figure 35:**
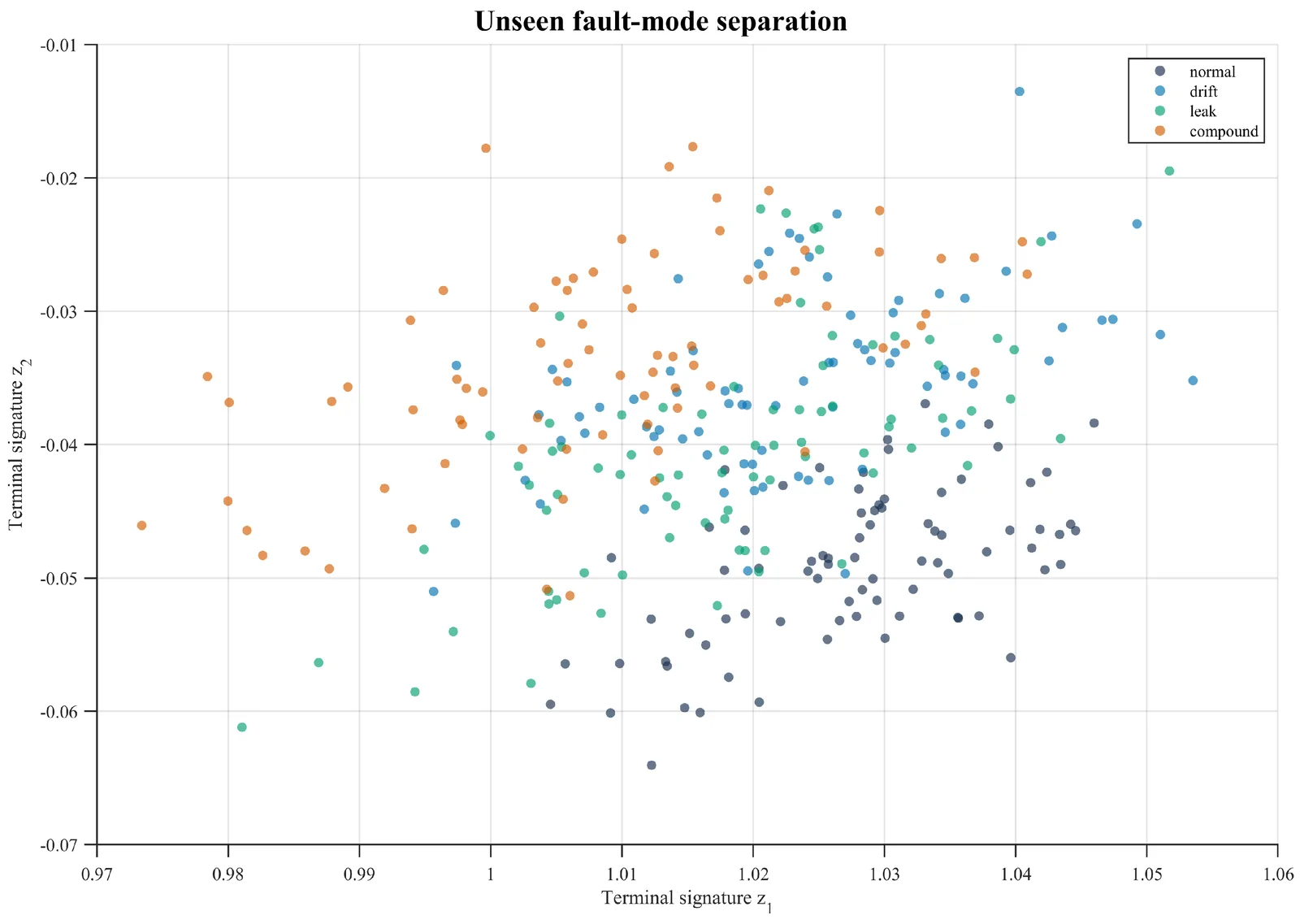
Terminal latent states under the prescribed normal, drift, leakage, and compound-fault scenarios.

Figure 35 contrasts normal, drift, leakage, and compound terminal populations. Leakage and compound interventions displace the controlled mechanical path; drift changes its inferred signature through observations. Their overlapping endpoints make trajectory history and event order relevant to mechanism interpretation.

**Figure 36:**
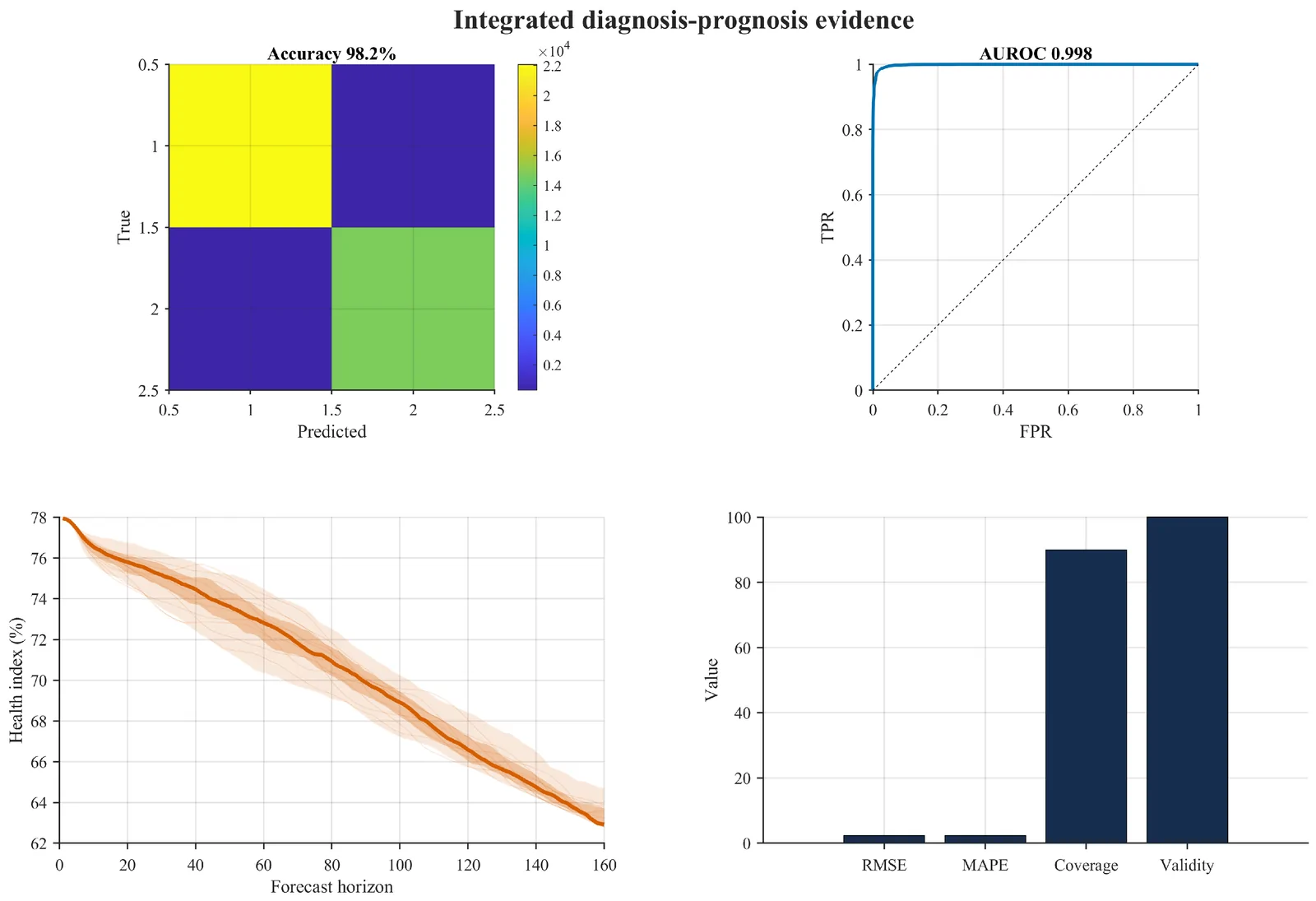
Integrated diagnosis and prognosis evidence combining discrimination, trajectory uncertainty, generation validity, and risk estimation.

Figure 36 links PHM2024 fault-label discrimination with MetroPT3 controlled trajectory reconstruction, interval reference inclusion, and candidate acceptance. Encoder and trajectory ablations retain these separate targets and supervision pathways.

Table 2a resolves mechanical/kinematic, thermal, atmospheric, and airspeed evidence in PHM2024. Pressure altitude and the derived density ratio supply environmental correction; the engine release supplies no pressure, flow, or current sensor for these configurations. P5 adds a torque–density–speed ratio, and P6 adds a gas-temperature residual and the NP/NG ratio at the diagnostic readout. Torque margin remains outside every diagnostic feature set and defines the reference-health readout in Equation (A4). P4 and Full use the same seven measurements and density ratio. P4 refits the head on the frozen Stage I encoder for 50 epochs; Full jointly optimizes the encoder and head for 50 Stage I epochs. This comparison changes diagnostic optimization with feature identity fixed. P5 and P6 evaluate additional derived descriptors.

Table 2b begins with measured compressor actuation and status. Oil temperature and H1 pressure enter at M2, the remaining pressure/flow channels at M3, and motor current at M4. These observations resolve mechanical loading through actuation, pressure build-up, flow, and electrical effort. M5 enables $\eta_{\mathrm{pc}}$ in health readout; M6 additionally enables control consistency and the valve–pressure residual. M6 uses deterministic SDA2D forecasting with gate evaluation. Full retains the same measured and derived evidence and uses conditional latent diffusion, intersection selection, and interval calibration. The separately trained deterministic-decoder row is the model substitution reported in Table 5. Thus, the M6–Full comparison resolves the forecast construction at fixed evidence. The efficiency and control-consistency terms enter Equations (A1)–(A3), and the valve–pressure residual supplies the physical gate in Equation (A12). The full-model gate value of 100.0% indicates admissibility within the accepted ensemble; candidate acceptance is 93.72% on the generated pool, with its scenario-level decomposition in Table A5.

P4 in Table 2 repeats the five-seed PHM2024 head-refitting statistic in Table 5, 93.75±0.18% macro-F1. The corresponding full-model joint fit gives 98.16±0.02%. Their 4.41-percentage-point difference contrasts joint representation–head optimization with head refitting on a frozen representation. This is a diagnostic training-protocol comparison; the independently parameterized MetroPT3 decoder, synthesis, selection, and calibration are assessed through the trajectory metrics.

Coverage precision is estimated by moving-block bootstrap over the time-ordered forecast origins, with circular indexing at the sequence boundary. The procedure uses 2000 replicates at seed 2026 and percentile confidence limits at 2.5% and 97.5%. Each resampled origin retains its complete 160-step sequence, all nine scenarios, and all five seeds; fitted models, gate thresholds, and calibration settings remain fixed. The main block length is 56 consecutive origins, using the ratio of the 224-block history–forecast support to the four-block origin stride. Coverage and interval width use the same evaluation population and equal weights. Table 8 resolves both quantities over four equally sized forecast ranges and nine scenarios.

The resampling unit preserves the dependence of forecast steps, scenarios, and seeds within each origin. The resulting confidence intervals describe temporal resampling variability of constructed-reference inclusion along the MetroPT3 chronology, conditional on the fitted models and prescribed scenario laws. Seed and scenario repetitions characterize conditional model behavior within that historical support.

Over the full 256-origin evaluation, coverage is 90.00% with a 95% confidence interval of 89.62–90.38% and mean width of 2.05 health percentage points. Averaging the four forecast-range widths or the nine scenario widths yields the same overall width. The horizon profile resolves early conservatism: coverage is 91.0% over 2–80 h and 89.4% over each of the two later ranges, while width increases from 1.30 to 2.75 percentage points. All nine scenario confidence intervals encompass the nominal rate. Resampling blocks of 28 and 112 origins under the same circular rule gives aggregate confidence intervals of 89.58–90.42% and 89.66–90.34%, respectively; the half-width changes by 0.04 percentage points relative to the 56-origin setting.

The source-window-disjoint check uses $O_{\mathrm{dis}}=\left\{908,1132,1356,1580,1804\right\}$, retaining the same nine scenarios, five seeds, and 160-step forecasts. For source-block origin *o*, let $I_{o,f,s}\left(j\right)$ denote the calibrated interval and $H_{o,f}^{\mathrm{ref}}\left(j\right)$ its constructed reference. The origin’s coverage and mean width are

$$\begin{array}{cc} {\bar{C}}_{o} & =\frac{100}{7200}\sum_{f=1}^{9}\sum_{s=2026}^{2030}\sum_{j=1}^{160}⊮\;\left[H_{o,f}^{\mathrm{ref}}\left(j\right)\in I_{o,f,s}\left(j\right)\right],\\ {\bar{W}}_{o} & =\frac{1}{7200}\sum_{f=1}^{9}\sum_{s=2026}^{2030}\sum_{j=1}^{160}a_{s}\;\left[q_{0.95,o,f,s}\left(j\right)-q_{0.05,o,f,s}\left(j\right)\right]. \end{array}$$

Panel (c) of Table 8 resolves the five origin means. Their 32,400 inclusions among 36,000 checks yield 90.00% coverage, with a mean width of 2.15 health percentage points, compared with 90.00% and 2.05 points over the complete rolling-origin population. Coverage across origins spans 89.50–90.50%, and mean width spans 2.08–2.22 points. The corresponding sample standard deviations are 0.354 and 0.054 percentage points, calculated across the five origin means with divisor 5−1. Across scenarios in the disjoint subset, coverage ranges from 89.3% to 90.6% and width from 1.90 to 2.40 points.

The historical support of this check comprises five windows from the same compressor chronology. Disjointness removes shared source indices while retaining common asset and temporal context. The reported ranges and sample standard deviations describe dispersion of the five origin means; the 36,000 inclusion indicators and their scenario–seed repetitions remain grouped within those origins. The confidence intervals in panels (a) and (b) of Table 8 concern the full 256-origin population. In both evaluations, coverage establishes pointwise inclusion of the constructed reference paths under the specified dynamics and interventions. Calibration against observed future degradation concerns a separate statistical target.

### 4.5. Evidence-Specific Parameter Sensitivity

Table 9 resolves the risk response to standard screening and to single-gate threshold perturbations on the same generated paths. The pool comprises 5,760,000 candidates from 256 origins, nine scenarios, and five seeds, with 500 proposals per origin. Counts are summed across these populations. Failure and fixed-tail probabilities normalize their retained event counts by the total retained count; RUL quantiles and terminal-loss VaR/CVaR use the corresponding pooled empirical distributions with equal path weights. Physical and dynamic thresholds vary individually by ±10% around (3.0,2.5,0), with model weights, candidate paths, the semantic threshold, and interval parameters fixed.

Standard screening retains 98.462% of failure-bearing paths and 93.722% of all candidates, increasing the pooled 320 h failure probability from 9.028% to 9.484%, a shift of 0.4565 percentage points. Relative to standard screening, physical-threshold changes of −10% and +10% shift failure probability by +0.0062 and +0.0017 percentage points; the corresponding dynamic changes give −0.0113 and +0.0077 points. These paired changes follow the event-retention ratio in Equation (19b). Candidate yield spans 93.10–94.19% under the perturbations. Fixed-tail retention tracks overall acceptance, and its probability is 0.05200 in all six populations at five-decimal precision. VaR and CVaR are 11.1 and 12.5 health percentage points across the six settings at the reported 0.1-point precision. Both pooled RUL quantiles exceed 320 h, consistent with cumulative failure mass below 0.10. The numerical comparison thus separates the screening-induced enrichment of first-passage events from the response to threshold perturbations and the terminal-loss tail.

Calibration acts on a different target through its weight $\lambda_{\mathrm{cal}}=0.1$; removal sets this weight to zero. Under the complete retraining protocol in Table 5, removing the calibration term gives 82.30±0.70% coverage, compared with 90.00±0.40% for the full configuration; trajectory RMSE changes from 2.310±0.05 to 2.250±0.05 percentage points. The 7.70-percentage-point coverage difference and 0.060-point RMSE difference distinguish distributional inclusion from forecast-center accuracy within this configuration comparison. The temporal dependence treatment is also stable to bootstrap-block choices of 28, 56, and 112 origins: the corresponding overall confidence intervals are [89.58,90.42], [89.62,90.38], and [89.66,90.34]%, with at most 0.04 percentage points of change in interval half-width. Together, these results support selecting parameters against the evidence channel they control while retaining a common final evaluation population.

## 5. Discussion

### 5.1. Mechanistic Contribution and Rare-Event Reasoning

Complete futures connect precursor timing, accumulated displacement, and adverse severity within one conditional law. Fault direction controls the prescribed mechanism, residual diffusion supplies path variation, and the three gates evaluate state compatibility, transition residuals, and event order. In MetroPT3, the deterministic reference replay identifies the shared construction against which learned reconstruction is assessed. PHM2024 independently evaluates present-state fault discrimination. Asset-specific projections and fitted parameters preserve these distinct sensing and supervision structures within the common eight-coordinate interface.

### 5.2. Scenario-Conditioned First-Passage and Tail Risk

Warning probability, censored first-passage RUL, prediction intervals, VaR, and CVaR are computed from the same accepted paths. Their joint interpretation depends on both the intervention law and screening: the retained failure mass and terminal-loss tail can respond differently to selection. The fixed-pool sensitivity analysis resolves these effects, while rejection signatures identify the physical, dynamic, or temporal criteria responsible for exclusion. Maintenance preserves pre-action failure history even when later health recovers.

### 5.3. Scalable World-Model Extension and Cross-Asset Deployment

Extension begins with an asset-specific mechanism-feature dictionary, health readout, and fitted $D_{a}\times 8$ projection. At least eight independent measured or label-free derived features support the orthogonal interface. Chronological transition fitting and physical predicates then follow the new asset. The reusable structure comprises the state format, conditioning schema, path-generation architecture, and risk functionals.

### 5.4. Limitations and Evidential Scope

MetroPT3 reference construction and prediction share fitted SDA2D operators, projection, health mapping, fault direction, time grid, and prescribed interventions. Trajectory error and coverage therefore quantify reconstruction and inclusion of constructed reference paths under this controlled law. Scaling, projections, health endpoints, fault directions, and physical predicates are fitted separately for each asset. The common interface preserves asset-specific sensor semantics. The source-window-disjoint check comprises five origins from one compressor chronology; repeated seeds and prescribed scenarios characterize training variation and conditional responses on these histories. Block-bootstrap uncertainty is conditional on that chronology and the fitted models. Prognosis has no validation against observed run-to-failure trajectories. RUL and tail loss characterize the scenario- and acceptance-conditioned ensemble, while measured PHM2024 fault labels independently support diagnosis.

## 6. Conclusions

Diffusion-RarePHM connects asset-specific health encoding, level–increment dynamics, conditional latent diffusion, and mechanism-directed interventions within a common path-based formulation. Physical, dynamic, and temporal-predicate gates define the accepted ensemble from which warning, censored RUL, interval, and tail-risk statistics are derived. This construction preserves the relation between degradation timing and adverse severity under explicit operating and maintenance conditions.

PHM2024 diagnosis achieves 98.23% accuracy, 98.16% macro-F1, and 0.9985 AUROC on the record-level held-out partition. Operating-condition separation gives 97.86±0.12% accuracy, 97.77±0.13% macro-F1, and 0.9979±0.0003 AUROC, with a 3.65-percentage-point accuracy gain over the PHM2024 encoder ablation. The MetroPT3-anchored controlled benchmark gives nine-scenario means of 2.250 health percentage-point RMSE, 2.294% MAPE, and 90.00% pointwise constructed-reference coverage. These results establish measured-label discrimination and controlled health-path reconstruction. The accepted ensemble unifies scenario generation, first-passage analysis, and tail-risk readout under the declared dynamics and intervention laws.

## Figures and Tables

**Table 1:** Dataset composition, evidential scope, and role in the Diffusion-RarePHM analytical framework.

Dataset	Asset/Regime	Rows Used	Principal Channels	Supervision	Role
PHM2024	Turbine-engine operating envelope	180,000 of 742,625	Torque, OAT, MGT, altitude, airspeed, NP, NG	Fault label; torque-margin health reference	Record- and condition-held-out diagnosis; health-reference analysis
MetroPT3	Rail compressor chronology	12,642 of 1,516,948	Pressures, oil temperature, motor current, binary controls, flow	Unlabeled trajectory	Normal dynamics, physical logic, and semi-physical features
Robot telemetry	Seven-axis arm and energy	Schema inspected	Speed, torque, angle, power, voltage, current	Normal and altered motion files	Extension constraint: action-energy compatibility
BatteryLife	Cross-source cell cycling benchmark	Schema and repository inspected	Voltage, current, capacity, temperature, chemistry, protocol	Life and cycling metadata	Extension constraint: chemistry-conditioned capacity direction

**Table 2:** Asset-specific evidence configurations. Panel (**a**): PHM2024 engine diagnosis. Each row lists its complete measured-variable set and derived quantities. Pressure altitude and the density ratio represent atmospheric operating conditions. The residual and ratio features are defined in Appendix C.1. P4 freezes the Stage I encoder and refits the diagnosis head for 50 epochs, repeating the five-seed statistics in Table 5 for seeds 2026–2030 and the common test partition. Full-model diagnosis jointly trains the encoder and head. Panel (**b**): MetroPT3 compressor prognosis. The set C contains compressor command (COMP), electric discharge-valve state, tower state, MPG, low-pressure switch, pressure switch, and oil level. H1 is a pressure channel. The derived quantities are pressure–current efficiency $\eta_{\mathrm{pc}}$, control consistency $f_{\mathrm{ctrl}}$, and valve–pressure residual $r_{v}$; they enter health readout and gate evaluation. M6 uses deterministic SDA2D propagation with gate assessment. Full uses conditional latent diffusion, the triple-gate intersection, and interval calibration. Forecast entries report means. Superscript *A* denotes retained-set validity for the full model; *C* denotes full-rule passage among all 128,000 candidates per scenario and seed for every other row, including the deterministic variant.

(a)					
Configuration	Measured Variables	Derived Quantities		Acc. (%)	Macro-F1 (%)
P1: mechanical/kinematic	torque, NP, NG	—		86.34	85.12
P2: add thermal	torque, NP, NG, OAT, MGT	—		88.12	87.01
P3: add altitude/density	torque, NP, NG, OAT, MGT, pressure altitude	$r_{\rho }$		90.34	89.34
P4: add airspeed; frozen Stage I encoder	torque, NP, NG, OAT, MGT, pressure altitude, IAS	$r_{\rho }$		94.01±0.15	93.75±0.18
P5: add performance ratio	torque, NP, NG, OAT, MGT, pressure altitude, IAS	$r_{\rho }$, $e_{\tau }$		93.45	92.56
P6: add cross-sensor residual/ratio	torque, NP, NG, OAT, MGT, pressure altitude, IAS	$r_{\rho }$, $e_{\tau }$, $r_{\mathrm{MT}}$, $r_{N}$		94.34	93.45
Full: joint encoder–head training	torque, NP, NG, OAT, MGT, pressure altitude, IAS	$r_{\rho }$		98.23	98.16±0.02
(**b**)					
**Configuration**	**Measured Variables**	**Derived Quantities**	**RMSE (p.p.)**	**MAPE (%)**	**Full-Rule Passage (%)**
M1: actuation/status	C	—	3.56	3.89	92.3 ^*C*^
M2: add oil temperature and H1 pressure	C, oil temperature, H1	—	3.21	3.56	93.8 ^*C*^
M3: add pressure/flow	C, oil temperature, H1, TP2, TP3, discharge-valve pressure, reservoir pressure, flow impulses	—	2.89	3.12	94.5 ^*C*^
M4: add current	C, oil temperature, H1, TP2, TP3, discharge-valve pressure, reservoir pressure, flow impulses, motor current	—	2.67	2.89	95.6 ^*C*^
M5: add efficiency	C, oil temperature, H1, TP2, TP3, discharge-valve pressure, reservoir pressure, flow impulses, motor current	$\eta_{\mathrm{pc}}$	2.56	2.67	96.8 ^*C*^
M6: consistency; deterministic SDA2D	C, oil temperature, H1, TP2, TP3, discharge-valve pressure, reservoir pressure, flow impulses, motor current	$\eta_{\mathrm{pc}}$, $f_{\mathrm{ctrl}}$, $r_{v}$	2.45	2.45	97.9 ^*C*^
Full: conditional latent diffusion	C, oil temperature, H1, TP2, TP3, discharge-valve pressure, reservoir pressure, flow impulses, motor current	$\eta_{\mathrm{pc}}$, $f_{\mathrm{ctrl}}$, $r_{v}$	2.250	2.294	100.0 ^*A*^
Full: deterministic decoder	C, oil temperature, H1, TP2, TP3, discharge-valve pressure, reservoir pressure, flow impulses, motor current	$\eta_{\mathrm{pc}}$, $f_{\mathrm{ctrl}}$, $r_{v}$	2.78	2.67	97.8 ^*C*^

**Table 3:** Primary quantitative results. Real diagnosis and controlled semi-physical prognosis are reported separately. Trajectory metrics are equal-weight averages over nine scenarios.

Metric	Value	Evidence Scope
Accuracy	98.23%	PHM2024 record-level held-out diagnosis
Macro-F1	98.16%	PHM2024 record-level held-out diagnosis
AUROC	0.9985	PHM2024 record-level held-out diagnosis
RMSE	2.250 percentage points	Nine-scenario mean; semi-physical health trajectory
MAPE	2.294%	Nine-scenario mean; semi-physical health trajectory
90% interval coverage	90.00%	Pointwise constructed-reference inclusion; nine-scenario mean over forecast steps 1–160
Candidate acceptance (three-gate intersection)	93.72%	Accepted/generated candidates; equal-weight mean over nine scenarios
Accepted-set admissibility	100.00%	Fraction satisfying all three criteria within the retained trajectory ensemble

**Table 4:** Author-computed performance across two asset-specific tasks. Diagnosis uses record-level PHM2024 adaptations on the common held-out partition; recurrent and Transformer diagnosis inputs have sequence length one. Prognosis uses chronological MetroPT3 histories and controlled semi-physical health paths. Performance entries average seeds 2026–2030; inference times are means ± sample standard deviations over 200 synchronized calls after 50 warm-up calls. Appendix C reports their latency distributions. Method configurations, adaptations, and task-specific computational profiles are defined in Appendix C.

	PHM2024: Turbine-Engine Diagnosis			MetroPT3: Compressor Prognosis			Computational Profile	
Method	Diagnosis Acc. (%)	Macro-F1 (%)	AUROC	RMSE (p.p.)	MAPE (%)	90% Coverage (%)	Time (ms) mean ± SD	Params (M)
CNN-1D (author)	84.34	83.12	0.89	4.56	4.98	82.3	8.2±0.3	3.6
LSTM [74]	85.56	84.34	0.90	4.23	4.67	83.1	10.8±0.4	6.1
GRU [75]	86.12	85.01	0.91	4.01	4.34	83.8	11.2±0.4	5.8
TCN [76]	85.89	84.67	0.90	4.12	4.45	83.4	7.5±0.3	4.3
Transformer [77]	88.45	87.34	0.93	3.78	3.98	85.6	25.3±0.8	20.7
Gated RNN (author variant) [75]	87.67	86.56	0.92	3.89	4.12	84.9	11.8±0.5	7.1
Dual-Attention (author variant) [78]	89.34	88.23	0.94	3.56	3.67	86.3	29.4±1.0	24.1
Bayesian dropout net [79]	89.89	88.78	0.94	3.34	3.45	87.1	19.8±0.7	15.8
GRL domain-adversarial [80]	90.12	89.01	0.95	3.23	3.34	87.8	22.4±0.8	18.2
Multi-Task PHM [81]	90.56	89.34	0.95	3.12	3.23	88.2	24.1±0.9	19.5
Physics-informed Bayesian [5]	91.23	90.12	0.96	2.98	3.01	88.9	33.6±1.2	29.1
LDT (adapted) [26]	91.89	90.78	0.96	2.89	2.89	89.2	35.8±1.3	32.4
Generative DG (adapted) [44]	92.34	91.23	0.97	2.78	2.78	89.7	38.1±1.4	34.8
Learned latent dynamics world model (author) [82]	92.89	91.78	0.97	2.71	2.71	90.1	40.5±1.5	37.6
Regularized conditional GAN [43,83]	93.45	92.34	0.98	2.65	2.65	90.4	42.8±1.6	40.2
Diffusion-RarePHM (Ours)	98.23	98.16	0.9985	2.250	2.294	90.00	46.2±1.1	44.8

**Table 5:** Ablations for PHM2024 diagnosis and MetroPT3 health-trajectory prediction. Performance entries are means over seeds 2026–2030; values following ± are sample standard deviations. PHM2024 uses the common 37,500-record test partition. Its full model jointly fits the encoder and head; the raw-fusion comparison changes its representation. The diagnosis readout accompanying the deterministic configuration is the frozen-encoder/head-refitting reference in Table A8. MetroPT3-only substitutions have no diagnosis cell. Their effects are assessed by the trajectory columns. Accepted counts are rounded means per scenario and seed. Gate superscript *A* denotes validity within retained paths; *C* denotes passage of the original full-model rules within the 128,000 generated candidates per scenario and seed. Dashes in diagnosis cells denote asset-inapplicable comparisons; dashes in the final column denote unreported full-rule audits for individual gate removals.

	PHM2024 Engine Diagnosis		MetroPT3 Compressor Prognosis				
Configuration	Diagnosis Acc. (%)	Macro-F1 (%)	RMSE (p.p.)	MAPE (%)	90% Coverage (%)	Mean Accepted	Full-Rule Passage (%)
Full Model	98.23	98.16±0.02	2.250±0.05	2.294±0.05	90.00±0.40	119,964	100.0 ± 0.0 ^*A*^
w/o Unified Health Encoder (raw fusion)	92.34±0.18	91.90±0.21	3.120±0.08	3.018±0.07	86.20±0.60	122,112	95.4 ^*C*^
w/o SDA2D Dynamics (single-level only)	—	—	2.890±0.07	2.780±0.06	87.10±0.55	123,008	96.1 ^*C*^
w/o Conditional Latent Diffusion (deterministic)	94.01±0.15	93.75±0.18	2.780±0.06	2.670±0.06	85.60±0.65	125,184	97.8 ^*C*^
w/o Fault-Direction Synthesis (random augmentation)	—	—	2.560±0.06	2.450±0.05	88.10±0.55	121,856	95.2 ^*C*^
w/o Physical Gate	—	—	2.450±0.05	2.380±0.05	88.90±0.50	120,768	–
w/o Dynamic Gate	—	—	2.410±0.05	2.340±0.05	89.20±0.45	121,652	–
w/o Semantic Gate	—	—	2.380±0.05	2.310±0.05	89.50±0.45	123,741	–
w/o Triple-Gate Intersection (weighted sum)	—	—	2.350±0.05	2.280±0.05	89.70±0.40	124,416	97.2 ^*C*^
w/o Calibration (raw intervals)	—	—	2.310±0.05	2.240±0.05	82.30±0.70	126,848	99.1 ^*C*^

**Table 6:** Model architecture, diffusion settings, and optimization configuration.

Component	Configuration
Latent trajectory	$d_{z}=8$; history 64×8; forecast 160×8; 2 h per forecast step
Asset input adapters	PHM2024: seven measurements plus derived density ratio, one-record input; MetroPT3: 15 channels, 64-record history; asset-fitted scaling and $D_{a}\times 8$ projection
Diffusion training	1,000 noise levels; linear $\beta_{\tau }$ from $10^{-4}$ to 0.02
Forecast sampling	100 DDIM updates; zero added reverse-step variance; 500 candidates per origin
Sampling indices	Zero-based 990,980,…,0; noise-array index *k* maps to level τ=k+1
History Transformer	Six layers; width 512; eight heads; feedforward width 2048
Denoising Transformer	Four layers; width 128; eight heads; feedforward width 512; GELU; dropout 0.1
Embeddings	Sinusoidal diffusion-time encoding with MLP; condition MLP 17→256→128; current SDA2D state projected 8→128 and added to condition embedding
Condition vector	17 entries: health 1; structure 4; duty 3; environment 3; maintenance 2; fault mode 4
SDA2D parameterization	Four-layer MLP parameterization for $A_{\psi },B_{\psi },b_{\psi }$; inputs $\left(z_{t},c_{t}\right)$; hidden width 512
PHM2024 diagnosis head	MLP 128→64→2; ReLU; dropout 0.1
PHM2024 diagnostic fits	50 epochs: joint encoder–head fit for the full model; frozen Stage I encoder and newly initialized head for the deterministic configuration
PHM2024 initialization	Encoder: Xavier-uniform weights and zero biases for the joint fit; Stage I weights retained for head refitting. Head: He-normal weights, zero biases, and dropout 0.1 in both fits
MetroPT3 stage budgets	50/20/100/30 epochs for Stages I–IV
Optimizer	Independent AdamW optimizers for the two branches; learning rate $2\times 10^{-4}$; weight decay $10^{-4}$; batch 128; cosine decay
Loss weights	MetroPT3: encoding/dynamics/diffusion/physics/semantics/calibration: 1/1/1/0.5/0.2/0.1; PHM2024: cross-entropy
Active MetroPT3 stage terms	I: encoding and dynamics; II: direction estimation; III: diffusion, physics, semantics; IV: physics, semantics, calibration
Regularization	Equation (7): $\lambda =10^{-4}$; Equation (14): $w_{r}=1$; Equation (21): β=0.1
MetroPT3 Stage IV	Encoder, SDA2D, and fault direction frozen; denoiser and calibration parameters updated; gate thresholds frozen before evaluation
Gate thresholds	Physical 3.0; dynamic 2.5; semantic 0; explicit temporal predicates, with no VLM call; trajectory scores as in Equation (A12)
Training relaxations	Gate-score sigmoid temperature $\tau_{u}=1.0$; score-level straight-through derivatives, with hard event-index extraction held constant; product-form soft coverage with ϑ=1.0 health percentage point; connected sampling and Gaussian rank-kernel quantiles with $h_{q}=0.02$ in quantile-level units
Interval calibration	$a_{s}=\mathrm{softplus}\left(\alpha_{s}\right)+10^{-6}$ and shift $b_{s}$ per seed; common 128 normal calibration origins; untruncated endpoints and hard membership at evaluation
Repeated realizations	Seeds 2026–2030; shared normal MetroPT3 fit and reference; independently trained diffusion modules and PHM2024 classifiers

**Table 7:** Robustness under sensor noise, modality dropout, and uncertainty interval quality. PHM2024 columns report diagnosis accuracy under standardized Gaussian noise. MetroPT3 Drop columns report the proportion of point forecasts within 2 health percentage points of the reference; width and coverage characterize the MetroPT3 intervals. Results are five-seed means and sample standard deviations. PHM2024 noise results compare the full classifier, raw-fusion representation, and frozen-encoder/head-refitting reference. Dashes denote MetroPT3-only substitutions, which do not enter the PHM2024 training graph.

	PHM2024 Engine Diagnosis		MetroPT3 Compressor Prognosis			
Configuration	Noise σ = 0.1 Acc. (%)	Noise σ = 0.3 Acc. (%)	Drop 20% Within 2 p.p. (%)	Drop 50% Within 2 p.p. (%)	Avg. Interval Width (p.p., σ = 0.3)	Coverage (%) (σ = 0.3)
Full Model	97.12±0.10	94.34±0.15	94.5±0.2	89.2±0.3	2.12±0.04	92.78±0.35
w/o Unified Health Encoder	93.12±0.16	88.34±0.20	91.2±0.3	85.6±0.4	2.89±0.06	87.34±0.55
w/o SDA2D Dynamics	—	—	92.1±0.3	86.3±0.4	2.67±0.05	88.89±0.50
w/o Conditional Latent Diffusion	94.12±0.14	89.78±0.18	92.8±0.3	87.0±0.4	2.56±0.05	89.45±0.45
w/o Fault-Direction Synthesis	—	—	93.0±0.3	87.6±0.4	2.45±0.04	90.12±0.40
w/o Triple-Gate Validator	—	—	92.1±0.3	86.1±0.4	2.78±0.05	88.12±0.50
w/o Calibration (raw intervals)	—	—	93.5±0.3	88.1±0.4	3.45±0.07	83.56±0.65

**Table 8:** Pointwise coverage of constructed reference paths and mean width of the nominal 90% health interval. Panel (a) averages nine scenarios and five seeds within each forecast range; panel (b) averages all 160 forecast steps and five seeds within each scenario. Both use 256 origins, with confidence intervals from 2000 circular block-bootstrap replicates of 56-origin blocks. Panel (c) reports each of the five source-window-disjoint origins, aggregating nine scenarios, five seeds, and 160 steps into 7200 reference-inclusion checks per origin. Width is in health percentage points.

Forecast Range/Scenario	Coverage (%)	95% CI (%)	Width (p.p.)
*(a) Forecast range*			
Steps 1–40 (2–80 h)	91.0	[90.4, 91.6]	1.30
Steps 41–80 (82–160 h)	90.2	[89.6, 90.8]	1.80
Steps 81–120 (162–240 h)	89.4	[88.8, 90.0]	2.35
Steps 121–160 (242–320 h)	89.4	[88.7, 90.1]	2.75
*(b) Scenario*			
Normal	90.2	[89.8, 90.6]	1.85
Early fault	89.9	[89.4, 90.4]	2.00
Slow leakage	90.1	[89.6, 90.6]	2.10
Compound fault	89.8	[89.2, 90.4]	2.30
Maintenance	90.3	[89.9, 90.7]	1.95
Thermal-load stress	90.0	[89.5, 90.5]	2.15
Sensor drift	90.4	[89.9, 90.9]	1.90
Missing 20%	89.8	[89.2, 90.4]	2.05
Missing 50%	89.5	[88.9, 90.1]	2.15
Overall equal-weight mean	90.00	[89.62, 90.38]	2.05
*(c) Source-window-disjoint origins*			
**Origin**	**Hits/checks**	**Coverage (%)**	**Width (p.p.)**
908	6444/7200	89.50	2.08
1132	6480/7200	90.00	2.18
1356	6516/7200	90.50	2.12
1580	6480/7200	90.00	2.22
1804	6480/7200	90.00	2.15
Five-origin aggregate	32,400/36,000	90.00	2.15

**Table 9:** Pooled risk sensitivity before and after screening on the same 5,760,000 candidates. Panel (a) reports retained counts, acceptance yield, and event-specific retention; panel (b) reports 320 h first-passage probability, RUL quantiles, and risk of terminal health loss $L_{160}=78-H_{160}$. The semantic threshold is zero for every screened setting.

(a) Candidate and Event Retention								
Setting	$\kappa_{\mathrm{phys}}$	$\kappa_{\mathrm{dyn}}$	*K*	$\hat{a}$	$n_{f}$	${\hat{r}}_{f}$	$n_{0.95}$	${\hat{r}}_{0.95}$
Unscreened	–	–	5,760,000	1.00000	520,000	1.00000	299,520	1.00000
Standard	3.0	2.5	5,398,400	0.93722	512,000	0.98462	280,718	0.93723
Physical −10%	2.7	2.5	5,375,900	0.93332	510,200	0.98115	279,548	0.93332
Physical +10%	3.3	2.5	5,418,720	0.94075	514,020	0.98850	281,773	0.94075
Dynamic −10%	3.0	2.25	5,362,400	0.93097	507,980	0.97688	278,844	0.93097
Dynamic +10%	3.0	2.75	5,425,400	0.94191	514,980	0.99035	282,119	0.94190
**(b) First-passage and terminal-loss risk**								
Setting		$P_{f}\left(320\;h\right)$		RUL $q_{0.10}$ (h)	RUL $q_{0.50}$ (h)	${\mathrm{VaR}}_{0.95}$	${\mathrm{CVaR}}_{0.95}$	$\hat{Q}\left(E_{0.95}\right)$
Unscreened		0.09028		>320	>320	11.1	12.5	0.05200
Standard		0.09484		>320	>320	11.1	12.5	0.05200
Physical −10%		0.09491		>320	>320	11.1	12.5	0.05200
Physical +10%		0.09486		>320	>320	11.1	12.5	0.05200
Dynamic −10%		0.09473		>320	>320	11.1	12.5	0.05200
Dynamic +10%		0.09492		>320	>320	11.1	12.5	0.05200

*K* is the retained count, $\hat{a}=K/5,760,000$, and $n_{f}$ and $n_{0.95}$ count retained failure and fixed-tail events. Their retentions are ${\hat{r}}_{f}=n_{f}/520,000$ and ${\hat{r}}_{0.95}=n_{0.95}/299,520$. The fixed tail uses each origin’s unfiltered 95th-percentile terminal-loss threshold and includes the quantile point. At 500 candidates, this is the 475th order statistic; the reported inclusive tail contains 26 paths per pool, totaling 299,520 (5.2%). The fixed-tail probability is $n_{0.95}/K$. RUL entries >320 denote right censoring; VaR/CVaR are in health percentage points and reported to 0.1 point.

## Data Availability

Public measurement sources and sampling protocols are identified in Section 2 and Appendix A and Appendix B. The companion reproducibility archive provides executable audit and calibration-reference routines, the index-derived MetroPT3 origin manifest, and author-reported audit summaries. The utilities construct source windows, validate exported PHM2024 split identities, aggregate per-origin error and interval statistics, and capture synchronized inference timings from a supplied model callable. Appendix D specifies their inputs and reporting units.

## Appendix A. Trajectory Benchmark Definition and Construction

### Appendix A.1. Health Definition and Endpoint Estimation

Let C(a)=min{1,max{0,a}}. Each component equals one at its normal-reference endpoint and zero at its adverse endpoint. For MetroPT3, the thermal, pressure–current, and latent components are

(A1) $$\begin{array}{cc} f_{\mathrm{oil}} & =C\;\left(\frac{T_{\mathrm{oil},\mathrm{crit}}-T_{\mathrm{oil}}}{T_{\mathrm{oil},\mathrm{crit}}-T_{\mathrm{oil},\mathrm{ref}}}\right),\\ \eta_{\mathrm{pc}} & =\frac{TP2}{\mathrm{motor}\_\mathrm{current}+\epsilon },\;f_{\mathrm{pc}}=C\;\left(\frac{\eta_{\mathrm{pc}}-\eta_{\mathrm{pc},\mathrm{crit}}}{\eta_{\mathrm{pc},\mathrm{ref}}-\eta_{\mathrm{pc},\mathrm{crit}}}\right),\\ f_{\mathrm{lat}} & =C\;\left(\frac{z_{1}-z_{1,\mathrm{crit}}}{z_{1,\mathrm{ref}}-z_{1,\mathrm{crit}}}\right). \end{array}$$

Here $T_{\mathrm{oil},\mathrm{crit}}>T_{\mathrm{oil},\mathrm{ref}}$, $\eta_{\mathrm{pc},\mathrm{ref}}>\eta_{\mathrm{pc},\mathrm{crit}}$, and $z_{1,\mathrm{ref}}>z_{1,\mathrm{crit}}$; $\epsilon =10^{-8}$ safeguards the current denominator. Control consistency is the complement of a windowed mismatch rate:

(A2) $$f_{\mathrm{ctrl}}\left(t\right)=1-\frac{1}{W_{c}}\sum_{\tau =t-W_{c}+1}^{t}⊮\;\left[{\mathrm{COMP}}_{\tau }\neq {\mathrm{COMP}}_{\mathrm{expected}}\left(\tau \right)\right],\;W_{c}=10.$$

Here *t* and τ index two-hour trajectory blocks. The ten-block window spans 20 h, corresponding to 60 retained 20 min observations. The expected compressor state follows a hysteretic rule:

(A3) $${\mathrm{COMP}}_{\mathrm{expected}}\left(t\right)=\left\{\begin{array}{cc} 1, & TP2\left(t\right)>\tau_{\mathrm{load}}\;\mathrm{and}\;H1\left(t\right)>\tau_{H1},\\ 0, & TP2\left(t\right)<\tau_{\mathrm{unload}},\\ \mathrm{COMP}(t-1), & \mathrm{otherwise}. \end{array}\right.$$

The normal-fitting thresholds are $\tau_{\mathrm{load}}=8.5$ bar, $\tau_{\mathrm{unload}}=7.5$ bar, and $\tau_{H1}=0.5$ bar, and remain fixed for subsequent scenarios.

For PHM2024, torque margin and measured gas temperature contribute through

(A4) $$f_{\mathrm{TM}}=C\;\left(\frac{TM-TM_{\mathrm{crit}}}{TM_{\mathrm{ref}}-TM_{\mathrm{crit}}}\right),\;f_{\mathrm{MGT}}=C\;\left(\frac{MGT_{\mathrm{crit}}-MGT}{MGT_{\mathrm{crit}}-MGT_{\mathrm{ref}}}\right),$$

with $TM_{\mathrm{ref}}>TM_{\mathrm{crit}}$ and $MGT_{\mathrm{crit}}>MGT_{\mathrm{ref}}$. The latent component has the form in Equation (A1), with asset-specific endpoints. The two weighted scores and percentage-scale health readouts are

(A5) $$\begin{array}{cc} d_{M} & =0.25f_{\mathrm{oil}}+0.30f_{\mathrm{pc}}+0.15f_{\mathrm{ctrl}}+0.30f_{\mathrm{lat}},\\ d_{\mathrm{PHM}} & =0.55f_{\mathrm{TM}}+0.25f_{\mathrm{MGT}}+0.20f_{\mathrm{lat}},\\ H_{a} & =H_{\min}+\left(H_{\mathrm{norm}}-H_{\min}\right)d_{a}=60+18d_{a},\;a\in \left\{M,\mathrm{PHM}\right\}. \end{array}$$

Thus, $H_{\min}=60$, $H_{\mathrm{norm}}=78$, and $H_{a}\in \left[60,78\right]$. Empirical health quantiles are calculated after this mapping and satisfy $60\leq H_{q05}\leq H_{q50}\leq H_{q95}\leq 78$. The warning boundary $H_{\mathrm{warn}}=74$ and first-passage boundary $H_{\mathrm{crit}}=65$ define distinct events without altering the health readout. Table A1 gives the numerical endpoints, estimated from normal MetroPT3 fitting blocks 0–421 and normal PHM2024 development observations. The component subscript “crit” denotes its adverse normalization endpoint, while $H_{\mathrm{crit}}$ denotes the health-level first-passage boundary.

**Table A1:** Health-component endpoints and their normal-fitting statistics.

Asset	Component Variable	Reference Endpoint	Adverse Endpoint
MetroPT3	Oil temperature (°C)	50.0 (median)	65.0 (95th percentile)
MetroPT3	Pressure–current efficiency (bar/A)	2.00 (median)	1.00 (5th percentile)
MetroPT3	Oriented $z_{1}$ (dimensionless)	1.05 (median)	0.72 (5th percentile)
PHM2024	Torque margin (%)	35.0 (median)	5.0 (5th percentile)
PHM2024	Measured gas temperature (°C)	620.0 (median)	700.0 (95th percentile)
PHM2024	Oriented $z_{1}$ (dimensionless)	1.00 (median)	0.60 (5th percentile)

Future latent states are read in physical units by restoring the robust channel scales used in Equation (4):

(A6) $$\begin{array}{cc} \hat{x} & =\mathrm{med}+SWz,\;S={\mathrm{diag}}_{r=1}^{D}\left(1.4826\;{\mathrm{MAD}}_{r}+\epsilon_{s}\right),\\ W & \in R^{D\times d_{z}},\;W^{T}W=I_{d_{z}},\;d_{z}=8,\epsilon_{s}=10^{-8}. \end{array}$$

This is the orthogonal-projection reconstruction of the standardized feature vector. MetroPT3 uses D=15. Binary control channels are recovered as ${\hat{b}}_{r}=⊮\left[{\hat{x}}_{r}\geq \tau_{b,r}\right]$, where $\tau_{b,r}$ is the midpoint between the fitted zero and one classes in normal blocks 0–421. The reconstructed continuous variables, thresholded controls, and oriented latent coordinate enter the component maps above; component clipping precedes their weighted sum.

### Appendix A.2. Intervention Laws and Parameter Values

The local forecast index is j=0,…,160, with one step corresponding to two hours. Define $u_{j_{0}}\left(j\right)=\max\left(j-j_{0},0\right)$ and the saturating displacement

(A7) $$R\left(j;A,j_{0},\tau \right)=A\left[1-\exp\;\left(-\frac{u_{j_{0}}\left(j\right)}{\tau }\right)\right].$$

The normal latent reference $z_{n}\left(j\right)$ is completed before intervention. Fault references are formed as $z_{f}^{\mathrm{ref}}\left(j\right)=z_{n}\left(j\right)+\rho_{f}\left(j\right)v_{f}$, with $v_{f}$ defined by Equation (12). The profile specifies the total displacement at time *j*; an incremental implementation uses $\rho_{f}\left(j\right)-\rho_{f}\left(j-1\right)$. The profiles and finite-horizon ranges are given in Table A2.

The fitted direction used for the reported interventions is stored as

(A8) $$\begin{array}{cc} u_{f} & ={\left(-0.943,\;0.277,\;-0.164,\;0.072,\;0.041,\;-0.031,\;0.010,\;0.000\right)}^{T},\\ v_{f} & =u_{f}/{∥u_{f}∥}_{2},\;{∥u_{f}∥}_{2}≃1.000400. \end{array}$$

Normalization is applied before rollout, so each $\rho_{f}\left(j\right)$ measures displacement along a unit direction.

The thermal-load intervention modifies the mean transition under the stress condition while retaining the innovation covariance:

(A9) $$f_{\psi ,\mathrm{stress}}\left(z,\Delta z,c\right)=1.8A_{\psi }\left(z,c\right)z+1.3B_{\psi }\left(z,c\right)\Delta z+1.2b_{\psi }\left(z,c\right).$$

Maintenance follows a linear pre-action profile $\rho_{\mathrm{pre}}\left(j\right)=0.35j/95$. Step $j_{m}=95$ records the state immediately before maintenance. The action at $95^{+}$ removes a fraction $R_{m}=0.55$ of the displacement, leaving $R_{\mathrm{res}}=0.45$ to decay with $\tau_{\mathrm{rec}}=25$. With no additional post-maintenance damage term, the reference is

(A10) $$z_{m}^{\mathrm{ref}}\left(j\right)=\left\{\begin{array}{cc} z_{n}\left(j\right)+0.35\left(j/95\right)v_{f}, & 0\leq j\leq 95,\\ z_{n}\left(j\right)+0.1575e^{-(j-95)/25}v_{f}, & 95<j\leq 160. \end{array}\right.$$

The instantaneous action removes 55% of the pre-maintenance displacement. The remaining displacement decays during the recovery transition. This construction preserves incomplete immediate restoration and a finite recovery timescale without repeated repair increments.

Drift and missingness alter the model’s observation stream. Their mechanical reference is the uncorrupted normal path under the same initial state and condition, so normal, drift, and missing-sensor comparisons share an identical reference health trajectory. Their predictive means and intervals can differ through observation-conditioned inference.

For MetroPT3, the mask acts on $C_{\mathrm{miss}}=\left\{TP2,TP3,H1,\mathrm{motor}\_\mathrm{current}\right\}$, with all remaining channels observed. Missingness is the fraction of unavailable observation cells within these four channels. The 20% and 50% settings therefore correspond to 5.33% and 13.33% of the full 15-channel input, respectively. Contiguous runs span 2–8 two-hour blocks (4–16 h), with positions selected using seed 2026. Neither mask changes the mechanical reference or rescales $\Sigma_{\eta }$.

**Table A2:** Fault and observation interventions over the 160-step forecast. Time constants and onset indices are in forecast steps.

Scenario	Intervention Law	Parameters and Range
Normal	$\rho_{n}\left(j\right)=0$	No mechanical intervention
Early fault	$\rho_{e}\left(j\right)=R\left(j;0.35,55,18\right)$	$0\leq \rho_{e}\left(j\right)\leq \rho_{e}\left(160\right)$; $\rho_{e}\left(160\right)≃0.348975$
Slow leakage	$\rho_{l}\left(j\right)=R\left(j;0.30,15,45\right)+0.25{\left[u_{15}\left(j\right)/160\right]}^{2}$	$0\leq \rho_{l}\left(j\right)\leq \rho_{l}\left(160\right)$; $\rho_{l}\left(160\right)≃0.493362$
Compound fault	$\rho_{c}\left(j\right)=R\left(j;0.30,20,25\right)+R\left(j;0.45,80,12\right)$	$0\leq \rho_{c}\left(j\right)\leq \rho_{c}\left(160\right)$; $\rho_{c}\left(160\right)≃0.748318$
Sensor drift	$x_{d}^{\mathrm{obs}}\left(j\right)=x^{\mathrm{obs}}\left(j\right)+a\left[1-e^{-j/\tau_{d}}\right]e_{TP2}$	a=0.12 bar; $\tau_{d}=20$
Missing sensors	$x_{m}^{\mathrm{obs}}\left(j\right)=x^{\mathrm{obs}}\left(j\right)⊙m\left(j\right)$	20% and 50% within $C_{\mathrm{miss}}$; contiguous lengths 2–8 blocks; seed 2026
Thermal-load stress	Conditional mean transition in Equation (A9)	Multipliers 1.8, 1.3, and 1.2 for level, increment, and drift terms; common $\Sigma_{\eta }$

### Appendix A.3. Reference Construction, Deterministic Comparator, and Evaluation

MetroPT3 blocks 0–421 (422 blocks) determine normal fitting, blocks 422–843 (422 blocks) supply calibration, and blocks 844–2106 (1263 blocks) form the evaluation segment. On the nominal two-hour benchmark grid starting at 00:00 on 1 February 2020, these intervals extend to 02:00 on 7 March, from 04:00 on 7 March to 06:00 on 11 April, and from 08:00 on 11 April to 12:00 on 25 July, respectively. Continuous channels are aggregated by their six-sample mean; binary controls use their final retained value. The 256 forecast origins are $t_{i}=908+4i$, i=0,…,255, so the actual selected indices run from 908 to 1928. Each origin has history $\left[t_{i}-63,t_{i}\right]$ and future $\left[t_{i}+1,t_{i}+160\right]$, both within the evaluation segment. The final forecast ends at block 2088. Normal validation residuals determine innovation covariance and gate thresholds. At an origin $t_{i}$, the reference construction is as follows:

1. Infer $z_{i,0}$ from the measured history and obtain $\Delta z_{i,0}$ from the final two latent states. Fix the prospective condition $c_{i}$.
2. Roll out the deterministic normal path using $z_{i,n}\left(j+1\right)=f_{\psi }\left(z_{i,n}\left(j\right),\Delta z_{i,n}\left(j\right),c_{i}\right)$ and $\Delta z_{i,n}\left(j\right)=z_{i,n}\left(j\right)-z_{i,n}\left(j-1\right)$.
3. Apply the prescribed fault displacement or single maintenance action to the completed normal path. For observational drift and missingness, retain the normal mechanical path and apply the perturbation to the observations supplied to prediction.
4. Restore the physical channel scales using Equation (A6), threshold the reconstructed binary controls, and evaluate the clipped health components on the uncorrupted reference. The resulting $H_{i,f}^{\mathrm{ref}}\left(j\right)$ is the benchmark ground truth for that origin and scenario.
5. Generate 500 candidate futures from the same origin and condition for each seed. Apply all three gates and compute the predictive mean, 5th percentile, and 95th percentile from the accepted health paths.

The reference and predictive mean transitions use the same fitted SDA2D parameters, estimated from normal blocks 0–421. They also share robust-standardization parameters, the orthogonal projection matrix, normalized fault direction, health endpoints, time grid, and prescribed intervention profile. Reference construction contains no stochastic innovation, reverse-diffusion sampling, gate selection, or calibration adjustment. These shared fitted operators and mechanism rules define the mechanical scenario against which conditional generation and interval reference inclusion are assessed. Accordingly, the trajectory metrics quantify agreement with a controlled semi-physical reference; the PHM2024 held-out diagnosis results assess discrimination against measured fault labels.

During reference mean propagation, the latent state is bounded using the normal-fitting percentile support before inverse projection and health readout. This deterministic state-bound operation is distinct from triple-gate screening. The clipped component maps and convex weights yield health in [60,78] directly, without an additional health-level projection.

The deterministic shared-operator comparator explicitly replays the reference-generating chain from the uncorrupted reference-side initial state. Let B denote the fitted latent-state bound, $f_{\psi ,f}$ the normal SDA2D transition or the prescribed thermal-load transition, $I_{f}$ the scenario intervention on a completed path, and $D_{H}$ the inverse projection and component-wise health readout. Its rollout is

(A10a) $$\begin{array}{cc} z_{i,n}^{\mathrm{SO}}\left(0\right) & =z_{i,0},\;\Delta z_{i,n}^{\mathrm{SO}}\left(0\right)=\Delta z_{i,0},\\ z_{i,n}^{\mathrm{SO}}\left(j+1\right) & =B\;\left[f_{\psi ,f}\;\left(z_{i,n}^{\mathrm{SO}}\left(j\right),\Delta z_{i,n}^{\mathrm{SO}}\left(j\right),c_{i}\right)\right],\\ {\hat{H}}_{i,f}^{\mathrm{SO}}\left(j\right) & =D_{H}\;\left[I_{f}\;\left(z_{i,n}^{\mathrm{SO}}\right)\right]\left(j\right)=H_{i,f}^{\mathrm{ref}}\left(j\right). \end{array}$$

Fault displacement and maintenance use the exact profiles in Appendix A; for drift and masking, $I_{f}$ leaves the mechanical reference unchanged. Equality with $H^{\mathrm{ref}}$ gives ${\mathrm{RMSE}}_{\mathrm{SO}}={\mathrm{MAPE}}_{\mathrm{SO}}=0$ at every origin and scenario. These zeros are algebraic consequences of reference replay, rather than empirical scores from a separately trained predictor. The comparator identifies the construction against which learned reconstruction is assessed. The learned latent dynamics world model in Table 4 uses its task-trained prediction branch and residual-calibrated intervals; the deterministic decoder ablation in Table 5 also undergoes retraining. Neither comparator is defined by exact replay of the shared reference operators.

Let $r_{t}=z_{t+1}-f_{\psi }\left(z_{t},\Delta z_{t},c_{t}\right)$ be a normal-validation residual and $\Sigma_{0}$ its sample covariance. Radius truncation is defined by

(A11) $${\tilde{r}}_{t}=r_{t}\min\left\{1,\sqrt{\frac{\chi_{0.95,d_{z}}^{2}}{r_{t}^{T}\Sigma_{0}^{-1}r_{t}}}\right\},\;\Sigma_{\eta }=\mathrm{Cov}\left({\tilde{r}}_{t}\right),$$

with the multiplier set to one for a zero residual and $d_{z}$ denoting the latent dimension. All primary scenarios use this same covariance and fixed gate thresholds. Observation-noise robustness tests perturb the measurements externally.

Gate decisions aggregate pointwise violations over the complete future. With $Q_{0.95,j}$ denoting the empirical 95th percentile over j=1,…,160,

(A12) $$\begin{array}{cc} g_{\mathrm{phys}}\left(\hat{Z}\right) & =Q_{0.95,j}\;\left[\frac{|{\hat{DV}}_{\mathrm{pressure}}\left(j\right)-f_{\mathrm{phys}}\left(\hat{TP2}\left(j\right),\hat{TP3}\left(j\right)\right)|}{\sigma_{\mathrm{phys}}}\right],\\ e_{j} & ={\hat{z}}_{j}-f_{\psi }\left({\hat{z}}_{j-1},\Delta {\hat{z}}_{j-1},c\right),\\ g_{\mathrm{dyn}}\left(\hat{Z}\right) & =Q_{0.95,j}\;\left[\sqrt{e_{j}^{T}\Sigma_{\eta }^{-1}e_{j}}\right],\\ g_{\mathrm{sem}}\left(\hat{Z}\right) & =\sum_{j=1}^{160}⊮\left[\mathrm{event}-\mathrm{order}\;\mathrm{violation}\;\mathrm{at}\;j\right]. \end{array}$$

The fixed thresholds are $\kappa_{\mathrm{phys}}=3.0$, $\kappa_{\mathrm{dyn}}=2.5$, and $\kappa_{\mathrm{sem}}=0$, with acceptance given by Equation (15). The dynamic residual retains both the latent increment and the prospective condition used by the SDA2D rollout. These trajectory-level gates are applied to candidates, while reference generation uses the deterministic mechanism construction above.

For a fixed initial state and deterministic health readout, every candidate has the same health at j=0; hence its mean and both quantiles coincide with the reference. Forecast statistics and errors are evaluated over j=1,…,160. For scenario *f* and seed *s*, with N=256 origins and accepted-ensemble mean ${\bar{H}}_{i,f,s}\left(j\right)$,

(A13) $${\mathrm{RMSE}}_{f,s}=\sqrt{\frac{1}{160N}\sum_{i=1}^{N}\sum_{j=1}^{160}{\left[{\bar{H}}_{i,f,s}\left(j\right)-H_{i,f}^{\mathrm{ref}}\left(j\right)\right]}^{2}}.$$

MAPE and interval coverage use the same origin–step pooling within each seed:

(A14) $$\begin{array}{cc} {\mathrm{MAPE}}_{f,s} & =\frac{100}{160N}\sum_{i=1}^{N}\sum_{j=1}^{160}\frac{|{\bar{H}}_{i,f,s}\left(j\right)-H_{i,f}^{\mathrm{ref}}\left(j\right)|}{H_{i,f}^{\mathrm{ref}}\left(j\right)},\\ {\mathrm{Coverage}}_{f,s} & =\frac{100}{160N}\sum_{i=1}^{N}\sum_{j=1}^{160}⊮\;\left[H_{q05,i,f,s}\left(j\right)\leq H_{i,f}^{\mathrm{ref}}\left(j\right)\leq H_{q95,i,f,s}\left(j\right)\right]. \end{array}$$

For any reported metric *m*, the scenario value is $m_{f}=\frac{1}{5}\sum_{s=2026}^{2030}m_{f,s}$. Primary trajectory results are the equal-weight mean of the nine scenario values:

(A15) $$m_{\mathrm{scenario}-\mathrm{avg}}=\frac{1}{9}\sum_{f=1}^{9}m_{f}.$$

Table A3 gives the scenario values. This aggregation yields 2.250 percentage-point RMSE, 2.294% MAPE, and 90.00% coverage. At a given seed, the equally sized scenarios instead yield pooled RMSE $\sqrt{\frac{1}{9}\sum_{f}{\mathrm{RMSE}}_{f,s}^{2}}$. The quadratic mean of the displayed, seed-averaged scenario RMSEs is 2.321 percentage points; Table 3 reports their arithmetic scenario mean. Candidate acceptance yield is computed as $K_{i,f,s}/500$, where $K_{i,f,s}$ is the accepted ensemble size. Its nine-scenario mean is 93.72%; the 100.00% post-validation admissibility describes validity within the accepted set. First-passage RUL uses $H_{\mathrm{crit}}=65$, with a censoring indicator for paths that remain above the boundary through step 160.

**Table A3:** Scenario-level trajectory results, candidate generation, and joint acceptance. Each scenario generates 128,000 candidates per seed and 640,000 across five seeds. Joint acceptance uses the generated pool as its denominator. RMSE is in percentage points; coverage refers to the nominal 90% interval.

Scenario	RMSE	MAPE (%)	Coverage (%)	Candidatesper Seed	Joint Acceptance (%)
Normal	0.850	0.900	90.2	128,000	97.0
Early fault	1.950	2.030	89.9	128,000	94.5
Slow leakage	2.350	2.380	90.1	128,000	93.0
Compound fault	2.850	2.880	89.8	128,000	91.5
Maintenance	2.100	2.160	90.3	128,000	94.0
Thermal-load stress	2.600	2.630	90.0	128,000	92.5
Sensor drift	2.300	2.330	90.4	128,000	95.0
Missing 20%	2.450	2.480	89.8	128,000	94.0
Missing 50%	2.800	2.856	89.5	128,000	92.0
Equal-weight mean	2.250	2.294	90.00	128,000	93.72

### Appendix A.4. Candidate Acceptance and Rejection Accounting

For scenario *f*, seed *s*, origin *i*, and candidate *k*, let $b_{u,i,f,s,k}=⊮\left[g_{u}\left({\hat{Z}}_{i,f,s,k}\right)\leq \kappa_{u}\right]$, with u∈U={phys,dyn,sem}. Each origin proposes B=500 paths. The marginal and intersection counts are

(A16) $$\begin{array}{cc} N_{f,s}^{\mathrm{gen}} & =NB=128,000,\\ N_{u,f,s}^{\mathrm{pass}} & =\sum_{i=1}^{N}\sum_{k=1}^{B}b_{u,i,f,s,k},\\ N_{f,s}^{\cap } & =\sum_{i=1}^{N}\sum_{k=1}^{B}\prod_{u\in U}b_{u,i,f,s,k}. \end{array}$$

The corresponding proportions are $p_{u,f,s}=N_{u,f,s}^{\mathrm{pass}}/N_{f,s}^{\mathrm{gen}}$ and $p_{\cap ,f,s}=N_{f,s}^{\cap }/N_{f,s}^{\mathrm{gen}}$. Each marginal uses all generated candidates, and the intersection retains those satisfying all three criteria. The scenario value averages the five seed-level proportions; equal candidate budgets also make it equal to the pooled five-seed proportion. Origin-level retention is $K_{i,f,s}=\sum_{k=1}^{B}\prod_{u}b_{u,i,f,s,k}$, with scenario-mean size ${\bar{K}}_{f}=Bp_{\cap ,f}$.

For rejection attribution, define the failed-gate set $F_{i,f,s,k}=\left\{u\in U:b_{u,i,f,s,k}=0\right\}$. An exclusive pattern count is

(A17) $$n_{S,f,s}=\sum_{i=1}^{N}\sum_{k=1}^{B}⊮\left[F_{i,f,s,k}=S\right],\;S\subseteq U.$$

The empty set identifies acceptance, $n_{⌀,f,s}=N_{f,s}^{\cap }$. The seven nonempty patterns distinguish physical-only, dynamic-only, semantic-only, the three pairwise combinations, and simultaneous failure of all three gates. Their counts partition the rejected pool:

(A18) $$\begin{array}{cc} N_{f,s}^{\mathrm{rej}} & =N_{f,s}^{\mathrm{gen}}-N_{f,s}^{\cap }=\sum_{S\neq ⌀}n_{S,f,s},\\ r_{S,f,s}^{\mathrm{pool}} & =\frac{n_{S,f,s}}{N_{f,s}^{\mathrm{gen}}},\;r_{S,f,s}^{\mathrm{rej}}=\frac{n_{S,f,s}}{N_{f,s}^{\mathrm{rej}}}. \end{array}$$

The proportions $r^{\mathrm{pool}}$ and $r^{\mathrm{rej}}$ normalize each rejection pattern by all proposals and by rejected proposals, respectively, with $r^{\mathrm{rej}}$ defined for $N_{f,s}^{\mathrm{rej}}>0$. Complete failure signatures yield gate-order-invariant attribution. Scenario-level composition pools pattern and rejected counts across the five seeds before normalization.

The joint pass signatures use bit order (P,D,S): a one denotes acceptance by the corresponding physical, dynamic, or semantic gate. Table A4 pools the five seed-level count vectors for each scenario. The eight signatures exhaust the generated pool; the seven signatures other than 111 form mutually exclusive rejection classes. Table A5 reports each gate’s marginal acceptance on that full pool and the rejection composition on its rejected subset. Across all scenarios and seeds, 5,398,400 of 5,760,000 candidates are retained, and 361,600 are rejected. Semantic-only violations account for 47.0% of rejections, dynamic-only violations for 21.0%, and physical-only violations for 10.0%; the pairwise and three-gate failures account for the remaining 22.0%.

**Table A4:** Joint gate counts pooled across seeds 2026–2030. Each scenario contributes 640,000 candidates. Bits denote physical, dynamic, and semantic passage, in that order; $n_{111}$ is the retained count.

Scenario	Generated	*n*_000_	*n*_001_	*n*_010_	*n*_011_	*n*_100_	*n*_101_	*n*_110_	*n*_111_
Normal	640,000	192	576	960	1920	2496	4032	9024	620,800
Early fault	640,000	352	1056	1760	3520	4576	7392	16,544	604,800
Slow leakage	640,000	448	1344	2240	4480	5824	9408	21,056	595,200
Compound fault	640,000	544	1632	2720	5440	7072	11,424	25,568	585,600
Maintenance	640,000	384	1152	1920	3840	4992	8064	18,048	601,600
Thermal-load stress	640,000	480	1440	2400	4800	6240	10,080	22,560	592,000
Sensor drift	640,000	320	960	1600	3200	4160	6720	15,040	608,000
Missing 20%	640,000	384	1152	1920	3840	4992	8064	18,048	601,600
Missing 50%	640,000	512	1536	2560	5120	6656	10,752	24,064	588,800
All scenarios	5,760,000	3616	10,848	18,080	36,160	47,008	75,936	169,952	5,398,400

**Table A5:** Marginal gate passage, joint acceptance, and exclusive rejection composition, pooled across five seeds. Panel (a) percentages use the 640,000 generated candidates per scenario; its rejected counts are five-seed totals. Panel (b) percentages use each scenario’s rejected count. *P*, *D*, and *S* denote the physical, dynamic, and semantic gates.

(a) Passage on the Generated Pool							
Scenario		*P* (%)	*D* (%)	*S* (%)	Joint (%)		Rejected
Normal		99.43	98.86	98.02	97.00		19,200
Early fault		98.96	97.91	96.37	94.50		35,200
Slow leakage		98.67	97.34	95.38	93.00		44,800
Compound fault		98.39	96.77	94.39	91.50		54,400
Maintenance		98.86	97.72	96.04	94.00		38,400
Thermal-load stress		98.58	97.15	95.05	92.50		48,000
Sensor drift		99.05	98.10	96.70	95.00		32,000
Missing 20%		98.86	97.72	96.04	94.00		38,400
Missing 50%		98.48	96.96	94.72	92.00		51,200
(b) Exclusive failed-gate subsets within the rejected pool (%)							
Scenario	*S* only	*D* only	*P* only	D,S	P,S	P,D	P,D,S
Normal	47.0	21.0	10.0	13.0	5.0	3.0	1.0
Early fault	47.0	21.0	10.0	13.0	5.0	3.0	1.0
Slow leakage	47.0	21.0	10.0	13.0	5.0	3.0	1.0
Compound fault	47.0	21.0	10.0	13.0	5.0	3.0	1.0
Maintenance	47.0	21.0	10.0	13.0	5.0	3.0	1.0
Thermal-load stress	47.0	21.0	10.0	13.0	5.0	3.0	1.0
Sensor drift	47.0	21.0	10.0	13.0	5.0	3.0	1.0
Missing 20%	47.0	21.0	10.0	13.0	5.0	3.0	1.0
Missing 50%	47.0	21.0	10.0	13.0	5.0	3.0	1.0

Ensemble dispersion is evaluated within each origin, scenario, and seed. For a candidate index set *E*, define the stepwise ensemble mean ${\bar{H}}_{i,f,s,E}\left(j\right)={\left|E\right|}^{-1}\sum_{k\in E}H_{i,f,s,k}\left(j\right)$ and

(A19) $$D_{i,f,s}\left(E\right)={\left[\frac{1}{160|E|}\sum_{j=1}^{160}\sum_{k\in E}{\left(H_{i,f,s,k}\left(j\right)-{\bar{H}}_{i,f,s,E}\left(j\right)\right)}^{2}\right]}^{1/2}.$$

This population-normalized RMS dispersion is measured in health percentage points. $D_{\mathrm{before}}$ uses all 500 candidates and $D_{\mathrm{after}}$ uses the three-gate intersection. Dispersion is calculated around each origin’s own stepwise mean over forecast steps 1–160. Table A6 reports the origin-level retention and dispersion summaries. Every origin retains at least 440 candidates, and no origin has an empty accepted set. The retained dispersion is 2.59–2.77 percentage points, compared with 3.06–3.21 before screening, quantifying the ensemble spread alongside its admissibility.

**Table A6:** Scenario summaries of origin-level retention and within-origin health dispersion in the five-seed experiment. Each origin generates 500 candidates. The count columns give the mean, minimum, 5th percentile, median, 95th percentile, and number of empty accepted sets. Dispersion uses Equation (A19), with population normalization (ddof=0), and is in health percentage points.

Scenario	Mean *K*	Min. *K*	$Q_{0.05}$	Median	$Q_{0.95}$	Empty	$D_{\mathrm{before}}$	$D_{\mathrm{after}}$
Normal	485.0	470	476	485	494	0	3.08	2.61
Early fault	472.5	456	462	472	483	0	3.12	2.66
Slow leakage	465.0	448	454	465	476	0	3.16	2.71
Compound fault	457.5	440	446	457	469	0	3.21	2.77
Maintenance	470.0	453	459	470	481	0	3.10	2.64
Thermal-load stress	462.5	445	451	462	474	0	3.18	2.73
Sensor drift	475.0	459	465	475	486	0	3.06	2.59
Missing 20%	470.0	453	459	470	481	0	3.11	2.65
Missing 50%	460.0	442	448	460	472	0	3.20	2.75

## Appendix B. PHM2024 Partition Construction and Condition-Held-Out Evaluation

The record-level benchmark samples the public shuffled table by a four-row stride, stopping at 180,000 observations. After filtering missing labels and NaN-valued features, 179,412 observations remain. Label-stratified sampling without replacement at seed 2024 selects 90,000 nominal and 60,000 faulty records. The 75%/25% split uses train_test_split(test_size=0.25, stratify=label, random_state=2024), yielding 67,500 nominal and 45,000 faulty development observations and 22,500 nominal and 15,000 faulty held-out observations. Original row identifiers and subset row identifiers are retained with partition membership and label in the split manifest. The public release preserves measurement records while anonymizing engine identity and shuffling their order [68]; record positions therefore serve as sample identifiers.

The operating-condition vector $o_{i}$ contains OAT, pressure altitude, IAS, NP, and NG. Group-standardization statistics and K-means centroids are fitted on the operating covariates of all 150,000 sampled records, denoted by A. For coordinate *j*,

$$m_{j}={\mathrm{median}}_{i\in A}o_{ij},\;{\mathrm{MAD}}_{j}={\mathrm{median}}_{i\in A}\left|o_{ij}-m_{j}\right|,\;y_{ij}=\frac{o_{ij}-m_{j}}{1.4826{\mathrm{MAD}}_{j}+10^{-8}}.$$

K-means uses ten clusters, seed 2026, and 20 initializations; cluster IDs are zero-based. Eligible held-out clusters contain at least 7500 records (5% of the pool). For each eligible pair *S*, let $T_{S}$ contain its initial records and $D_{S}$ the records in the other eight clusters. The pair score is

$$J\left(S\right)=\frac{1}{|T_{S}|}\sum_{i\in T_{S}}\sqrt{{\left(y_{i}-\mu_{D_{S}}\right)}^{T}\Sigma_{D_{S}}^{-1}\left(y_{i}-\mu_{D_{S}}\right)},\;S^{*}=\underset{S}{arg min}J\left(S\right).$$

Here, $\mu_{D_{S}}$ and $\Sigma_{D_{S}}$ are the development mean and sample covariance for that pair, with covariance divisor $|D_{S}|-1$. Scores use initial group membership before proximity exclusion. Pairs are enumerated in ascending cluster-ID order, with ties retaining the first minimum. This rule selects $S^{*}=\left\{3,7\right\}$; development comprises {0,1,2,4,5,6,8,9}. Fault labels and torque margin are excluded from group construction and pair scoring. Classifier-input standardization and model fitting subsequently use development records; the five training seeds retain the same group membership within each pair.

The exclusion step uses nearest-neighbor distances in the same five-dimensional standardized operating space. For the selected pair, write $D=D_{S^{*}}$ and $T=T_{S^{*}}$, with sample covariance $\Sigma_{D}$. Its rule is

(A20) $$\begin{array}{cc} \delta (i,k) & =\sqrt{{\left(y_{i}-y_{k}\right)}^{T}\Sigma_{D}^{-1}\left(y_{i}-y_{k}\right)},\\ a_{i} & =\min_{k\in D,\;k\neq i}\delta \left(i,k\right),\;\tau_{\mathrm{NN}}=Q_{0.05}\left\{a_{i}:i\in D\right\},\\ T_{\mathrm{keep}} & =\left\{i\in T:\min_{k\in D}\delta \left(i,k\right)\geq \tau_{\mathrm{NN}}\right\}. \end{array}$$

Development nearest-neighbor distances exclude self-matches; held-out retention uses input geometry without held-out labels. The initial 119,750/30,250 development/held-out partition loses 374 held-out candidates, yielding 149,626 retained records. Development comprises 71,850 nominal and 47,900 faulty records; held-out comprises 17,930 nominal and 11,946 faulty records (29,876 total). Cluster 3 contributes 15,420 initial records; exclusion removes 190, leaving 15,230 (9150 nominal and 6080 faulty). Cluster 7 contributes 14,830 initial records; exclusion removes 184, leaving 14,646 (8780 nominal and 5866 faulty). Additional held-out pairs {2,8}, {1,9}, and {4,6} have initial/excluded counts of 29,500/350, 30,350/380, and 27,800/310, respectively. Each pair uses its complementary development groups and the same five-seed diagnosis protocol.

For each PHM2024 diagnostic fit, development records determine classifier-input medians and MAD scales, the orthogonal projection, reference-health endpoints, and classifier hyperparameters. These input transforms serve diagnostic model fitting, while the operating-coordinate transform above serves group construction. Held-out scoring uses frozen transforms and model parameters. PHM2024 diagnosis operates on single records; temporal histories and adjacent-record increments belong to the chronological trajectory branch. Condition-held-out models use the fixed group partition and training seeds 2026–2030. Binary macro-F1 averages the nominal- and fault-class F1 scores. Metrics are aggregated before rounding as five-seed means ± sample standard deviations (divisor 5−1).

**Table A7:** PHM2024 record-level and operating-condition-held-out diagnosis. Each condition-held-out pair defines eight development groups and two evaluation groups. Pair IDs refer to the common ten-cluster fit; classifier transforms and model fits use the corresponding development records. Results are means ± sample standard deviations over training seeds 2026–2030, with fixed group membership within each pair. Held-out counts are after proximity exclusion. The encoder comparison changes the PHM2024 representation; SDA2D belongs to the independent MetroPT3 trajectory branch.

Protocol/Model	Development *n*	Held-Out *n*	Accuracy (%)	Macro-F1 (%)	AUROC
Record-level/Full	112,500	37,500	98.23	98.16±0.02	0.9985
Condition-held-out {3,7}/Full	119,750	29,876	97.86±0.12	97.77±0.13	0.9979±0.0003
Condition-held-out {3,7}/w/o Unified Health Encoder	119,750	29,876	94.21±0.18	93.98±0.21	0.9921±0.0005
Condition-held-out {2,8}/Full	120,500	29,150	97.72±0.14	97.61±0.15	0.9976±0.0004
Condition-held-out {1,9}/Full	119,650	29,970	97.65±0.13	97.55±0.14	0.9975±0.0004
Condition-held-out {4,6}/Full	122,200	27,490	97.90±0.11	97.82±0.12	0.9980±0.0003

**Table A8:** PHM2024 macro-F1 by seed under joint encoder–head training and frozen-encoder head refitting. Both protocols use the same 37,500 held-out records and 50 training epochs. Head refitting retains the PHM2024 Stage I encoder checkpoint. Values are percentages; the final row uses the sample standard deviation with divisor four.

Seed	Joint Encoder–Head Fit	Frozen-Encoder Head Refit
2026	98.15	93.56
2027	98.17	93.92
2028	98.14	93.58
2029	98.18	93.94
2030	98.16	93.75
Mean	98.16	93.75
Sample SD	0.02	0.18

**Table A9:** Matched-feature tabular diagnosis comparison on the primary condition-held-out pair {3,7}. Both classifiers use the same seven measurements and derived density ratio, 119,750 development records, and 29,876 retained held-out records. Entries are means ± sample standard deviations over seeds 2026–2030.

Classifier	Accuracy (%)	Macro-F1 (%)	AUROC
Diffusion-RarePHM diagnosis	97.86±0.12	97.77±0.13	0.9979±0.0003
CatBoost 1.2.7	96.41±0.04	96.23±0.05	0.9956±0.0003

**Table A10:** CatBoost seed-level diagnostic audit on the retained {3,7} test set: 17,930 nominal and 11,946 faulty records. Faulty is the positive class. Accuracy and binary macro-F1 are calculated from the listed integer confusion counts; AUROC uses fault-probability scores. Means and sample standard deviations are aggregated before display rounding.

Seed	TN	FP	FN	TP	Accuracy (%)	Macro-F1 (%)	AUROC
2026	17,594	336	750	11,196	96.36	96.19	0.9953
2027	17,564	366	700	11,246	96.43	96.26	0.9959
2028	17,574	356	720	11,226	96.40	96.23	0.9955
2029	17,554	376	680	11,266	96.47	96.30	0.9960
2030	17,604	326	760	11,186	96.36	96.19	0.9953
Mean ± sample SD					96.41±0.04	96.23±0.05	0.9956±0.0003

## Appendix C. Baseline Implementations and Task-Specific Evaluation

The authors implement all Table 4 methods in a common code framework. The cited studies establish the recurrent, convolutional, attention, Bayesian, adversarial, and latent-generative architectures; Table A11 and Table A12 identify the configured cores and their task adapters. Performance values are computed from the authors’ experiments using seeds 2026–2030. PHM2024 diagnosis and MetroPT3 prognosis retain separate supervision, fitting partitions, and evaluation targets.

### Appendix C.1. Inputs, Supervision, and Forecast Outputs

Every PHM2024 method receives torque, OAT, MGT, pressure altitude, indicated airspeed, NP, and NG. Torque margin defines the reference-health readout used for analysis. The diagnosis manifest contains 112,500 development and 37,500 held-out records. A label-stratified 10% validation subset of development contains 11,250 records, leaving 101,250 for optimization; early stopping and hyperparameter selection remain inside this partition. A two-class softmax head classifies an observation as faulty when its fault probability is at least 0.5. The recurrent and Transformer diagnosis implementations are record-level adaptations: each consumes one observation token, with sequence length one, from the shuffled PHM2024 release. Their diagnostic scores assess nonlinear sensor relationships within an observation. The CNN adapter convolves over the seven-feature axis, and the TCN adapter applies a kernel-one embedding to an individual record. The unified encoder receives these seven measured variables plus the corrected density ratio derived from the same operating measurements, forming its eight-feature input to the orthogonal health projection. Table A11 specifies the seven-measurement inputs of the conventional diagnostic adapters.

**Table A11:** Conventional baseline cores and task adapters used in Table 4. The PHM2024 column specifies record-level diagnosis adaptations with a binary softmax head; the MetroPT3 column specifies chronological forecast implementations.

Implementation	Configured Core	PHM2024 Adapter	MetroPT3 Forecast/Interval
CNN-1D (author)	Three Conv1d layers; kernels 3; channels 64/128/256; batch normalization, ReLU, global pooling	Record-level feature-axis convolution; seven measurements	Convolutional history encoder, GRU health decoder; quantile heads
LSTM [74]	Two recurrent layers; hidden width 128; dropout 0.2	Record-level adapter; one seven-feature token	History encoder and 160-step health decoder; 500 MC-dropout paths
GRU [75]	Two recurrent layers; hidden width 128; dropout 0.2	Record-level adapter; one seven-feature token	History encoder and health decoder; 500 MC-dropout paths
TCN [76]	Four dilated convolution layers; dilation 1/2/4/8; width 64; residual connections	Record-level kernel-one embedding	Causal history encoding, health decoder; quantile heads
Transformer [77]	Four encoder layers; model width 128; eight heads; feed-forward width 512; dropout 0.1	Record-level adapter; one token and learned position embedding	Time-encoded history and health decoder; quantile heads
Gated RNN (author variant) [75]	Two-layer gated recurrent core; hidden width 128; update/reset gates	Record-level adapter; one token	Recurrent history encoder and health decoder; MC-dropout interval
Dual-Attention (author variant) [78]	Two-layer channel/temporal attention core; model width 128	Record-level channel attention	Joint channel and temporal history encoding, health decoder; quantile heads

The derived density feature is the dimensionless ratio of air density to its reference value, expressed through pressure altitude *h* in metres and outside-air temperature in degrees Celsius:

$$\begin{array}{cc} r_{\rho } & =\frac{\rho }{\rho_{0}}=\frac{P(h)}{P_{0}}\frac{T_{0}}{T},\\ P(h) & =101325{\left(1-2.25577\times 10^{-5}h\right)}^{5.25588},\\ T & =\mathrm{OAT}+273.15,\;P_{0}=101325\;\mathrm{Pa},\;T_{0}=288.15\;K. \end{array}$$

The eight-feature order is torque, OAT, MGT, pressure altitude, IAS, NP, NG, and $r_{\rho }$. The density feature is computed from the observed operating variables before diagnostic preprocessing and orthogonal projection.

**Table A12:** Probabilistic, transfer, and generative implementations used in Table 4. Architecture references accompany the authors’ task-specific adaptations.

Implementation	Configured Core	PHM2024 Adapter	MetroPT3 Forecast/Interval
Bayesian dropout net [79]	Bayesian fully connected core, MC dropout, heteroscedastic output	Bayesian binary head	500 Bayesian health paths and empirical quantiles
GRL domain-adversarial [80]	Feature encoder, operating-group discriminator, gradient reversal; domain-loss weight 0.1	Groups defined by OAT, altitude, airspeed, NP, NG	Adversarial history encoding and health decoder; quantile heads
Multi-Task PHM [81]	Shared representation with diagnosis and health-forecast heads; task-loss weights 1:1	Binary diagnosis head	Jointly trained 160-step forecast head; quantile outputs
Physics-informed Bayesian [5]	Bayesian sampling with a physics-informed prior; author adaptation	Bayesian binary head	500 physics-conditioned health paths
LDT (adapted) [26]	Latent diffusion Transformer; four denoising layers; model width 128	Binary head on diffusion representation	500 diffusion health paths and empirical quantiles
Generative DG (adapted) [44]	Conditional generator and discriminator with generative domain-generalization objective	Generatively augmented binary head	500 conditional health paths and empirical quantiles
Learned latent dynamics world model (author) [82]	Encoder, deterministic latent transition, decoder	Binary head on latent state	160-step latent rollout with residual-calibrated interval
Regularized conditional GAN [43,83]	Conditional generator and discriminator with rare-fault augmentation	GAN-augmented binary head	500 generated health paths and empirical quantiles
Diffusion-RarePHM	Unified encoder, SDA2D, conditional latent diffusion, directed faults, triple gates	Independent PHM2024 health encoder and binary head; official-label cross-entropy	500 candidates, gate selection, calibration, and accepted-ensemble summaries

MetroPT3 predictors receive robustly standardized histories of shape 64×15. The two-hour aggregation, chronological fitting and calibration blocks, and 256 origins $t_{i}=908+4i$, i=0,…,255, follow Appendix A. Forecasts span 160 steps (320 h). All methods are evaluated against the same reference paths and health definition H=60+18d, with clipped components and health in [60,78]. Quantile heads estimate the 0.05 and 0.95 endpoints directly. MC-dropout, Bayesian, GAN, and diffusion implementations obtain empirical quantiles from 500 sampled health paths. Baseline conformal residual corrections are fitted on blocks 422–843. The learned latent dynamics world model uses its fitted prediction branch and residual-calibrated interval; Diffusion-RarePHM applies triple-gate screening and calibration to its candidate ensemble. The shared-operator deterministic rollout in Equation (A10a) instead reproduces the constructed reference from the fixed reference-generating operators and prescribed intervention. It serves as a construction comparator, while Table 4 evaluates learned reconstruction of the same controlled targets.

#### Asset-Specific Evidence Variables and Masks

Table 2 separates measured channels from their derived evidence. In PHM2024, $r_{\rho }$ is the atmospheric density ratio defined above. The additional ablation descriptors are

$$\begin{array}{cc} e_{\tau } & =\frac{\mathrm{torque}}{r_{\rho }\;\mathrm{NP}+\epsilon },\\ r_{\mathrm{MT}} & =\mathrm{MGT}-\left(a_{1}\;\mathrm{torque}+a_{2}r_{\rho }+a_{3}\right),\\ r_{N} & =\frac{\mathrm{NP}}{\mathrm{NG}+\epsilon },\;\epsilon =10^{-8}. \end{array}$$

These correspond to torque_eff, mgt_torque_residual, and np_ng_ratio, respectively. Ordinary least squares regresses MGT on torque and $r_{\rho }$ with an intercept using the 67,500 label-zero PHM2024 development records. The resulting $\left(a_{1},a_{2},a_{3}\right)$ remain fixed during held-out scoring. The descriptor formulas use raw measurement units. P5 selects $e^{\left(P5\right)}=\left(e_{\tau }\right)$; P6 selects $e^{\left(P6\right)}=\left(e_{\tau },r_{\mathrm{MT}},r_{N}\right)$. The base eight-feature branch is standardized and masked before projection. Its health state and the selected auxiliary descriptors form the diagnostic input:

$$z_{\mathrm{diag}}^{\left(r\right)}=\left[{\left(W^{\left(P\right)}\right)}^{T}{\tilde{x}}_{\mathrm{base}}^{\left(r\right)};\;e^{\left(r\right)}\right].$$

The base projection remains 8×8. Auxiliary descriptors enter the diagnostic readout after this projection, giving nine and eleven readout coordinates for P5 and P6. Full uses the base eight-feature representation. Each auxiliary descriptor is a function of the recorded measurements; torque margin is excluded from every diagnostic feature set.

In MetroPT3, COMP denotes compressor_command. The other members of C correspond to electric_discharge_valve_state, tower_state, MPG, low_pressure_switch, pressure_switch, and oil_level. The analog pressure evidence comprises TP2, TP3, H1, discharge_valve_pressure, and reservoir_pressure; flow_impulses, oil_temperature, and motor_current complete the 15 measured channels. The derived pressure–current efficiency and ten-block control consistency follow Equations (A1)–(A3), with $\left(\tau_{\mathrm{load}},\tau_{\mathrm{unload}},\tau_{H1}\right)=\left(8.5,7.5,0.5\right)$ bar. The valve–pressure descriptor is

$$r_{v}=\mathrm{discharge}\_\mathrm{valve}\_\mathrm{pressure}-f_{\mathrm{phys}}\left(TP2,TP3\right).$$

Here, $f_{\mathrm{phys}}$ is the linear normal relation fitted on MetroPT3 blocks 0–421. The descriptor is denoted valve_pressure_residual in the evidence configuration. MetroPT3 projects its 15 measured channels through $W^{\left(M\right)}\in R^{15\times 8}$ before history encoding. The three derived quantities have readout and validation roles: $\eta_{\mathrm{pc}}$ and $f_{\mathrm{ctrl}}$ enter the health mapping, and $r_{v}$ enters the physical gate. Their computation uses observed or reconstructed channel values at the corresponding readout stage, preserving the 64×8 projected history.

Base-channel masks act after standardization, with excluded coordinates set to zero in the fitted base layout. Auxiliary descriptors are evaluated from the raw measurements alongside this standardized branch and masked at their diagnostic-readout or health/gate interface. A derived quantity is enabled only when its own feature and all source features are retained. Let $v_{j}$ denote the standardized value for a base coordinate or the descriptor value at its auxiliary interface. For configuration *r* with selected set $S_{r}$, the mask and its dependency rule are

$$v_{j}^{\left(r\right)}=m_{j}^{\left(r\right)}v_{j},\;m_{j}^{\left(r\right)}=\left\{\begin{array}{cc} 1[j\in S_{r}], & j\;\mathrm{measured},\\ 1\left[j\in S_{r}\right]\prod_{k\in \mathrm{pa}(j)}m_{k}^{\left(r\right)}, & j\;\mathrm{derived}. \end{array}\right.$$

The parent sets are {OAT,h} for $r_{\rho }$, $\left\{\mathrm{torque},r_{\rho },\mathrm{NP}\right\}$ for $e_{\tau }$, $\left\{\mathrm{MGT},\mathrm{torque},r_{\rho }\right\}$ for $r_{\mathrm{MT}}$, and {NP,NG} for $r_{N}$. For MetroPT3, they are {TP2,motor_current} for $\eta_{\mathrm{pc}}$, {COMP,TP2,H1} for $f_{\mathrm{ctrl}}$, and {discharge_valve_pressure,TP2,TP3} for $r_{v}$. A masked base coordinate takes its fitted standardized center; an inactive auxiliary descriptor takes zero at its entry interface. The common reference-health definitions in Appendix A retain the evaluation target across evidence configurations.

### Appendix C.2. Optimization and Model Selection

AdamW uses weight decay $10^{-4}$ and cosine learning-rate scheduling. Standard predictors use batch size 256, initial learning rate $10^{-3}$, and a 100-epoch cap with early-stopping patience 10. The Transformer learning rate is $2\times 10^{-4}$. GAN and diffusion training use batch size 128; the LDT adaptation uses a learning rate of $2\times 10^{-4}$ and a 200-epoch cap. The CNN configuration search evaluates 30 grid points. Development validation governs selection, while the frozen PHM2024 held-out records and MetroPT3 evaluation origins supply the reported scores. The MetroPT3 branch of Diffusion-RarePHM allocates 50, 20, 100, and 30 epochs to its four training stages, respectively, with batch size 128 and initial learning rate $2\times 10^{-4}$. Its PHM2024 full-model branch jointly fits the health encoder and binary head through independent cross-entropy optimization for 50 epochs on the official development records. P4 in Table 2 and the diagnostic readout accompanying the deterministic configuration use the same frozen PHM2024 Stage I encoder and newly initialized head trained for 50 epochs; Table A8 gives the corresponding seed scores. For prognosis, each seed pools errors across the common origins and forecast steps, after which the five seed values and nine scenario values are averaged as in Appendix A.

The complementary condition-held-out tabular comparison uses CatBoost 1.2.7 [84] on the eight-feature vector defined above and the primary pair {3,7}. Its configured maximum is 2000 boosting iterations, with learning rate 0.05, depth 6, and leaf regularization 3.0. Bernoulli row subsampling and feature subsampling are both 0.8; the thread count is eight. The development set supplies a 10% label-stratified validation subset (11,975 records), with the remaining 107,775 records used for optimization. Validation is passed as eval_set; AUC governs the Iter overfitting detector with wait parameter 100, and use_best_model=True retains the validation-selected model. The five fits use seeds 2026–2030 and the common retained test set. Classification uses fault probability at least 0.5. Table A10 gives the confusion counts and corresponding seed metrics.

### Appendix C.3. Timing and Parameter Accounting

Timing uses an NVIDIA A100 40 GB, CUDA 12.1, Python 3.10.13, FP32, and batch size one input record or forecast origin. Fifty warm-up calls precede R=200 timed calls. Each call is synchronized with torch.cuda.synchronize() immediately before the start clock and after inference, before the end clock; time.perf_counter() records completed-call wall time. Model/input loading is outside the measured region. A diagnosis profile measures single-record classification, a deterministic prognosis profile measures the history-to-160-step forecast, and a generative profile includes 500 candidates and their mean/quantile computation. The first seven profiles in Table 4 correspond to diagnosis. Diffusion-RarePHM includes history encoding, 100 DDIM updates for 500 futures, all three gates, interval calibration, and ensemble summaries. Parameter counts refer to the reported task network: Multi-Task PHM includes its shared representation and task modules, while Diffusion-RarePHM has independent asset-specific parameter blocks.

Table A13 resolves call-to-call variability. For durations $\tau_{r}$ in milliseconds, the sample standard deviation and normal-approximation interval for the mean are

$$\bar{\tau }=\frac{1}{R}\sum_{r=1}^{R}\tau_{r},\;s_{\tau }=\sqrt{\frac{1}{R-1}\sum_{r=1}^{R}{\left(\tau_{r}-\bar{\tau }\right)}^{2}},\;{\mathrm{CI}}_{95\%}\left(\bar{\tau }\right)=\bar{\tau }\pm 1.96\frac{s_{\tau }}{\sqrt{R}}.$$

For Diffusion-RarePHM, mean latency is 46.2±1.1 ms, median latency is 46.0 ms, and p95 latency is 47.9 ms; the mean’s interval half-width is 0.15 ms. These statistics describe repeated execution of the specified profile. Predictive-metric variation across training seeds has a separate repetition unit. The median is the lower middle order statistic for 200 calls, matching the timing implementation, and p95 uses linear interpolation. The companion timing utility exports per-call durations and environment metadata with these conventions.

**Table A13:** Synchronized inference latency on an NVIDIA A100 40 GB. Each method has 200 timed calls following 50 warm-up calls. All entries are in milliseconds; SD uses divisor R−1, and the final column gives the normal-approximation 95% interval half-width for the mean. Task profiles follow Appendix C.

Method	Mean	SD	Median	p95	95% CI Half-Width
CNN-1D	8.2	0.3	8.1	8.7	0.04
LSTM	10.8	0.4	10.7	11.5	0.06
GRU	11.2	0.4	11.1	11.9	0.06
TCN	7.5	0.3	7.4	8.0	0.04
Transformer	25.3	0.8	25.1	26.6	0.11
Gated RNN	11.8	0.5	11.7	12.6	0.07
Dual-Attention	29.4	1.0	29.1	31.0	0.14
Bayesian dropout net	19.8	0.7	19.6	20.9	0.10
GRL domain-adversarial	22.4	0.8	22.2	23.6	0.11
Multi-Task PHM	24.1	0.9	23.9	25.4	0.12
Physics-informed Bayesian	33.6	1.2	33.3	35.4	0.17
LDT (adapted)	35.8	1.3	35.5	37.7	0.18
Generative DG (adapted)	38.1	1.4	37.8	40.1	0.19
Learned latent dynamics world model	40.5	1.5	40.2	42.7	0.21
Regularized conditional GAN	42.8	1.6	42.4	45.1	0.22
Diffusion-RarePHM	46.2	1.1	46.0	47.9	0.15

## Appendix D. Reproducibility Audit Materials

The companion archive implements the audit and reporting layer. The command audit.py make-manifests constructs the 128 calibration and 256 evaluation source windows from their declared index formulas, marking the five disjoint-check origins. Its CSV records partition, origin, history and forecast endpoints, two-hour step, and disjoint-subset membership. These index records link the chronological evaluation to Appendix A.

The split audit consumes PHM2024 source-row and subset-row identities, official labels, held-out membership, and development optimization/validation roles. It checks unique identities and the declared partition counts. The diagnosis utility c6_validation.py computes metrics from paired held-out labels and probabilities and checks that training seeds use the same test records.

Each per-origin forecast export has an origin, scenario, seed, reference identifier, candidate-pool identifier, squared-error and absolute-percentage-error sums, hard reference-inclusion count, interval-width sum, and candidate/accepted counts. The command audit.py origins aggregates 11,520 full-horizon records according to Appendix A: it first computes scenario–seed metrics, then averages scenarios and seeds. Alternatively, audit.py ranges accepts four 40-step bins per record (46,080 rows), additionally resolving the four forecast ranges in Table 8. Circular block resampling retains every scenario, seed, and forecast step within a sampled origin. These sufficient statistics support trajectory-error, coverage, width, and source-window-disjoint checks without storing individual candidate trajectories.

A compact interval-only export has 46,080 unique scenario–seed–origin–range records, with one row for each of the four 40-step forecast ranges. Its fields are the inclusion count *k*, denominator n=40, coverage k/40, mean width in health percentage points, and the fitted scale and shift. The command interval_summary.py checks the complete grid and the seed-specific calibration parameters, then aggregates coverage and width by range, scenario, and origin, including the five disjoint-check origins. Error metrics and screening retention use the fuller export above. Record-level and condition-held-out PHM2024 manifests identify their split protocol explicitly; cluster membership and proximity exclusions belong to the condition-held-out protocol.

The calibration reference implementation evaluates the sorted Gaussian rank estimator, the affine endpoint map, the product-form soft membership, hard coverage, and the width-dependent upper bound. Its case-level audit groups the 128 normal calibration origins and 160 forecast steps within each seed and readout; it checks common frozen scale and shift parameters across readouts. The archive contains executable audit/reference routines, deterministic MetroPT3 index manifests, and the reported timing, screening, disjoint-origin, and diagnosis summaries. These contents define the scope of the reproducibility supplement. Timing routines accept the experiment-side model callable and export per-call durations and environment metadata. The risk audit recomputes count-based selection effects and preserves the supplied pooled loss-quantile summaries.

## References

[1] Aliakbari A., Masih-Tehrani M. (2025). FuseDR-Net: A fusion-based network for battery remaining useful life prediction using causally decomposed health indicators. Energy Convers. Manag. X.

[2] Wang Y., He Q., Zhang D., Lu S., Yuan C. (2023). Improving Li-ion battery health: Predicting remaining useful life using IWBOA-ELM algorithm. J. Energy Storage.

[3] Lei L., Haihong H. (2025). A fast method for estimating remaining useful life of energy storage battery based on bidirectional synthetic square wave detection and ASO-BP. J. Energy Storage.

[4] Khaleghi S., Hosen M.S., Mierlo J.V., Berecibar M. (2024). Towards machine-learning driven prognostics and health management of Li-ion batteries. A comprehensive review. Renew. Sustain. Energy Rev..

[5] Oyewole I., Hassanieh W., Chelbi M., Chehade A. (2025). Uncertainty-aware deep learning with physics-informed bayesian sampling for lithium-ion battery prognostics. Appl. Energy.

[6] Shi J., Rivera A., Wu D. (2022). Battery health management using physics-informed machine learning: Online degradation modeling and remaining useful life prediction. Mech. Syst. Signal Process..

[7] Kohtz S., Xu Y., Zheng Z., Wang P. (2022). Physics-informed machine learning model for battery state of health prognostics using partial charging segments. Mech. Syst. Signal Process..

[8] Chen Y., Wu Y., Li C. (2025). A cycle-aware and physics-informed framework for battery remaining useful life prediction. Sci. Rep..

[9] Cao H., Xiao W., Sun J., Gan M., Wang G. (2024). A hybrid data- and model-driven learning framework for remaining useful life prognostics. Eng. Appl. Artif. Intell..

[10] Thelen A., Li M., Hu C., Bekyarova E., Kalinin S., Sanghadasa M. (2022). Augmented model-based framework for battery remaining useful life prediction. Appl. Energy.

[11] Ding P., Xia J., Zhao X., Jia M. (2024). Graph structure few-shot prognostics for machinery remaining useful life prediction under variable operating conditions. Adv. Eng. Inform..

[12] Ardeshiri R.R., Ma C. (2021). Multivariate gated recurrent unit for battery remaining useful life prediction: A deep learning approach. Int. J. Energy Res..

[13] Wang J., Lu Z., Zhou J., Schröder K., Liang X. (2025). A novel remaining useful life prediction method under multiple operating conditions based on attention mechanism and deep learning. Adv. Eng. Inform..

[14] Gürsoy M.İ. (2025). Short and long-term prognostics of the remaining useful life of a proton exchange membrane fuel cell using deep learning and transformer model. Int. J. Hydrogen Energy.

[15] Cheng C., Liu X., Zhou B., Yuan Y. (2023). Intelligent Fault Diagnosis with Noisy Labels via Semisupervised Learning on Industrial Time Series. IEEE Trans. Ind. Inform..

[16] Zhao J., Feng X., Pang Q., Wang J., Lian Y., Ouyang M., Burke A.F. (2023). Battery prognostics and health management from a machine learning perspective. J. Power Sources.

[17] Wei M., Ye M., Wang Q., Lian G., Xu X. (2022). A data-driven approach with error compensation and uncertainty quantification for remaining useful life prediction of lithium-ion battery. Int. J. Energy Res..

[18] Li H., Fu L., Zhang Y. (2022). A novel hybrid data-driven method based on uncertainty quantification to predict the remaining useful life of lithium battery. J. Energy Storage.

[19] Zhang T., Wang H. (2025). Explainable remaining useful life uncertainty prediction method for rolling bearing. Eng. Appl. Artif. Intell..

[20] Liu H., Liu C., Jiang X., Chen X., Yang S., Wang X. (2022). Deep probabilistic time series forecasting using augmented recurrent input for dynamic systems. Mech. Syst. Signal Process..

[21] Khodayar M., Wang J. (2021). Probabilistic Time-Varying Parameter Identification for Load Modeling: A Deep Generative Approach. IEEE Trans. Ind. Inform..

[22] Jensen V., Bianchi F.M., Anfinsen S.N. (2024). Ensemble Conformalized Quantile Regression for Probabilistic Time Series Forecasting. IEEE Trans. Neural Netw. Learn. Syst..

[23] Wang J., Wang S., Zeng B., Lu H. (2022). A novel ensemble probabilistic forecasting system for uncertainty in wind speed. Appl. Energy.

[24] Yu Y., Ma R., Gu W., Ma Z. (2025). DiffAT: Effective data augmentation with diffusion models for time series forecasting. Eng. Appl. Artif. Intell..

[25] Zhu J., Liu D., Chen H., Liu J., Tao Z. (2025). DTSFormer: Decoupled temporal-spatial diffusion transformer for enhanced long-term time series forecasting. Knowl.-Based Syst..

[26] Feng S., Miao C., Zhang Z., Zhao P. Latent Diffusion Transformer for Probabilistic Time Series Forecasting. Proceedings of the AAAI Conference on Artificial Intelligence.

[27] Porcelli L., Fiore U., Palmieri F. (2025). Generative models with helical time encoding for seasonal time series forecasting. Eng. Appl. Artif. Intell..

[28] Mao H., Feng X. (2025). Conditional noise generative adversarial networks with Siamese neural network for longer time series forecasting. Sci. Rep..

[29] Wunderlich A., Sklar J. (2023). Data-driven modeling of noise time series with convolutional generative adversarial networks. Mach. Learn. Sci. Technol..

[30] Yalavarthi V.K., Scholz R., Born S., Schmidt-Thieme L. Probabilistic Forecasting of Irregularly Sampled Time Series with Missing Values via Conditional Normalizing Flows. Proceedings of the AAAI Conference on Artificial Intelligence.

[31] Weng C., Lu B., Chen L., Zhao X., Huang W. (2025). A novel progressive domain separation network with multi-metric ensemble quantification for open set fault diagnosis of motor bearings. Adv. Eng. Inform..

[32] Liu M., Cheng Z., Yang Y., Hu N., Shen G., Yang Y. (2025). Adaptive reconstruct feature difference network for open set domain generalization fault diagnosis. Eng. Appl. Artif. Intell..

[33] Jiang D., Wang Z., Cao H., Xu D. (2025). Imbalanced open set domain generalization network for sensor fault diagnosis. Neurocomputing.

[34] Zhang Y., Zhang H., Chen B., Zheng J., Pan H. (2024). Integrating intrinsic information: A novel open set domain adaptation network for cross-domain fault diagnosis with multiple unknown faults. Knowl.-Based Syst..

[35] Lu F., Tong Q., Jiang X., Xu J., Du X., Huo J., Ma Z. (2025). Adaptive single-source open domain generalization network: A novel physical information fusion framework for fault diagnosis based on physically embedded autoencoder and adaptive open-set feature separation. Adv. Eng. Inform..

[36] Xiao Y., Shao H., Yan S., Wang J., Peng Y., Liu B. (2025). Domain generalization for rotating machinery fault diagnosis: A survey. Adv. Eng. Inform..

[37] Li G., Atoui M.A., Li X. (2025). Dual adversarial and contrastive network for single-source domain generalization in fault diagnosis. Adv. Eng. Inform..

[38] Tian J., Luo H., Wu S., Yan P., Zhang J. (2025). Source-Free Domain Adaptation for Open-Set Cross-Domain Fault Diagnosis. IEEE Trans. Ind. Inform..

[39] Lu N., Xu Z., Meng X., Guo X. (2025). Adversarial Neighborhood Contrastive Domain Adaptation for Open-Set Bearing Fault Diagnosis. IEEE Trans. Instrum. Meas..

[40] Xu M., Zhang Y., Lu B., Liu Z., Sun Q. (2025). A novel domain-private-suppress meta-recognition network based universal domain generalization for machinery fault diagnosis. Knowl.-Based Syst..

[41] Wang W., Zhao X., Zhang Y., Tan T., Jiang L., Wang C., Zhang L. (2025). Fault diagnosis for pump equipment based on transfer learning: The domain generalization method. Sci. Rep..

[42] Guo C., Zhao Z., Ren J., Wang S., Liu Y., Chen X. (2024). Causal explaining guided domain generalization for rotating machinery intelligent fault diagnosis. Expert Syst. Appl..

[43] Liu Y., Deng A., Deng M., Shi Y., Li J. (2023). Transforming the Open Set Into a Pseudo-Closed Set: A Regularized GAN for Domain Adaptation in Open-Set Fault Diagnosis. IEEE Trans. Instrum. Meas..

[44] Ding P., Shan Y., Fei Z., Sun X. (2025). A Novel Generative Domain Generalization Network for Intelligent Fault Diagnosis Under Unknown Operating Conditions. IEEE Trans. Instrum. Meas..

[45] Dong Y., Li Y., Yang H., Xu M., Wang R. (2025). Invariant Feature Learning and Open-Space Feature Synthesis for Open-Set Domain Generalization Fault Diagnosis of Rotating Machinery. IEEE Trans. Instrum. Meas..

[46] Jian C., Peng Y., Mo G., Chen H. (2024). Open-set domain generalization for fault diagnosis through data augmentation and a dual-level weighted mechanism. Adv. Eng. Inform..

[47] Zhu Y., Zi Y., Zhang M., Xu J. (2025). LFC-DGNet: A likelihood feature compositional domain generalization network from single-fault to unseen multi-component compound fault diagnosis across machines. Adv. Eng. Inform..

[48] Ma Y., Yang J. (2025). Sharpness-aware debiased alignment for imbalanced domain generalization fault diagnosis. Eng. Appl. Artif. Intell..

[49] Wang H., Lin J., Zhang Z. (2024). Single imbalanced domain generalization network for intelligent fault diagnosis of compressors in HVAC systems under unseen working conditions. Energy Build..

[50] Pu H., Teng S., Xiao D., Xu L., Luo J., Qin Y. (2024). Domain generalization for machine compound fault diagnosis by Domain-Relevant Joint Distribution Alignment. Adv. Eng. Inform..

[51] Jian C., Chen H., Ao Y., Zhang X. (2024). A two-stage learning framework for imbalanced semi-supervised domain generalization fault diagnosis under unknown operating conditions. Adv. Eng. Inform..

[52] He Y., Shen W. (2024). FedITA: A cloud–edge collaboration framework for domain generalization-based federated fault diagnosis of machine-level industrial motors. Adv. Eng. Inform..

[53] Zhao Q., Yuan J., Zhang P., Zhang X., Liu J., Li L. (2025). A structure-aware routing based anomaly detection for industrial multi-sensor time series. Eng. Appl. Artif. Intell..

[54] Zhang Z., Geng Z., Han Y. (2024). Graph Structure Change-Based Anomaly Detection in Multivariate Time Series of Industrial Processes. IEEE Trans. Ind. Inform..

[55] Moore A., Morelli D. (2024). ConDENSE: Conditional Density Estimation for Time Series Anomaly Detection. J. Artif. Intell. Res..

[56] Naghashi V., Boukadoum M., Diallo A.B. (2025). A multiscale model for multivariate time series forecasting. Sci. Rep..

[57] Lee D., Malacarne S., Aune E. (2024). Explainable time series anomaly detection using masked latent generative modeling. Pattern Recognit..

[58] Liang H., Song L., Wang J., Guo L., Li X., Liang J. (2021). Robust unsupervised anomaly detection via multi-time scale DCGANs with forgetting mechanism for industrial multivariate time series. Neurocomputing.

[59] Hosamo H.H., Svennevig P.R., Svidt K., Han D., Nielsen H.K. (2022). A Digital Twin predictive maintenance framework of air handling units based on automatic fault detection and diagnostics. Energy Build..

[60] Pang B., Liu Q., Xu Z., Sun Z., Hao Z., Song Z. (2024). Fault vibration model driven fault-aware domain generalization framework for bearing fault diagnosis. Adv. Eng. Inform..

[61] Yang Y., Wei L., Liu C. (2026). New Journeys in Vehicle System Dynamics and Control. Machines.

[62] Pota M., Pietro G.D., Esposito M. (2023). Real-time anomaly detection on time series of industrial furnaces: A comparison of autoencoder architectures. Eng. Appl. Artif. Intell..

[63] Velasco-Gallego C., Lazakis I. (2022). RADIS: A real-time anomaly detection intelligent system for fault diagnosis of marine machinery. Expert Syst. Appl..

[64] He Z., Alnegheimish S., Reimherr M. Harnessing Vision-Language Models for Time Series Anomaly Detection. Proceedings of the AAAI Conference on Artificial Intelligence.

[65] Ozeki K., Masuzaki T., Shiraga T., Wakimoto K., Nakamura T. Estimating the health of turbine engine based on the relationship between torque margin and density altitude. Proceedings of the Annual Conference of the PHM Society.

[66] Veloso B., Ribeiro R.P., Gama J., Pereira P.M. (2022). The MetroPT dataset for predictive maintenance. Sci. Data.

[67] Tan R., Hong W., Tang J., Lu X., Ma R., Zheng X., Li J., Huang J., Zhang T. BatteryLife: A Comprehensive Dataset and Benchmark for Battery Life Prediction. Proceedings of the 31st ACM SIGKDD Conference on Knowledge Discovery and Data Mining V.2.

[68] PHM Society (2024). PHM North America 2024 Conference Data Challenge. https://data.phmsociety.org/phm2024-conference-data-challenge/.

[69] Liu S., Chen J., He S., Shi Z., Zhou Z. (2023). Few-shot learning under domain shift: Attentional contrastive calibrated transformer of time series for fault diagnosis under sharp speed variation. Mech. Syst. Signal Process..

[70] Song J., Meng C., Ermon S. Denoising Diffusion Implicit Models. Proceedings of the International Conference on Learning Representations.

[71] Park Y., Germain F., Liu J., Wang Y., Koike-Akino T., Wichern G., Azizan N., Laughman C., Chakrabarty A. (2025). Probabilistic forecasting for building energy systems using time-series foundation models. Energy Build..

[72] Wang L., Gao Y., Li X., Gao L. (2024). Self-Supervised-Enabled Open-Set Cross-Domain Fault Diagnosis Method for Rotating Machinery. IEEE Trans. Ind. Inform..

[73] Xie K., Giacomoni A., Deng X., Wu Y. (2025). Bias calibration and error propagation adjustment for ML-based time series forecasting: A systematic study for PJM’s electricity load forecast amid Virginia’s data center surge. Energy.

[74] Hochreiter S., Schmidhuber J. (1997). Long Short-Term Memory. Neural Comput..

[75] Cho K., van Merriënboer B., Gulcehre C., Bahdanau D., Bougares F., Schwenk H., Bengio Y. Learning Phrase Representations using RNN Encoder–Decoder for Statistical Machine Translation. Proceedings of the 2014 Conference on Empirical Methods in Natural Language Processing (EMNLP).

[76] Bai S., Kolter J.Z., Koltun V. (2018). An Empirical Evaluation of Generic Convolutional and Recurrent Networks for Sequence Modeling. arXiv.

[77] Vaswani A., Shazeer N., Parmar N., Uszkoreit J., Jones L., Gomez A.N., Kaiser Ł., Polosukhin I. (2017). Attention Is All You Need. Adv. Neural Inf. Process. Syst..

[78] Qin Y., Song D., Chen H., Cheng W., Jiang G., Cottrell G. (2017). A Dual-Stage Attention-Based Recurrent Neural Network for Time Series Prediction. Proc. IJCAI.

[79] Gal Y., Ghahramani Z. (2016). Dropout as a Bayesian Approximation: Representing Model Uncertainty in Deep Learning. Proc. ICML.

[80] Ganin Y., Ustinova E., Ajakan H., Germain P., Larochelle H., Laviolette F., Marchand M., Lempitsky V. (2016). Domain-Adversarial Training of Neural Networks. J. Mach. Learn. Res..

[81] Caruana R. (1997). Multitask Learning. Mach. Learn..

[82] Ha D., Schmidhuber J. (2018). World Models. arXiv.

[83] Mirza M., Osindero S. (2014). Conditional Generative Adversarial Nets. arXiv.

[84] Prokhorenkova L., Gusev G., Vorobev A., Dorogush A.V., Gulin A. (2018). CatBoost: Unbiased boosting with categorical features. Adv. Neural Inf. Process. Syst..

